# Supplementary material for: Cell2fate infers RNA velocity modules to improve cell fate prediction
Source: Nat Methods. 2025 Mar 3;22(4):698–707. doi: 10.1038/s41592-025-02608-3 (PMC11978503; doi:10.1038/s41592-025-02608-3)
Supplement: Supplementary file 1 — Supplementary Figs. 1–28 and Notes 1–3 [file 41592_2025_2608_MOESM1_ESM.pdf]

nature methods

Supplementary information https://doi.org/10.1038/s41592-025-02608-3

# Cell2fate infers RNA velocity modules to improve cell fate prediction

In the format provided by the authors and unedited

## Supplementary Tables

**Supp. Table 1: Dentate Gyrus dataset CBDir metric for all cluster transitions and methods**
*The rows show the performance across different methods and the columns the scores in individual transitions. The last "Mean" column is an average across all individuals transitions.*

**Supp. Table 2: Pancreas dataset CBDir metric for all cluster transitions and methods**
*The rows show the performance across different methods and the columns the scores in individual transitions. The last "Mean" column is an average across all individuals transitions.*

**Supp. Table 3: Mouse Bone Marrow dataset CBDir metric for all cluster transitions and methods**
*The rows show the performance across different methods and the columns the scores in individual transitions. The last "Mean" column is an average across all individuals transitions.*

**Supp. Table 4: Erythroid Maturation dataset CBDir metric for all cluster transitions and methods**
*The rows show the performance across different methods and the columns the scores in individual transitions. The last "Mean" column is an average across all individuals transitions.*

**Supp. Table 5: Human Bone Marrow CBDir metric for all cluster transitions and methods**
*The rows show the performance across different methods and the columns the scores in individual transitions. The last "Mean" column is an average across all individuals transitions.*

**Supp. Table 6: Computation time for all methods on all datasets**
*The "GPU use" column indicates whether a method made use of a GPU or CPU and the "n_jobs" column indicates how many parallel jobs were used. For simplicity, only 1 job was used, but for CPU-based methods it can be expected that processing time decreases linearly with an increasing number of jobs used.*

**Supp. Table 7: Memory requirements of all methods on the largest dataset (Erythroid Maturation)**
*The "GPU use" column indicates whether a method made use of a GPU or CPU and the "n_jobs" column indicates how many parallel jobs were used. For simplicity, only 1 job was used, but it can be expected that the memory requirements increase linearly with an increasing number of jobs used.*

**Supp. Table 8: Time differences between clusters for all methods that infer a cell-specific time**
*The first column denotes the dataset, the second column the two cluster in the dataset for which the time difference was computed, where the cluster that is known to be the earlier one is listed first. The following columns show the Time Difference between clusters for cell2fate, as well as the CBDir score and confidence scores for comparison. The remaining columns show the time difference between clusters and the CBDir score for the pyroVelocity_model2 and UnitVelo methods. The last row indicates the number of correctly inferred transitions for each method and metric.*

**Supp. Table 9: Top module genes and TFs in the dentate gyrus dataset**
*For each module number the module genes and TFs with highest loadings are shown, followed by the enriched pathways in the "Terms Ranked" column. The last two columns show top module genes that overlap with known risk genes for Alzheimer's or Autism Spectrum Disorder (ASD).*

## Supplementary Figures

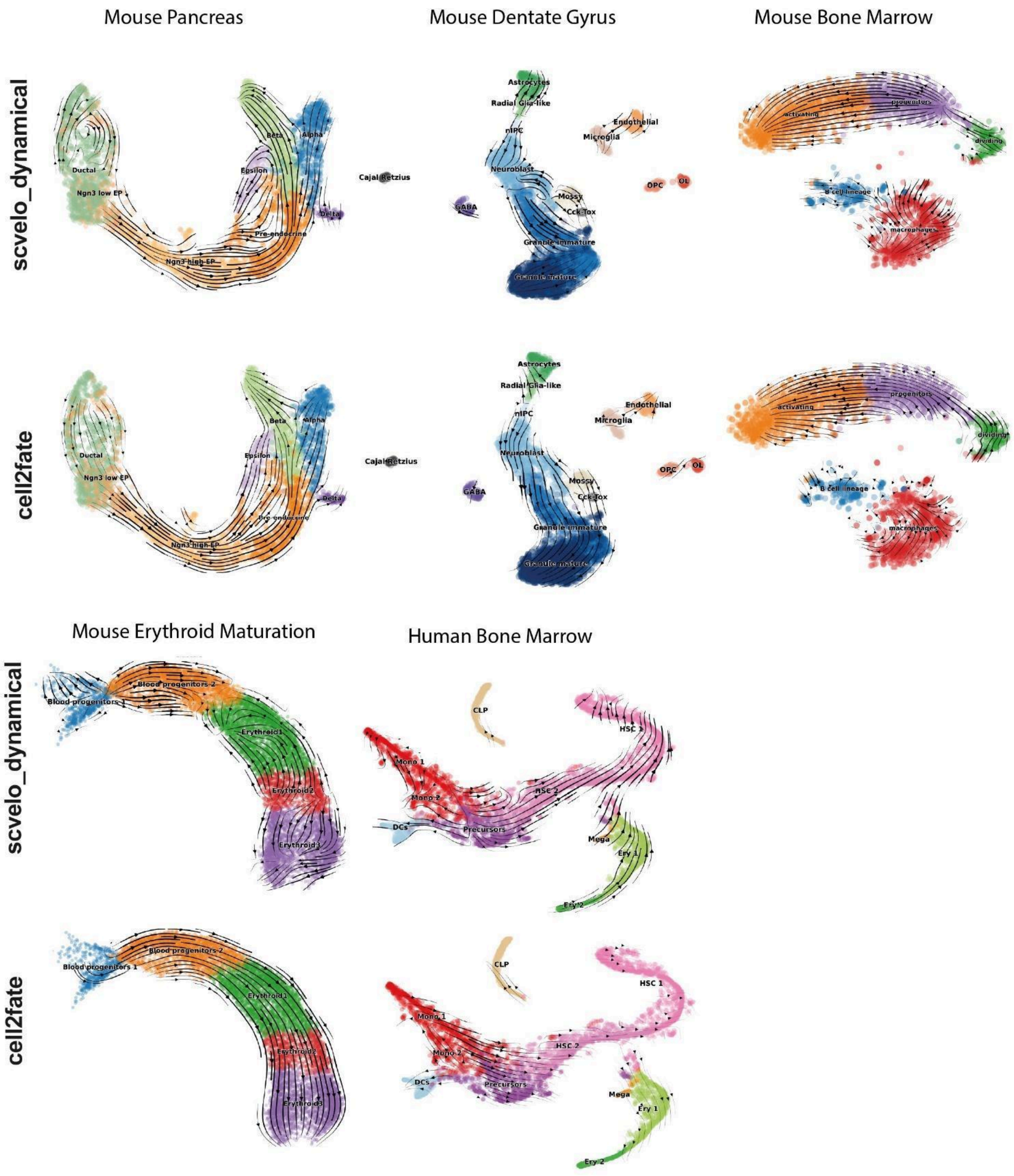


**Supp. Figure 1: all RNA velocity graph UMAP projections for scvelo_dynamical and cell2fate**
*Cell2fate more faithfully captures the expected differentiation trajectories, especially in challenging cases, such as mature granule neurons in the Dentate Gyrus dataset or complex transcription rate changes in the Erythroid Maturation and Human Bone Marrow datasets.*

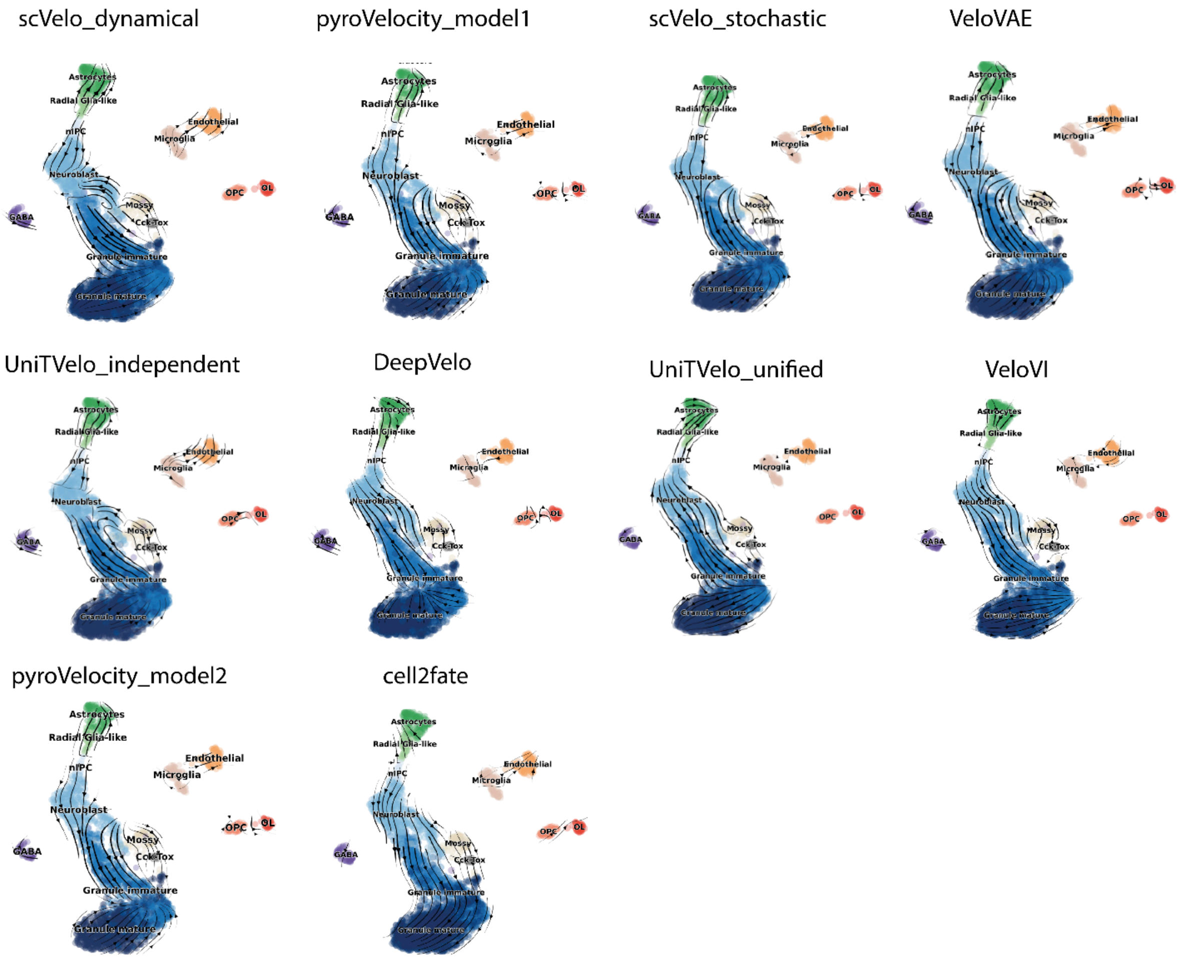


**Supp. Figure 2: velocity graph UMAPs for dentate gyrus data across all benchmarked methods**

*The dataset includes differentiation trajectories from Radial glia-like cells to Astrocytes, nIPC to mature granule neurons and OPC to Oligodendrocytes (OL). Cell2fate more faithfully captures the expected differentiation trajectories, especially in challenging cases, such as mature granule neurons.*

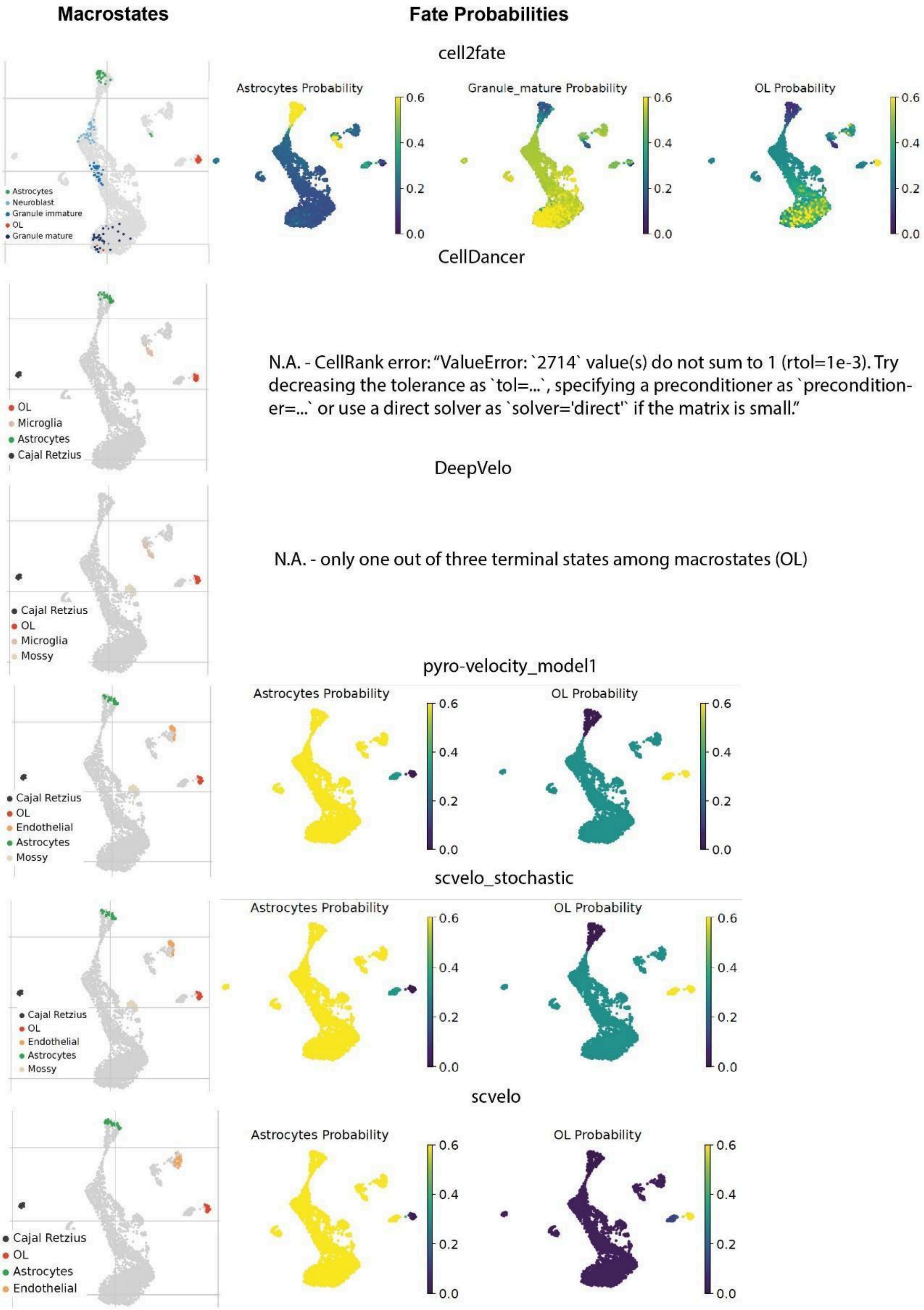


**Supp. Figure 3: CellRank Macrostates (left) and Fate Probabilities (right) for velocity graph inputs from 6 RNA velocity methods on the Dentate Gyrus dataset** *Fate probabilities for CellDancer could not be computed due to a CellRank error that is copied instead into the figure. cell2fate is the only method to detect the Granule mature macrostate.*

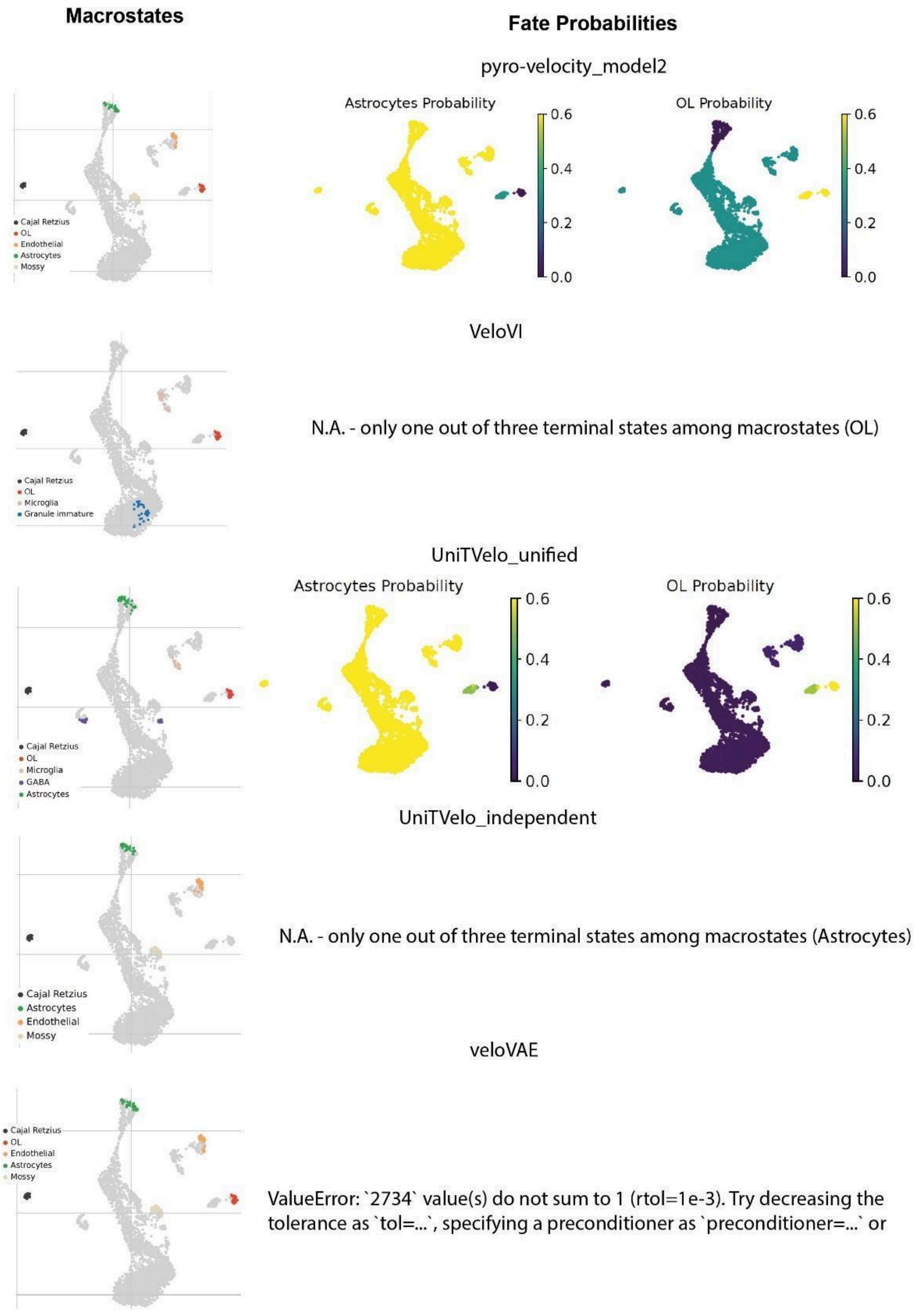


**Supp. Figure 4: CellRank Macrostates (left) and Fate Probabilities (right) for velocity graph inputs from 5 RNA velocity methods on the Dentate Gyrus dataset** *Fate probabilities for veloVAE could not be computed due to a CellRank error that is copied instead into the figure.*

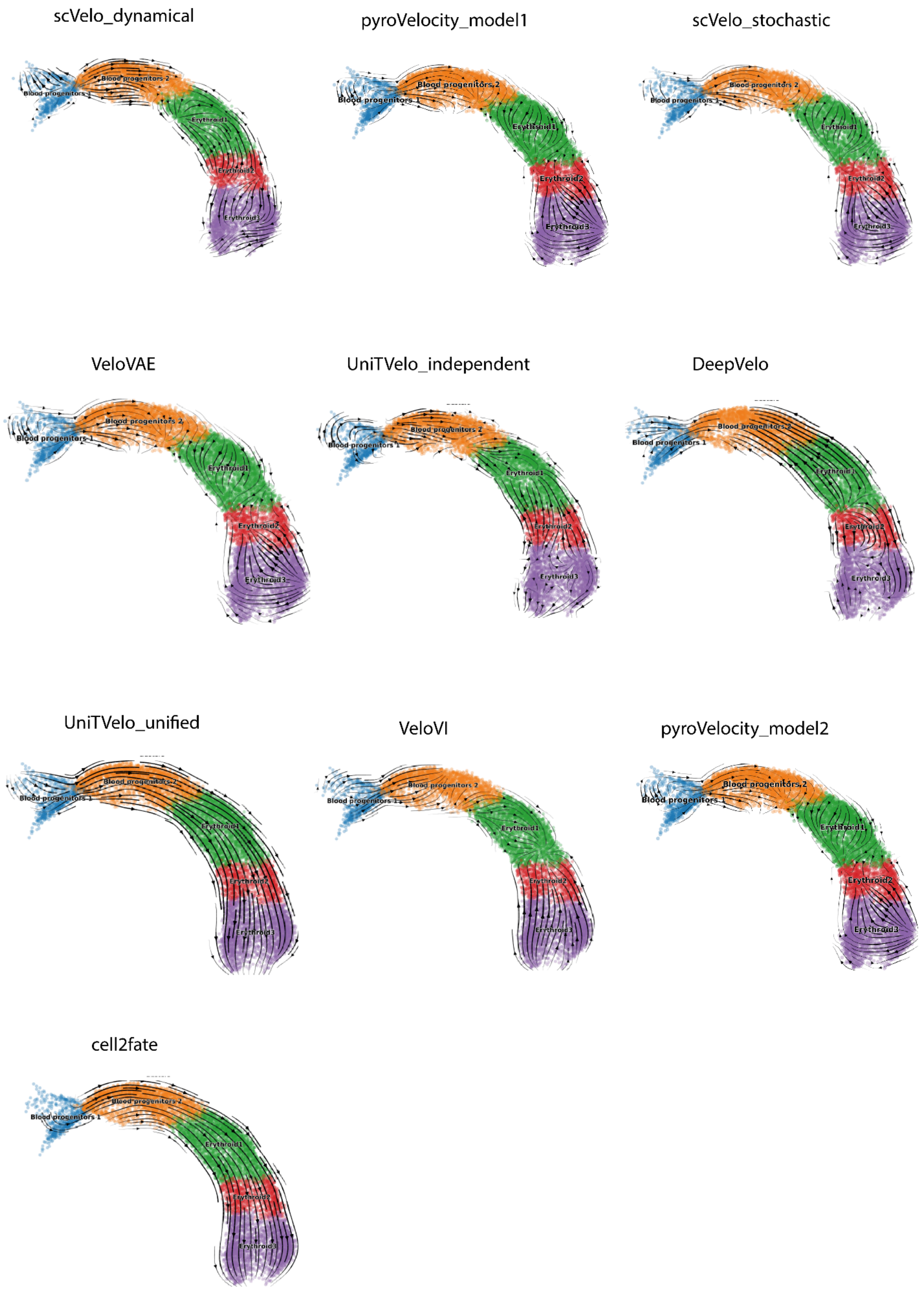


**Supp. Figure 5: velocity graph UMAPs for erythroid maturation data across all benchmarked methods** *The expected trajectory begins at Blood progenitors 1 and ends in Erythroid 3 cells. UniTVelo and cell2fate produce the expected velocity arrows throughout the trajectory.*

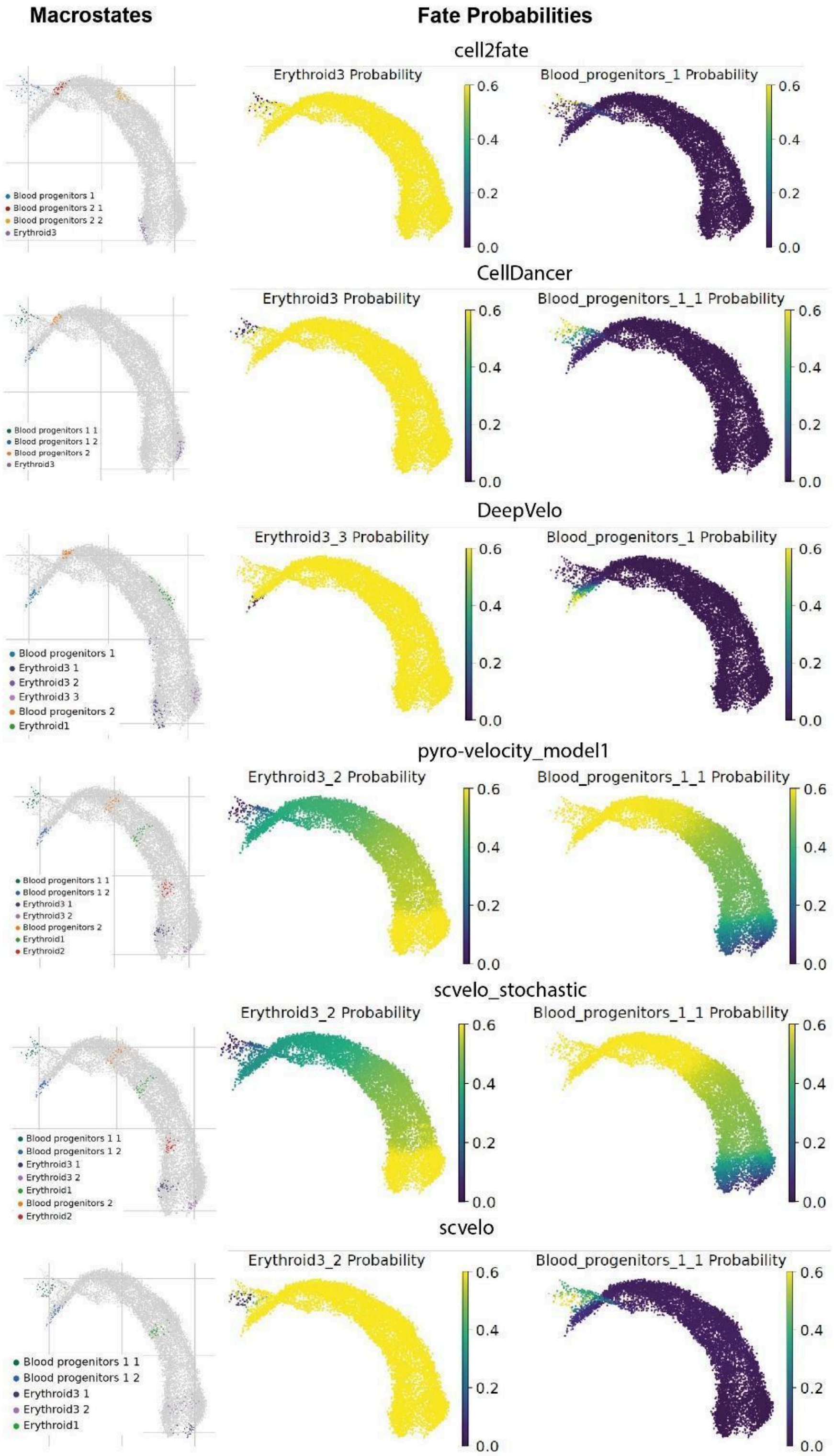


**Supp. Figure 6: CellRank Macrostates (left) and Fate Probabilities (right) for velocity graph inputs from 6 RNA velocity methods on the Erythroid Maturation dataset** *All methods identify macrostates both in progenitors and mature Erythroid cells, but some methods (pyro-velocity_model1, scvelo_stochastic) erroneously assign high fate probabilities to the progenitor state throughout the trajectory. Instead, the macrostate associated with the Erythroid 3 cluster should have a high probability throughout.*

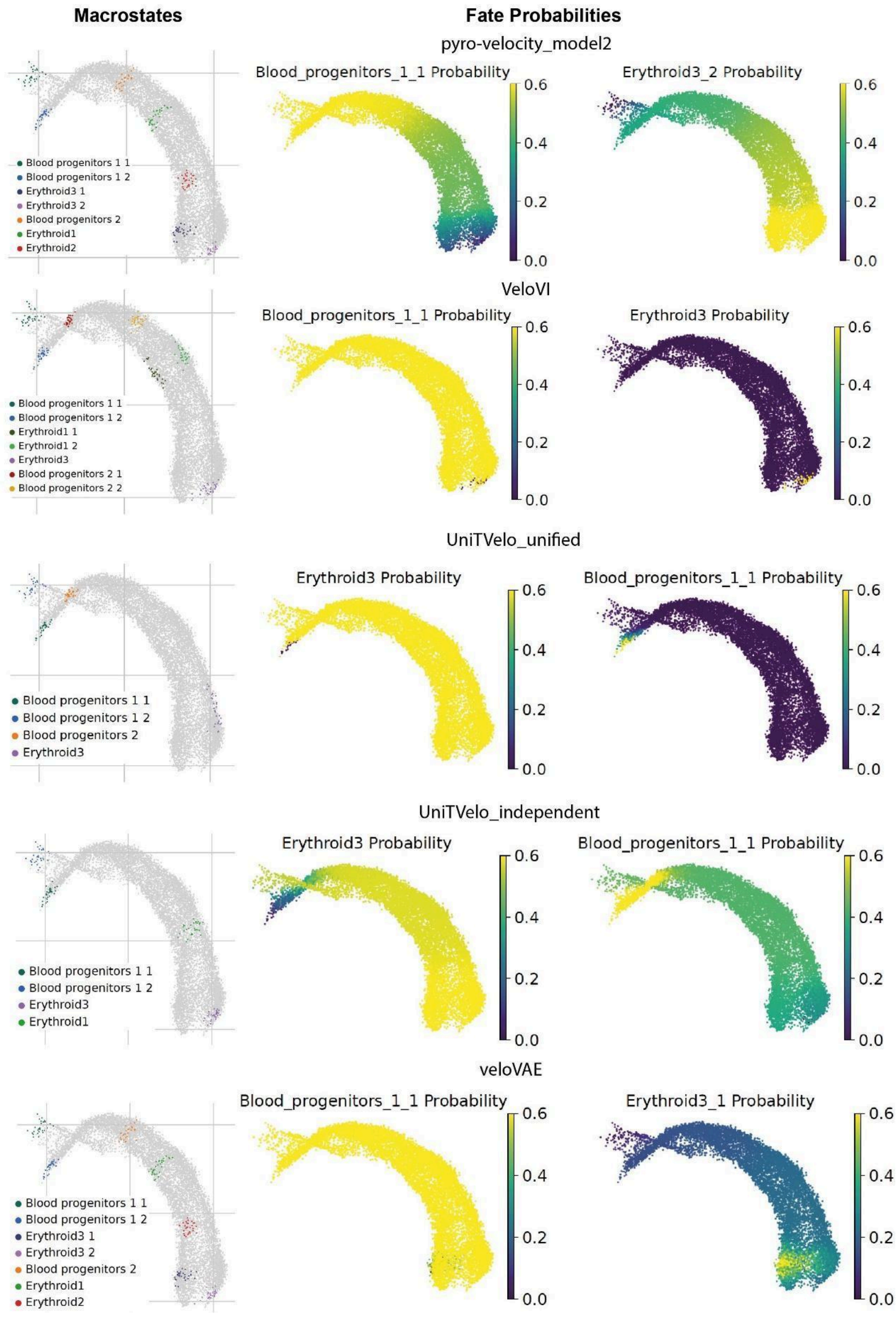


**Supp. Figure 7: CellRank Macrostates (left) and Fate Probabilities (right) for velocity graph inputs from 5 RNA velocity methods on the Erythroid Maturation dataset** *All methods identify macrostates both in progenitors and mature Erythroid cells, but all methods except UniTVelo_unified erroneously assign high fate probabilities to the progenitor state throughout the trajectory.*

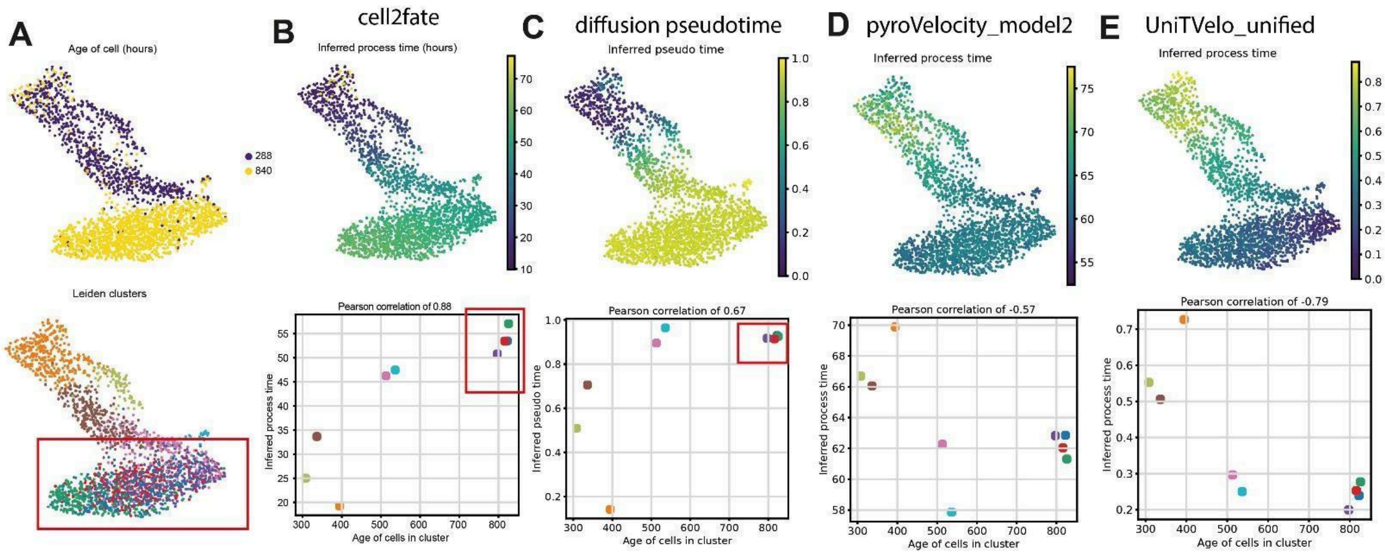


**Supp. Figure 8: Comparison of developmental age of mouse samples to time estimates in the Dentate Gyrus dataset A:** Developmental age of each cell (top) and Leiden clustering (bottom). **B to E:** Top panels show estimated time for each cell on a UMAP, using 4 different methods. The bottom panels show the average developmental age of cells in each Leiden cluster (x-axis) vs. the average estimated time within each Leiden cluster (y-axis). The red rectangle highlights that unlike pseudotime, cell2fate correctly infers an increasing time in mature Granule neurons.

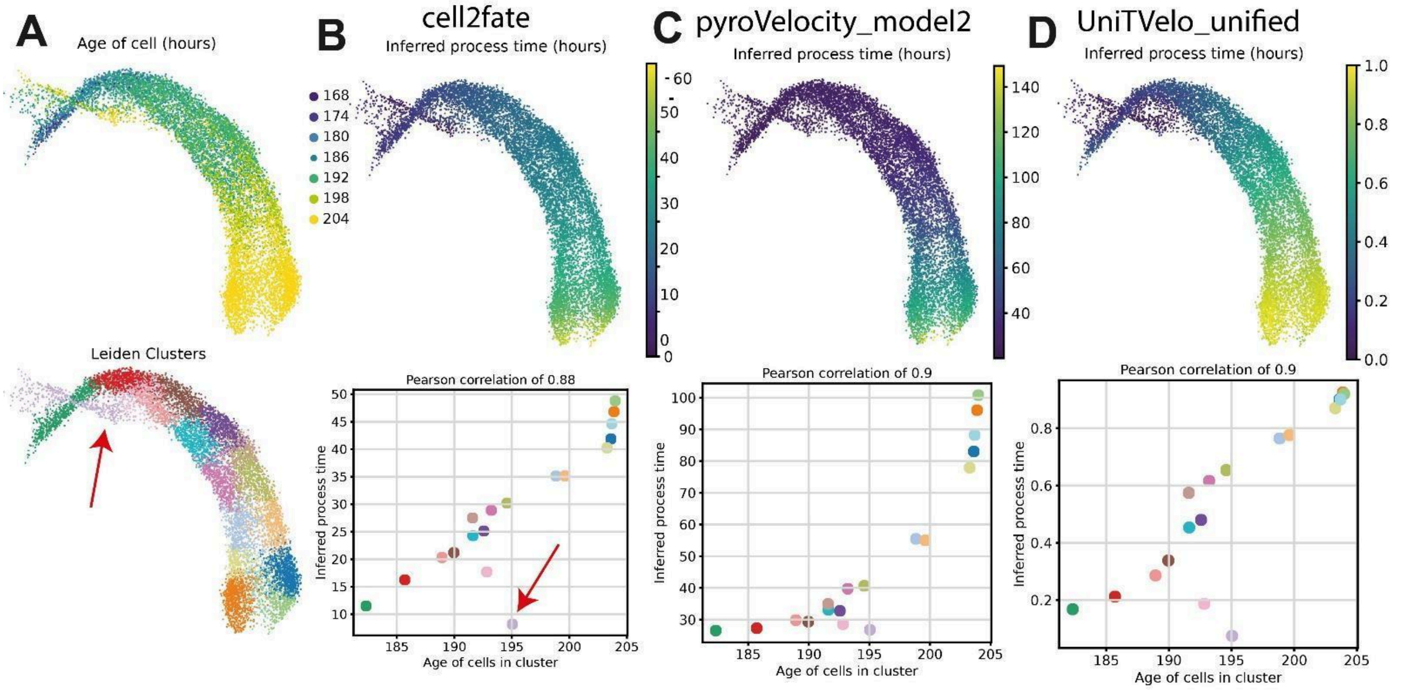


**Supp. Figure 9: Comparison of developmental age of mouse samples to time estimates in the Erythroid Maturation dataset A:** Developmental age of each cell (top) and Leiden clustering (bottom). **B to D:** Top panels show estimated time for each cell on a UMAP, using 4 different methods. The bottom panels show the average developmental age of cells in each Leiden cluster (x-axis) vs. the average estimated time within each Leiden cluster (y-axis).The red arrow highlights a progenitor cluster that belongs to a sample with high developmental time, thus highlighting the limitations of equating developmental time and the time of the inferred differentiation process across all clusters.

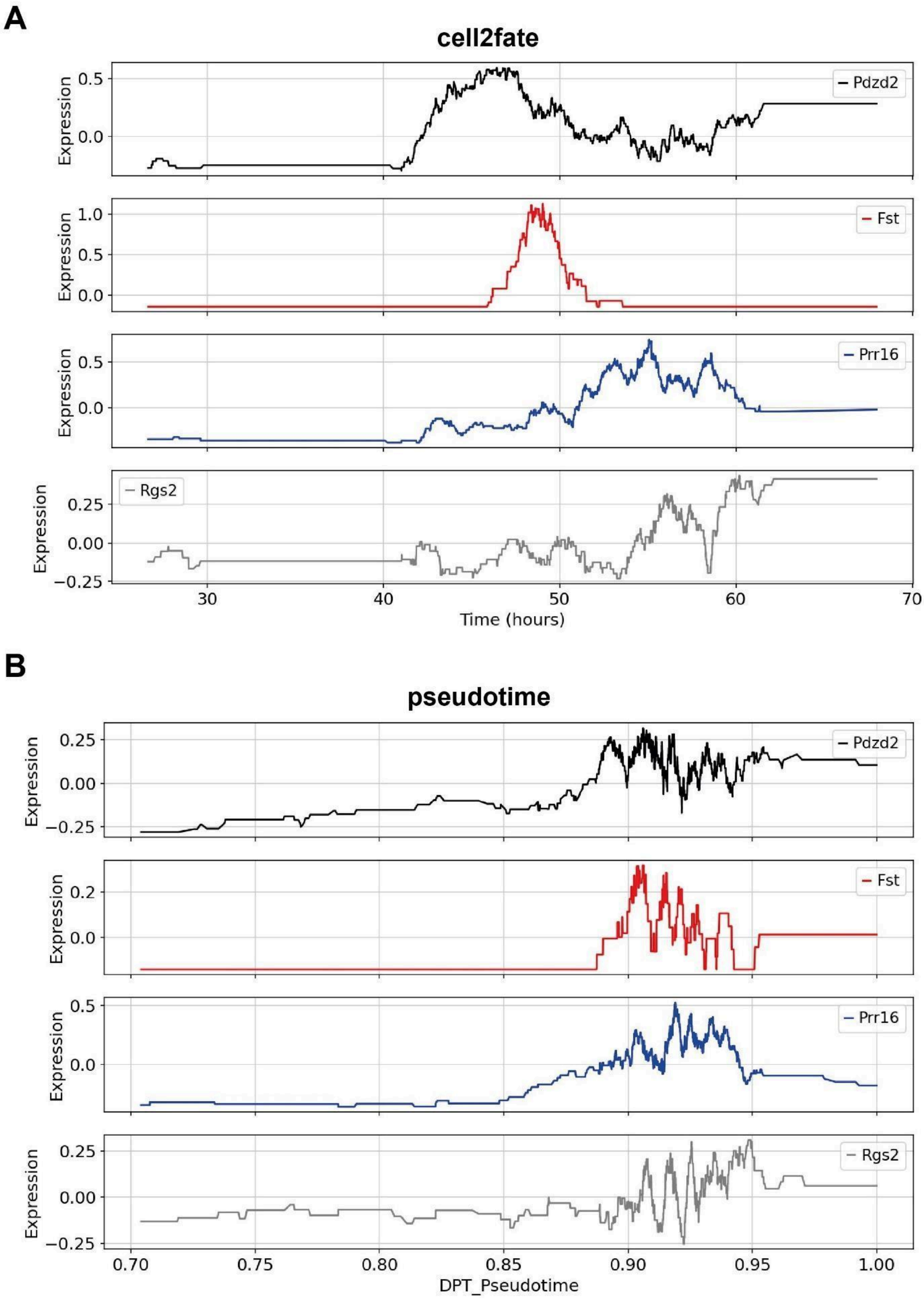


**Supp. Figure 10: Sequential activation of cell2fate module marker genes during final stages of granule neuron maturation ordered by cell2fate time or pseudotime** The expression shown is a moving average of 100 cells of the log2 transformed and total count normalized UMI counts in each cell **A:** Cells ordered by cell2fate time **B:** Cells ordered by pseudotime. Only ordering by cell2fate estimated time reveals the sequential gene expression patterns.

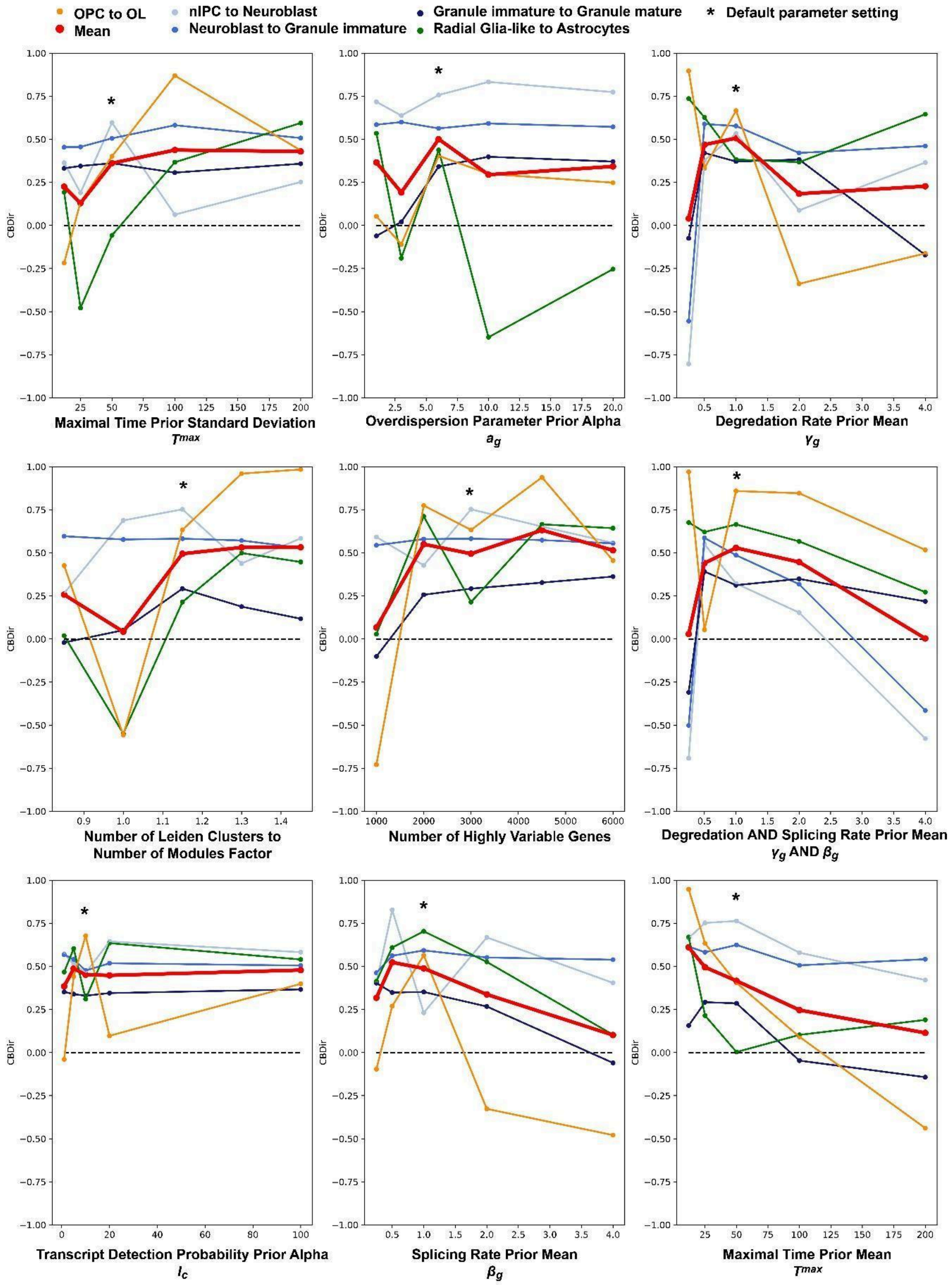


**Supp. Figure 11: Performance (CBDir values) of cell2fate on the Dentate Gyrus dataset with various preprocessing choices and prior distribution settings.** *The x-axis label shows the name of the prior distribution that was varied in the first row and the symbol of the parameter for which the prior distribution was varied in the second row. In addition, the number of modules to the number of Leiden clusters factor was varied and the number of input genes (row 2, left and middle). The star indicates the default parameter setting. Overall performance is robust, with the CBDir metric staying positive on average. However, within lowly abundant populations, such as OPCs, performance can be sensitive to strong deviations in prior parameter settings. Interestingly, performance may be increased by setting the prior distribution on the maximal time (Tmax) in the dataset to a lower mean, but a higher standard deviation.*

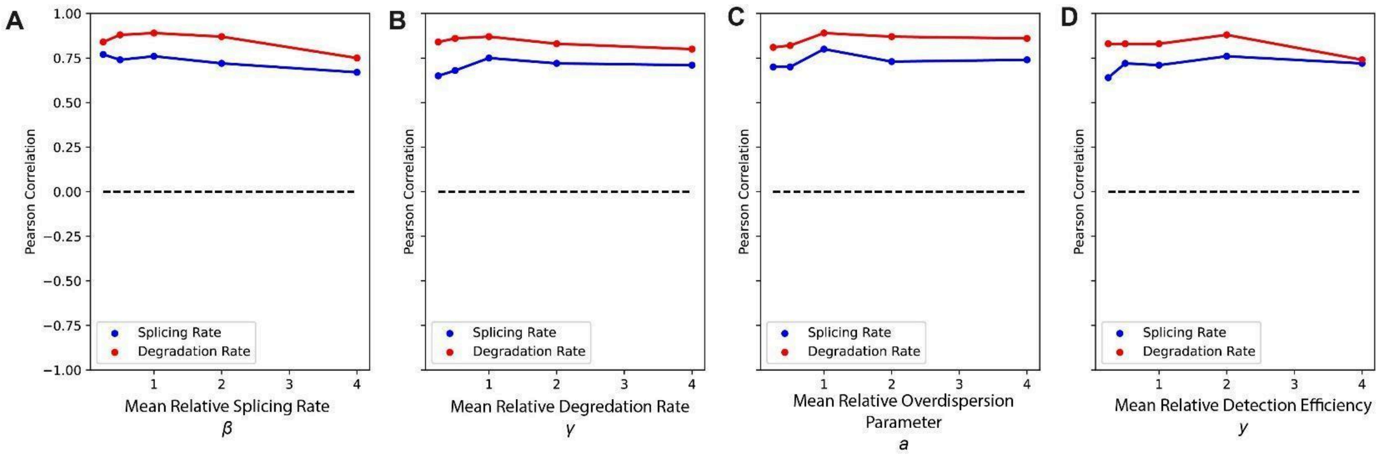


**Supp. Figure 12: Correlation of cell2fate inferred splicing and degradation rates compared to ground truth simulated from the cell2fate generative model with different parameter choices.** *Inferred parameter values on the Dentate Gyrus dataset were taken as starting values for the simulation and then four chosen parameters were increased or decreased by a common factor across genes or cells to produce multiple simulated datasets. Results on simulated data are shown with A: varying splicing rates across genes B varying degradation rates across genes C varying overdispersion parameter (noise) across genes D varying detection efficiency across cells. Overall, low correlations were obtained when the simulated splicing rate became much larger than the degradation rate, when the noise in the data was increased (i.e. a low overdispersion parameter) and when the detection efficiency of transcripts was lowered.*

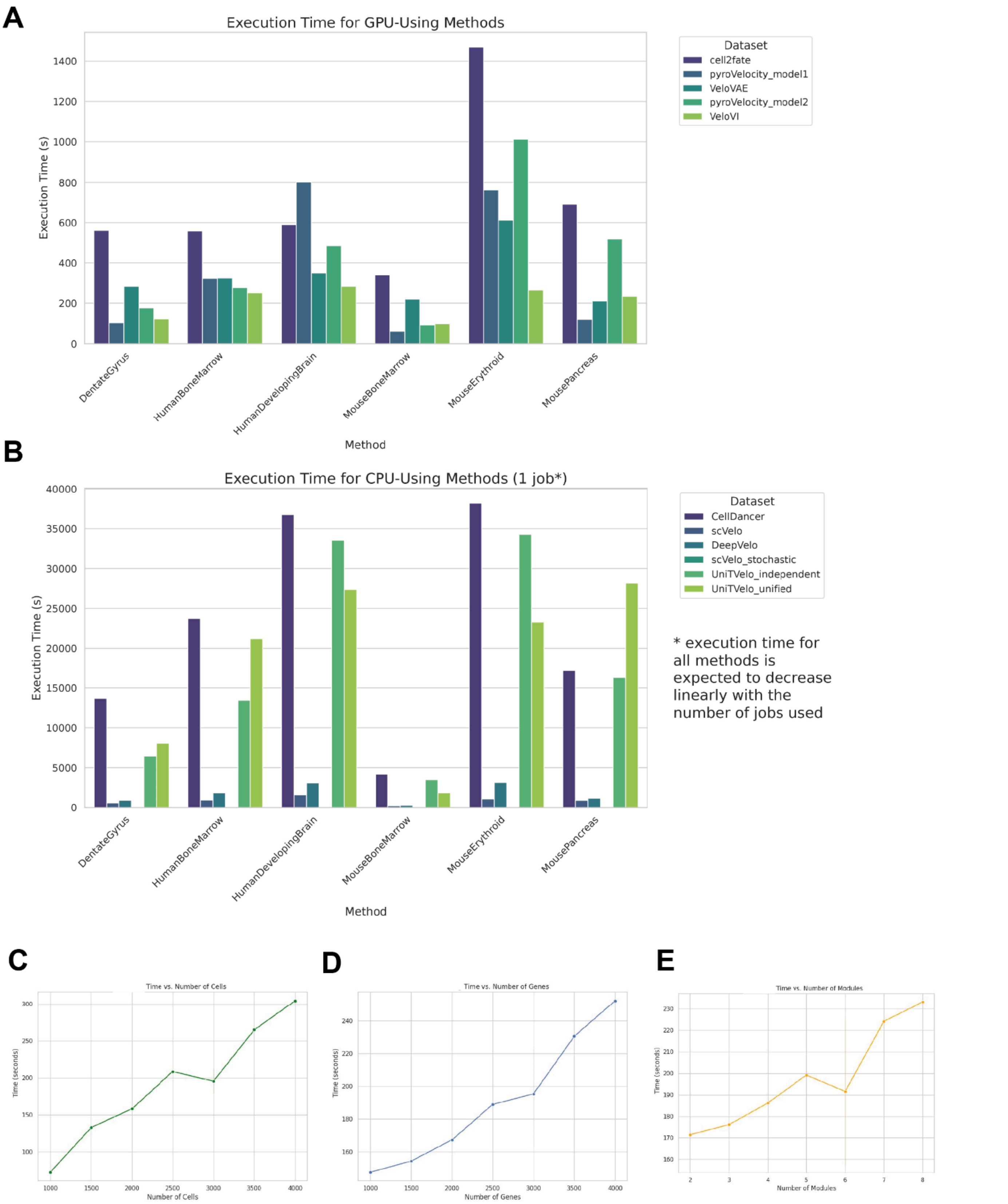


**Supp. Figure 13: Execution time of methods on different datasets A:** Execution times of gpu-based methods on all datasets **B:** Execution times of cpu-based methods on all datasets using 1 job. Execution times can be expected to decrease linearly with the number of jobs used. **C:** Execution time for cell2fate as a function of cell numbers randomly sampled from the Erythroid maturation dataset fixed at 3000 genes and 6 modules. **D:** Execution time for cell2fate as a function of highly variable genes using the Erythroid maturation dataset fixed at 3000 randomly sampled cells and 6 modules. **E:** Execution time for cell2fate as a function of number of modules using the Erythroid maturation dataset fixed at 3000 genes and 3000 cells.

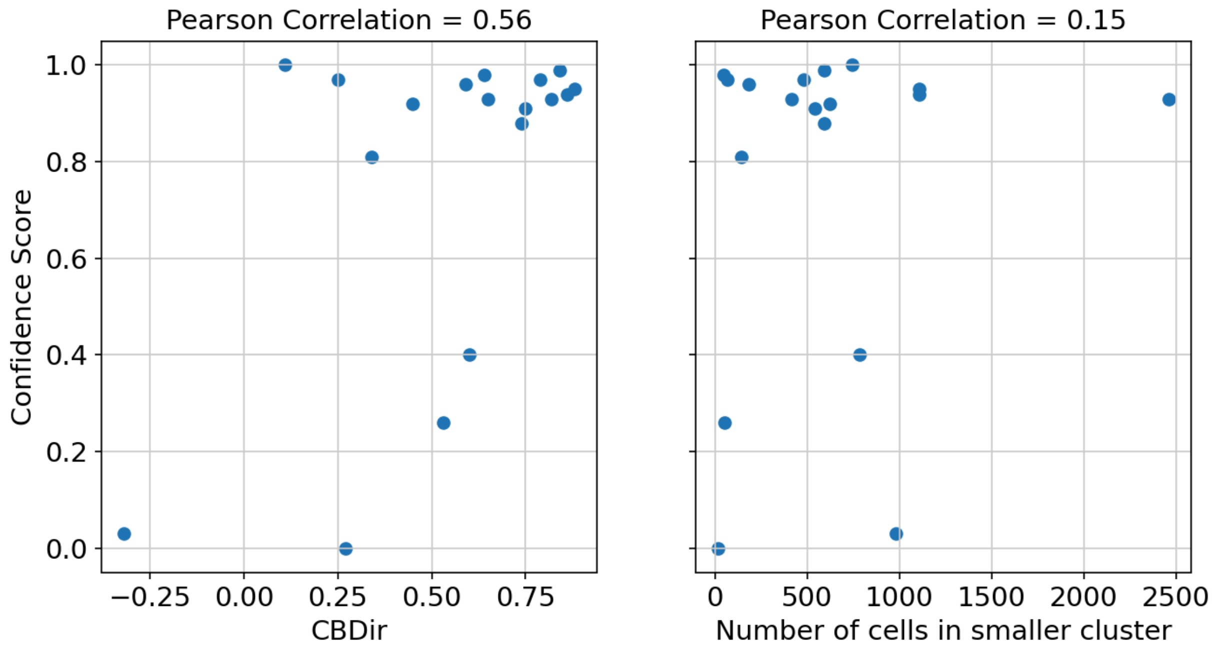


**Supp. Figure 14: Cell2fate confidence score for each transition on the y-axis vs. CBDir metric (left) or the number of cells (of the smaller of two clusters) in the transitions (right)** The moderately strong dependence on the CBDir metric suggests that this score can highlight potentially wrongly inferred transition directions.

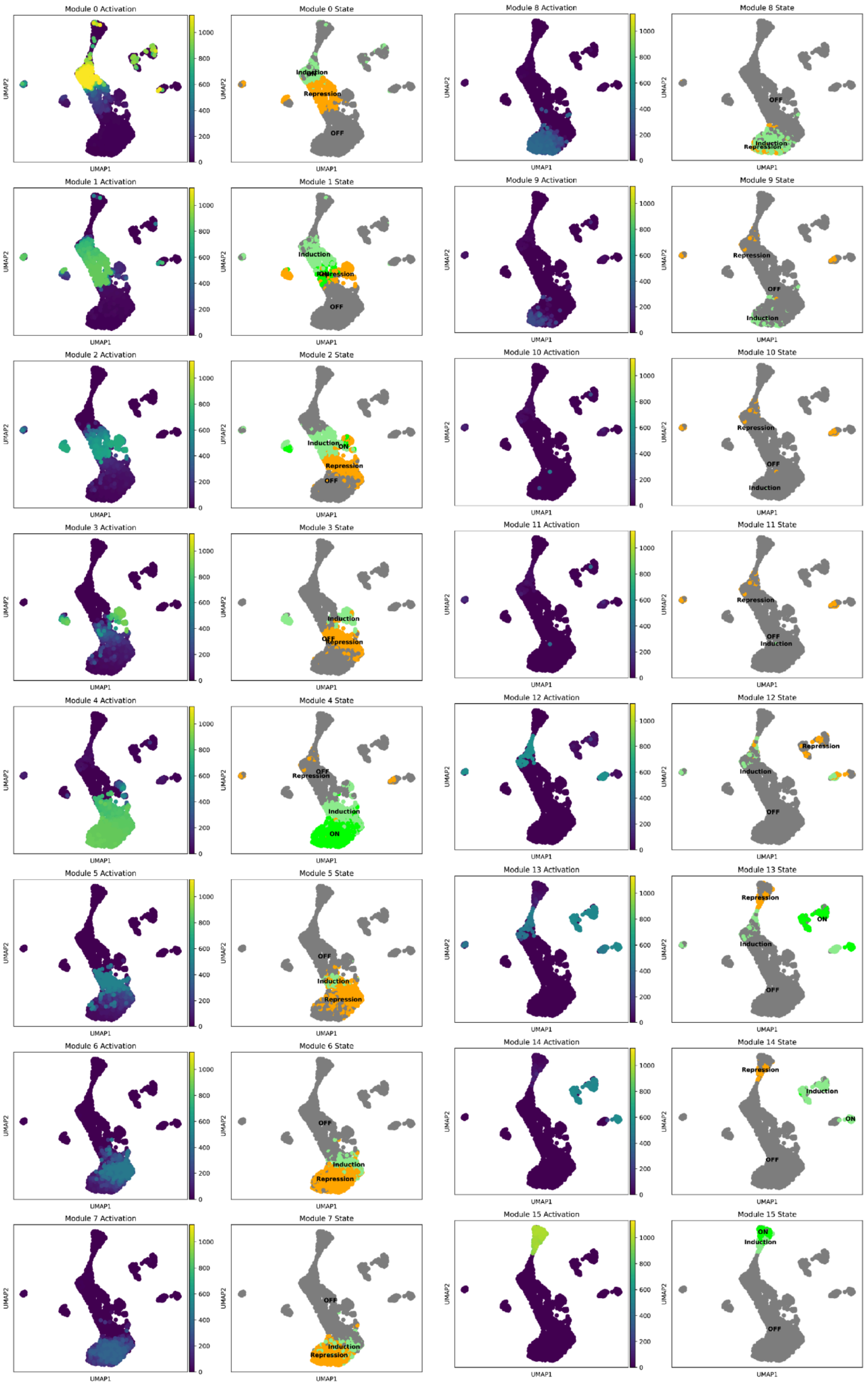


**Supp. Figure 15: module activation and state UMAP plots for mouse dentate gyrus data**

*UMAPs coloured by module activation in columns 1 and 3 and state in columns 2 and 4, highlight a sequence of transcriptional programs switched on during differentiation.*

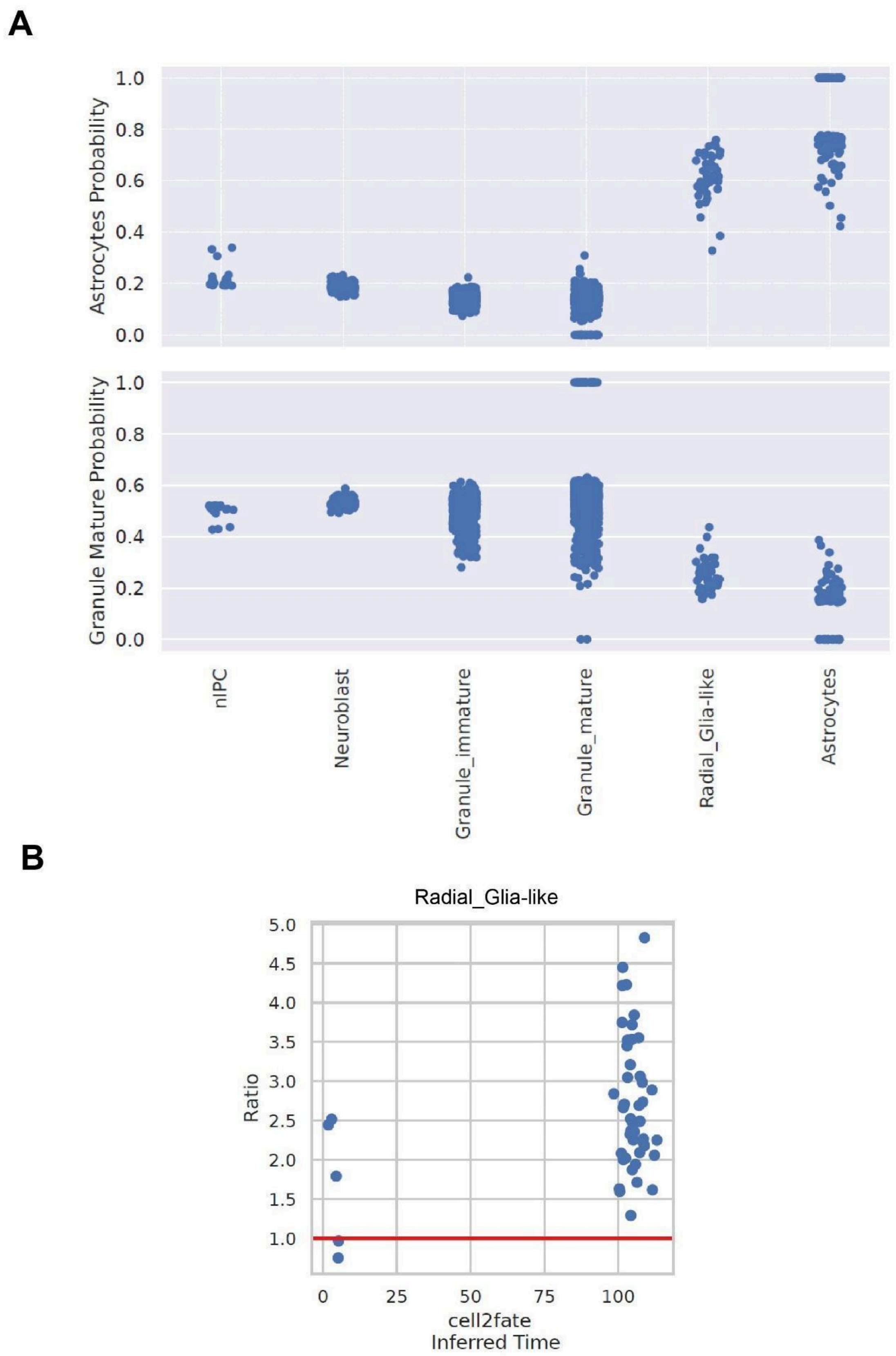


**Supp. Figure 16: Comparison of cell2fate time estimates and cellRank ratio of Astrocyte to Granule Neuron fate probabilities for Radial Glia cells A:** Astrocyte and Granule neuron fate probabilities **B:** *Cells with high probability for the Astrocyte fate tend to have a higher inferred time by cell2fate. Overall, almost all Radial Glia cells are committed to Astrocytes.*

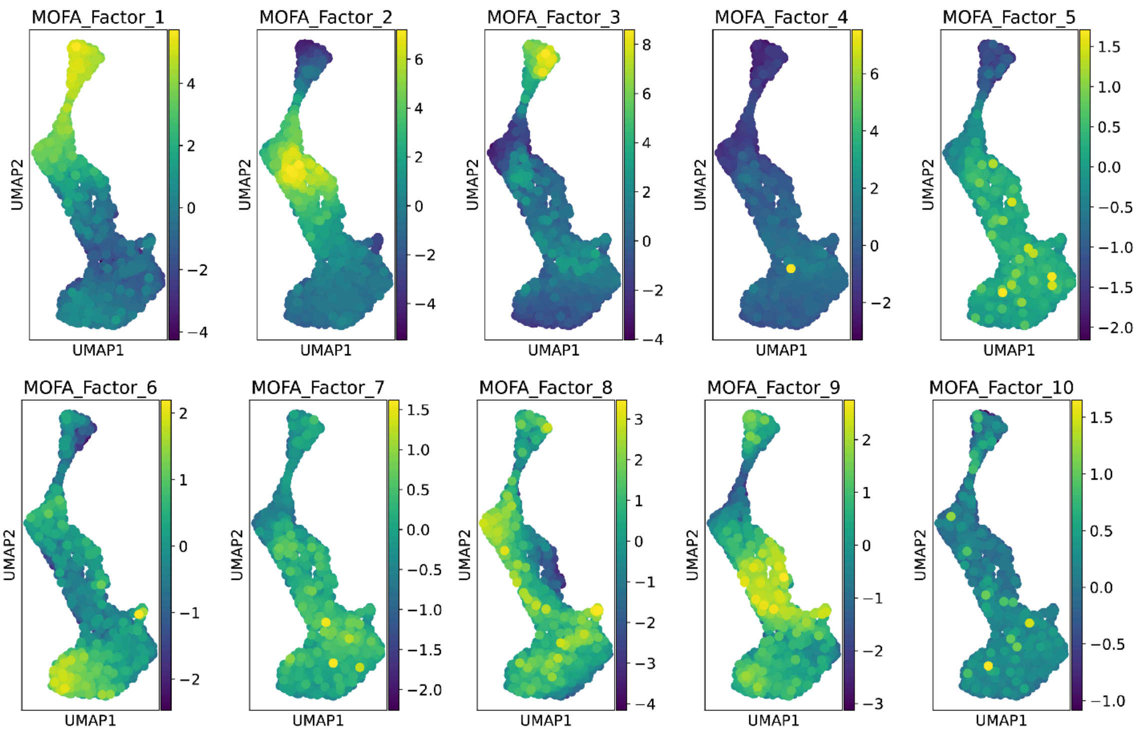


**Supp. Figure 17: Dentate Gyrus data factors obtained with MOFA factors** *Some MOFA factors associate with the differentiation trajectory in this dataset, while others are more broadly distributed.*

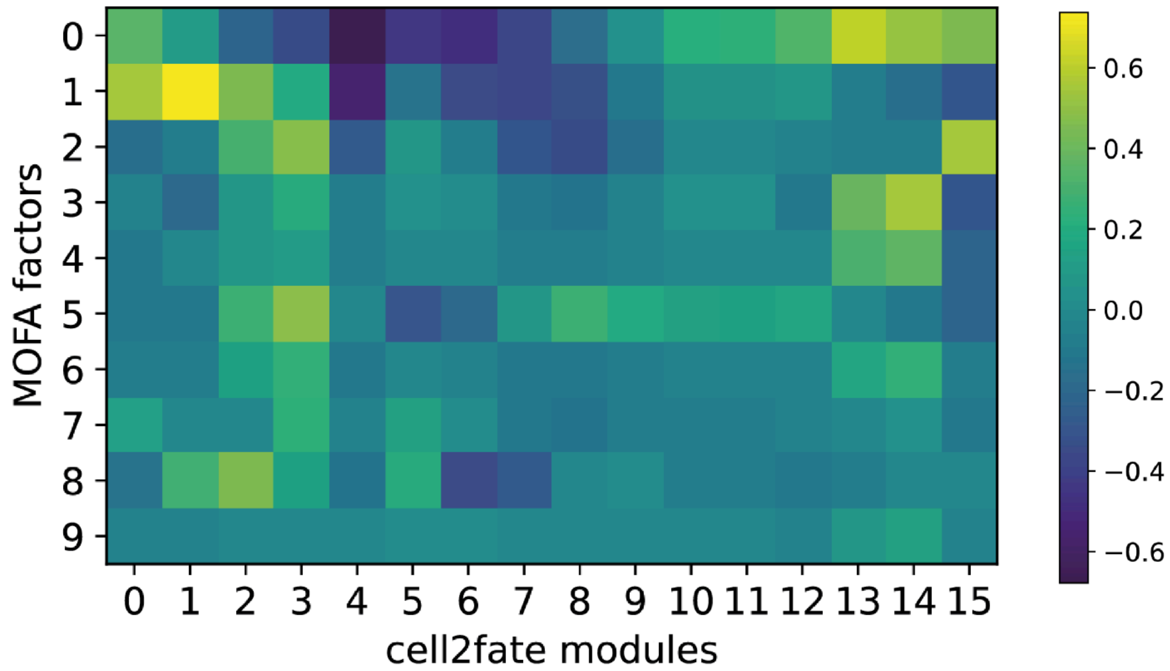


**Supp. Figure 18: Correlations between gene loadings of MOFA factors and cell2fate modules** *Correlations are low throughout, with particularly low values for modules 4 to 9 that are active in neurons. This indicates that cell2fate and MOFA find largely distinct expression dimensions.*

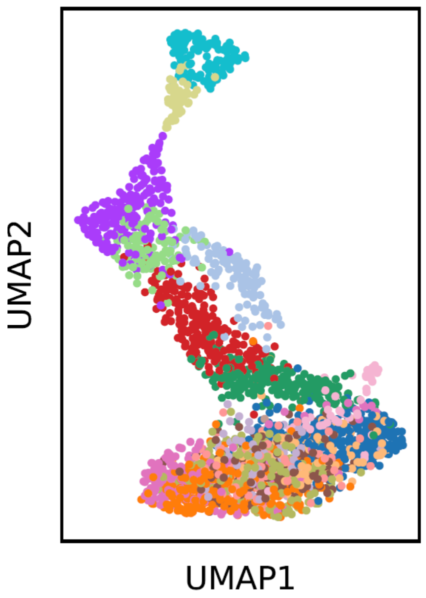


**Supp. Figure 19: Leiden clustering of dentate gyrus data with resolution parameter set to 2** *Leiden clusters partition the differentiation trajectory of Astrocytes (top of UMAP) neuronal progenitors and neurons (middle), but are diffusely distributed across mature granule neurons (bottom).*

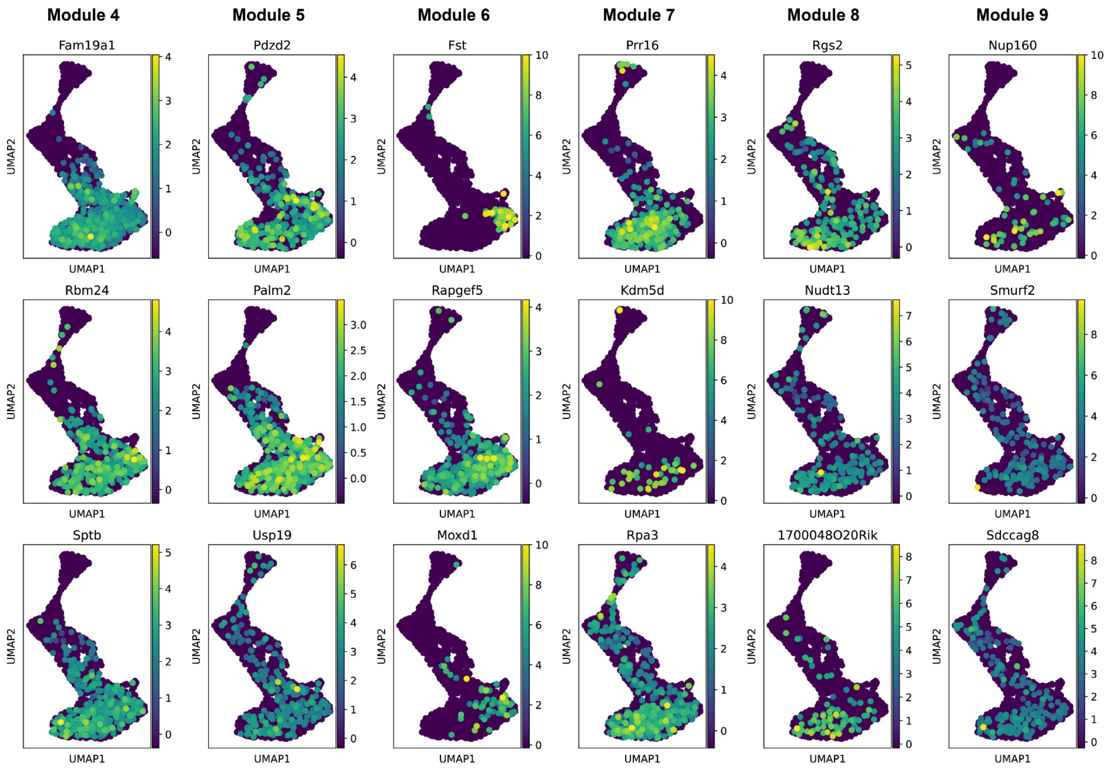


**Supp. Figure 20: Module marker genes in Dentate Gyrus data** *Modules are ordered from left to right, the most significant module gene is at the top, followed by the second and third.*

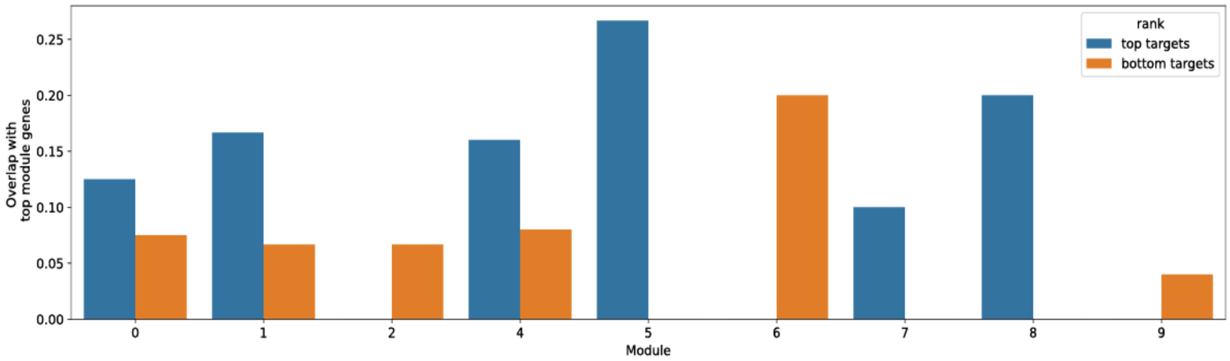


**Supp. Figure 21: overlap of top 10 and bottom 10 binding targets of top 20 module transcription factors with top 300 module genes.** *Putative promoter sequences were first extracted in the genomic vicinity of the top 300 module genes of each module. Binding affinities were then predicted between these sequences and top 20 module transcription factors using the ProBound*[25] *algorithm.*

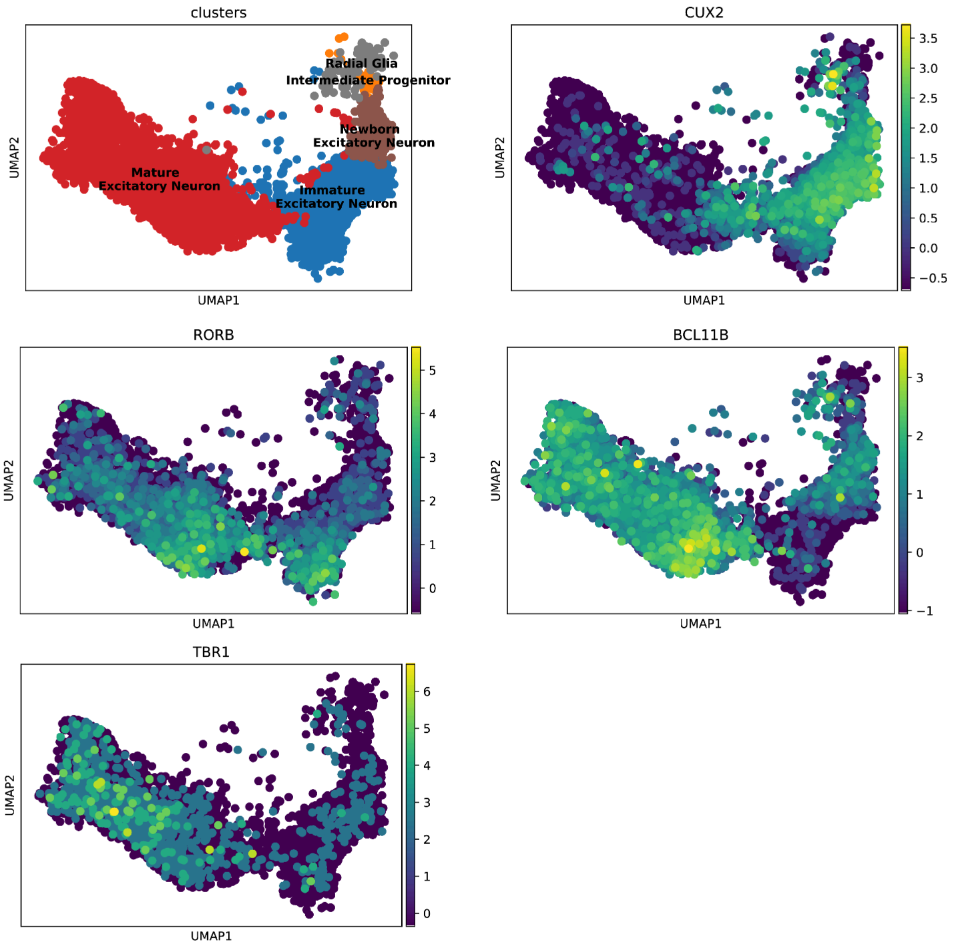


**Supp. Figure 22: neuronal subtype markers visualised in human developing brain data UMAP**

*Correspondence between subtypes and markers is as follows: CUX2 = upper layer, RORB = layer 4, BCL11B = layer 5, TBR1 = layer 6*

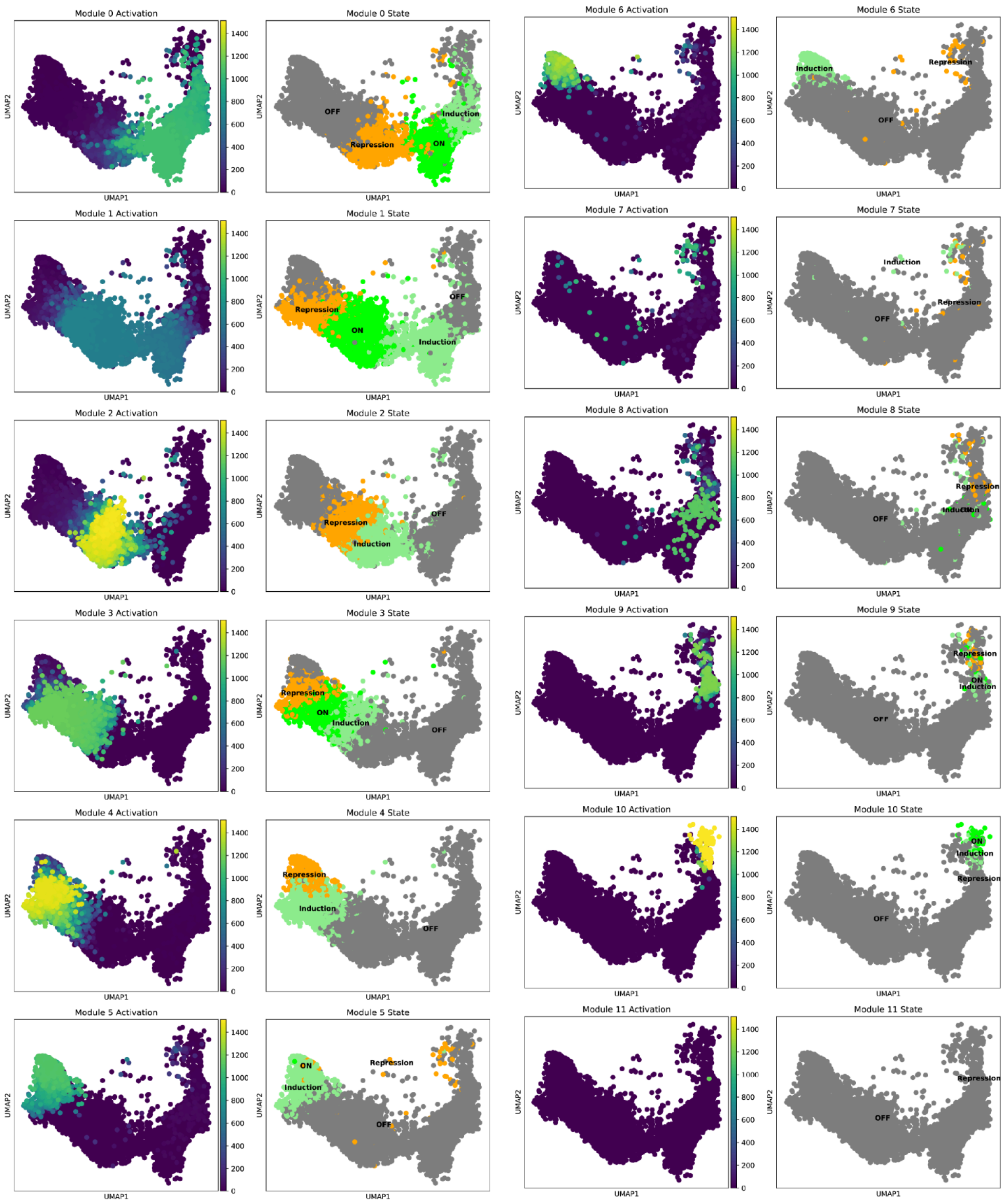


**Supp. Figure 23: module activation and state UMAP plots for human brain data**

*UMAPs coloured by module activation in columns 1 and 3 and state in columns 2 and 4, highlight a sequence of transcriptional programs switched on during differentiation.*

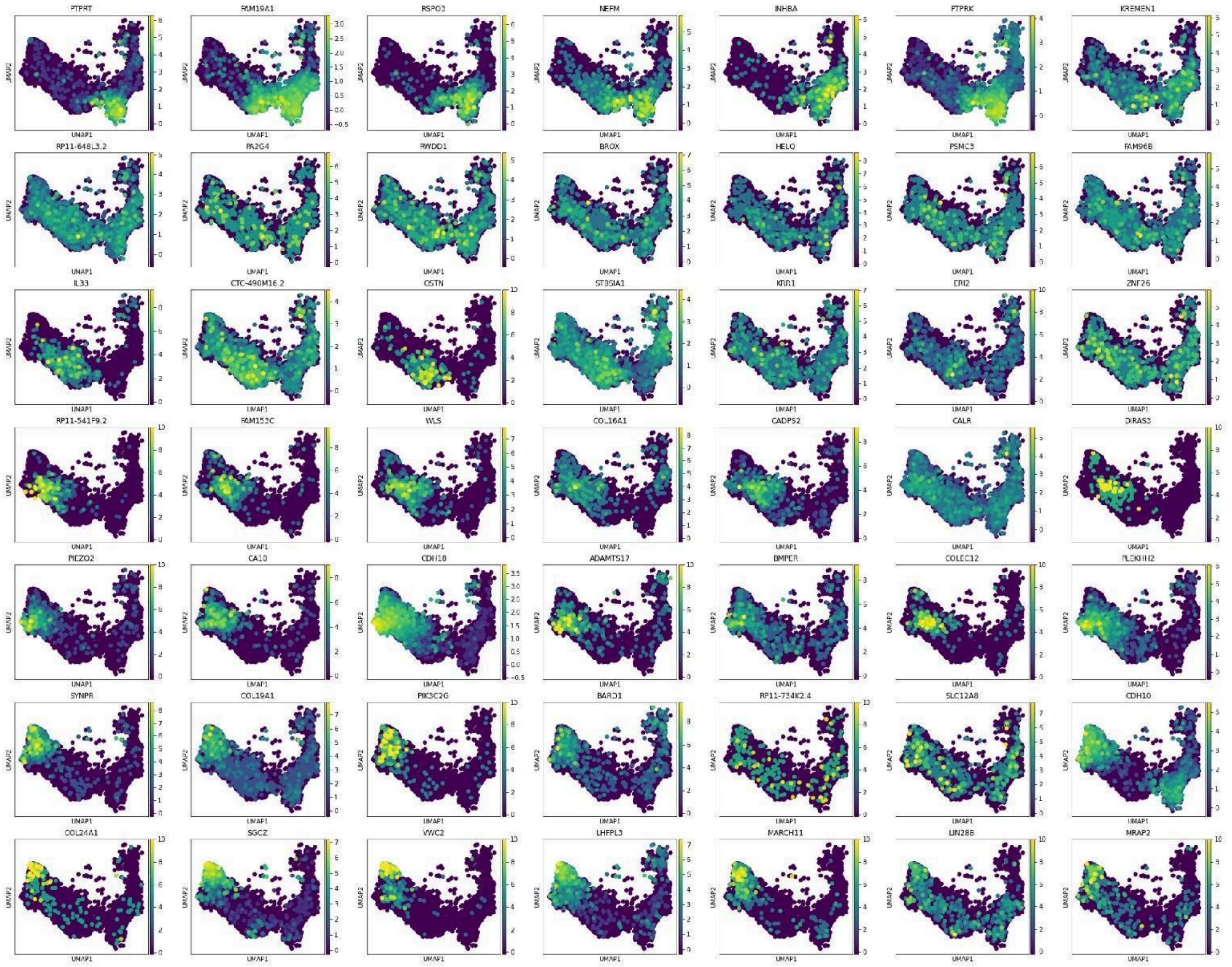


**Supp. Figure 24: top 7 marker genes (left to right) for modules 0 (top) to 6 (bottom) in human developing brain data**

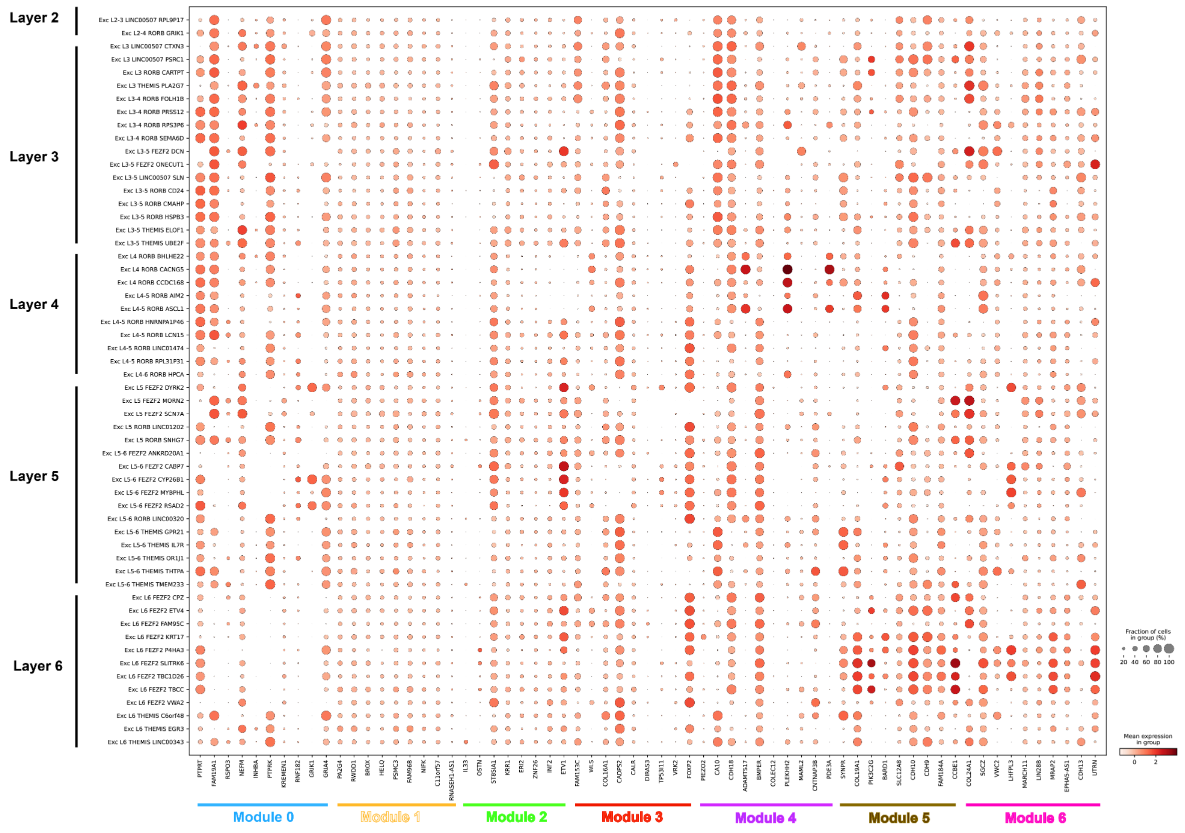


**Supp. Figure 25: Expression of top 10 markers of all 7 neuron differentiation modules in adult human brain data**

*Cell2fate module markers (left to right) are mostly expressed across all neuronal subtypes (top to bottom), indicating that cell2fate modules mostly capture a pan-neuronal differentiation trajectory.*

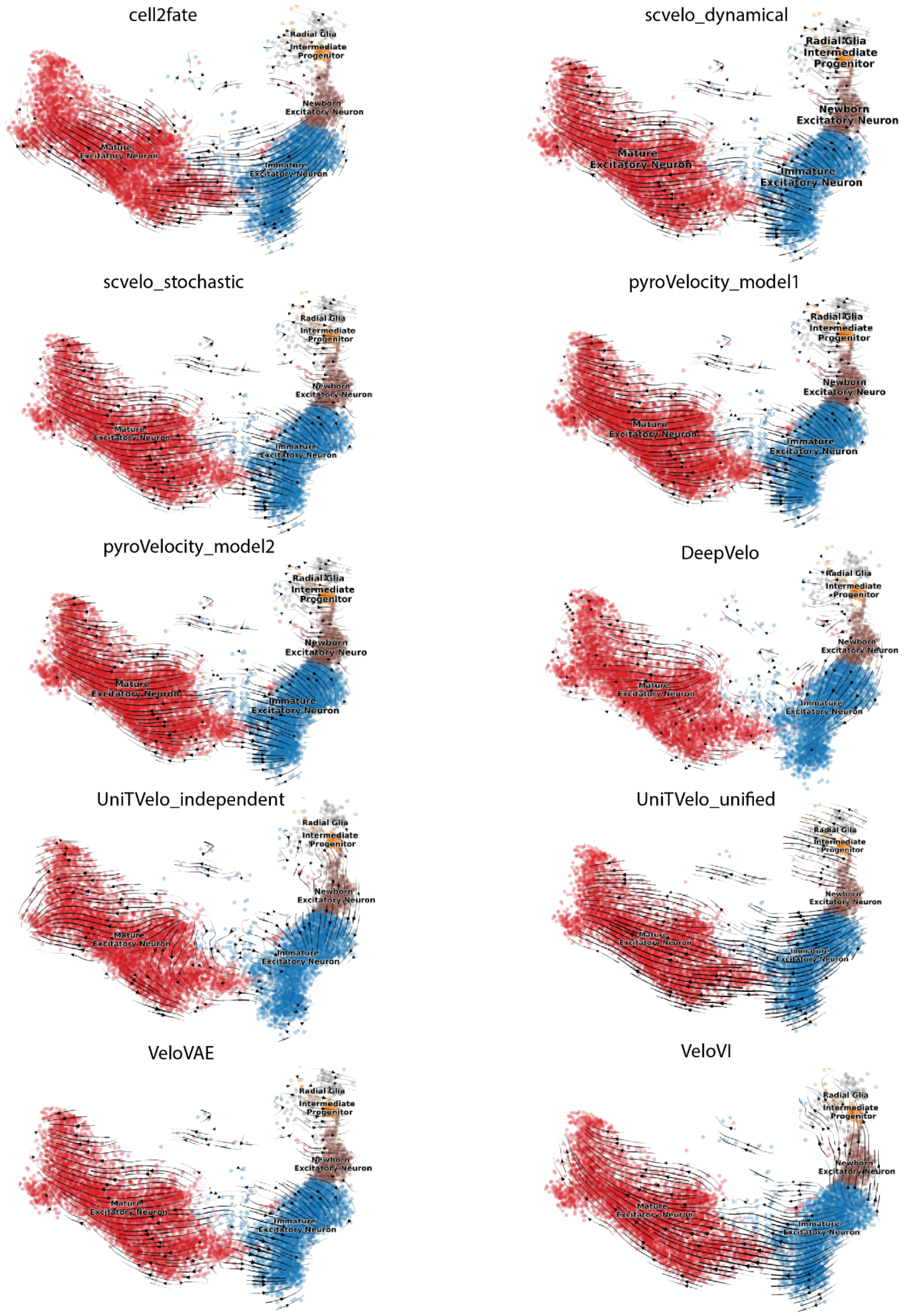


**Supp. Figure 26: Velocity Graph UMAP projections for all 10 RNA velocity methods for human developing brain data**

*cell2fate accurately captures the differentiation trajectory from progenitors (right) to mature neurons (left).*

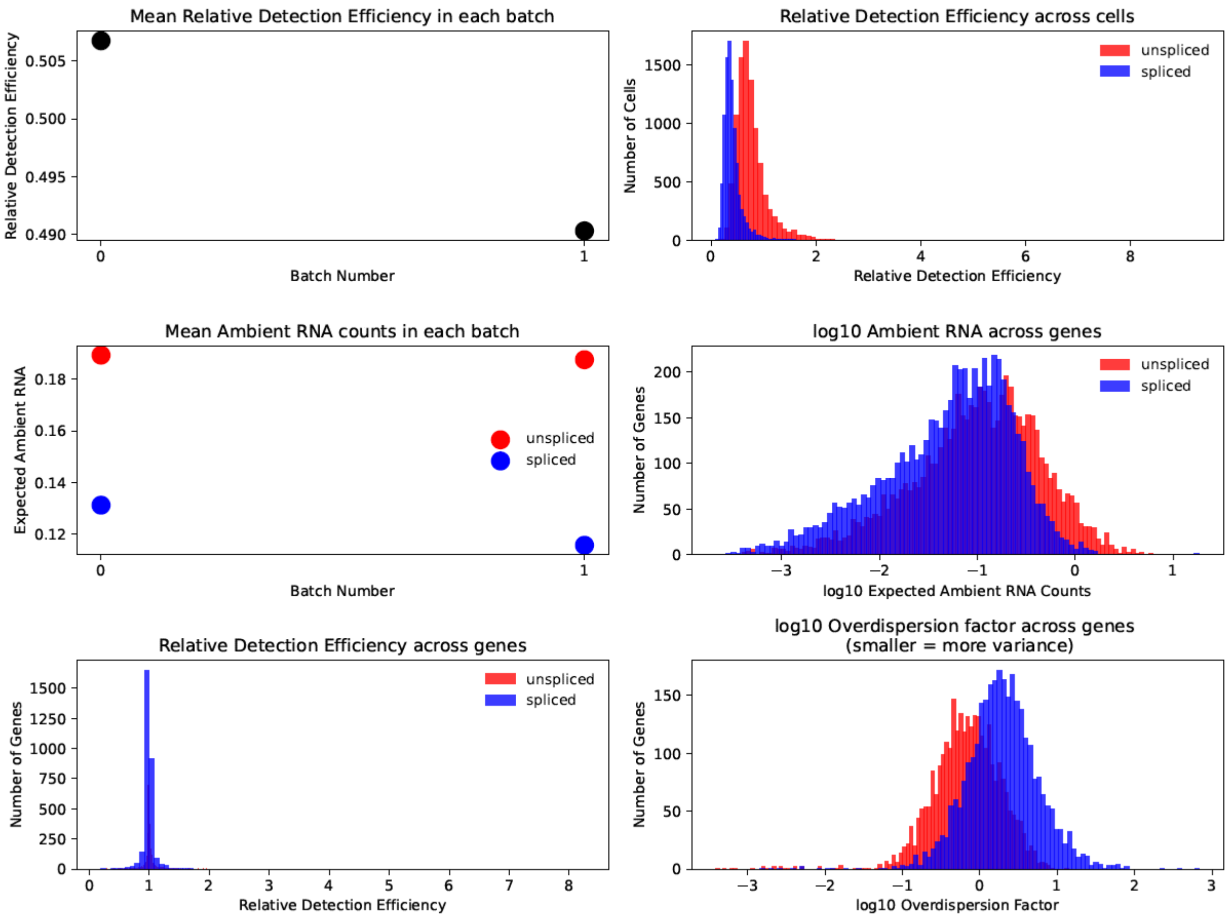


**Supp. Figure 27: overview of noise and technical variables for human brain data**

*In contrast to cell2fate model fits on single-cell data, unspliced counts are predicted to have higher detection efficiency than spliced counts in this single-nucleus dataset (top right). However, they still have higher ambient RNA (middle right) and more noise, corresponding to a lower overdispersion parameter (bottom right).*

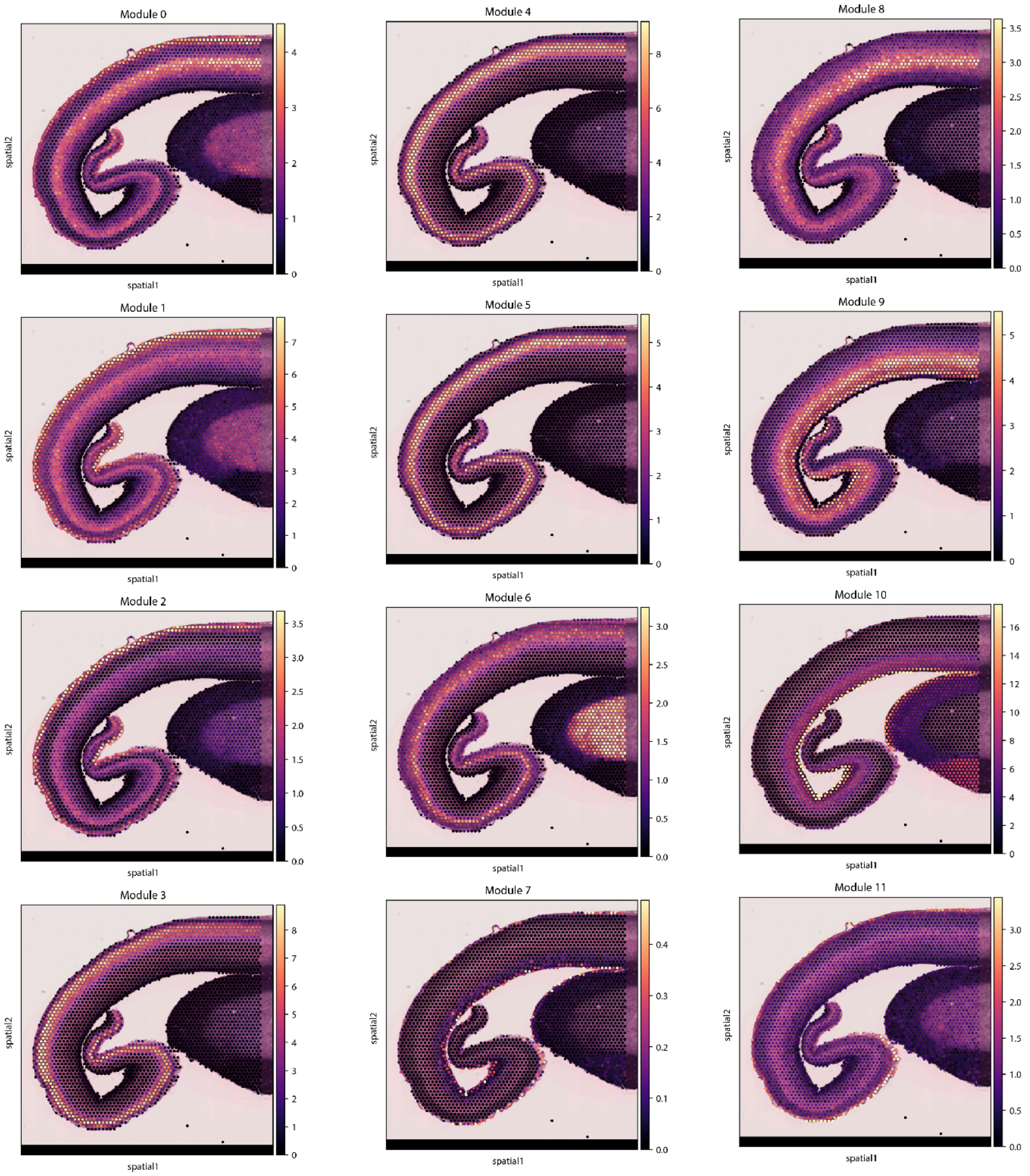


**Supp. Figure 28: cell2location mapping for all cell2fate modules in human developing brain data**

*Cell2fate modules are mapped to distinct locations. Immature neuron modules (e.g. 0,1,2) map towards the inside of the developing human brain section or into the upper layers, while modules corresponding to more mature neurons (e.g. 4,5) map to deep layers.*

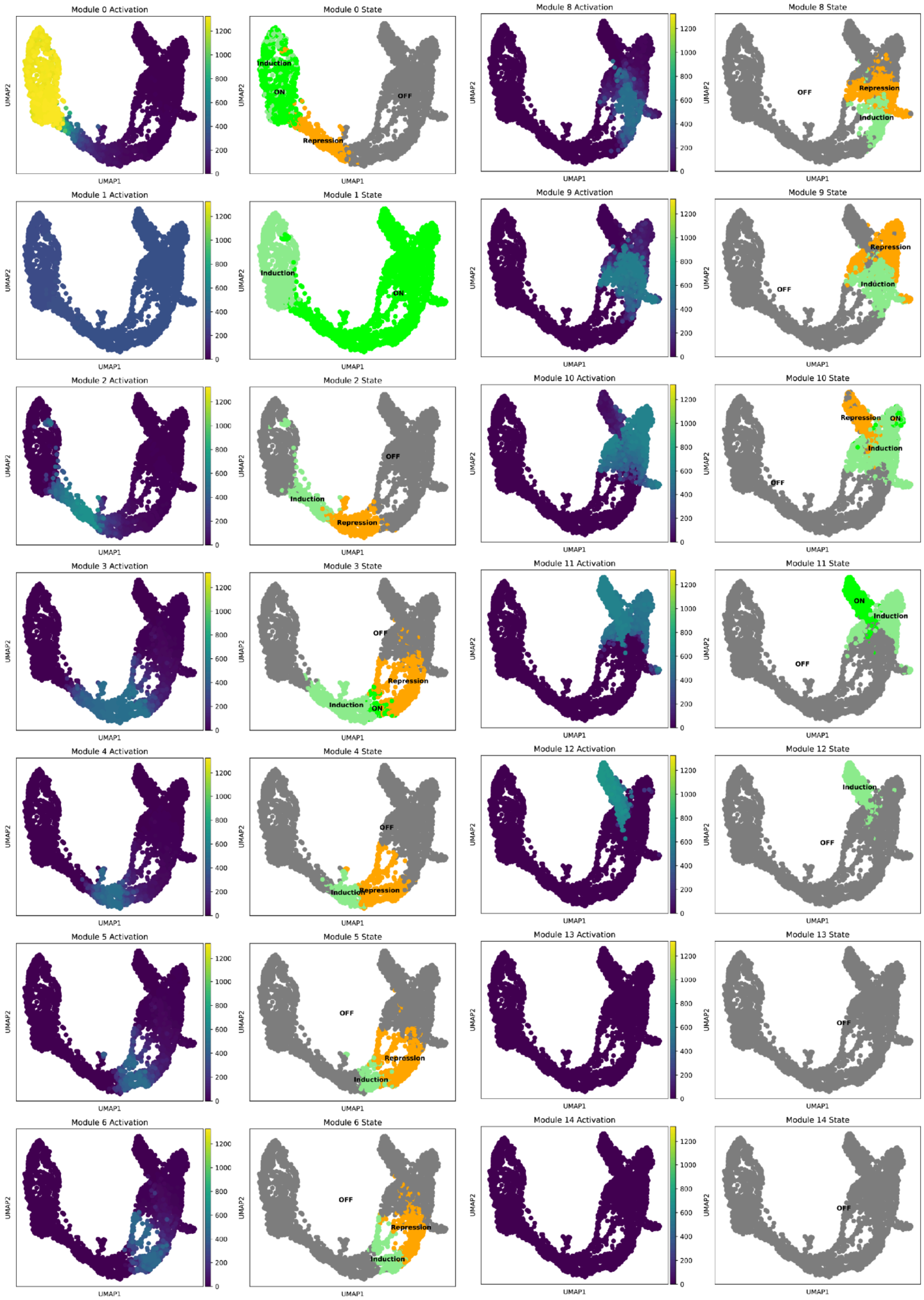


**Supp. Figure 29: module activation and state UMAP plots for mouse pancreas data** *UMAPs coloured by module activation in columns 1 and 3 and state in columns 2 and 4, highlight a sequence of transcriptional programs switched on during differentiation.*

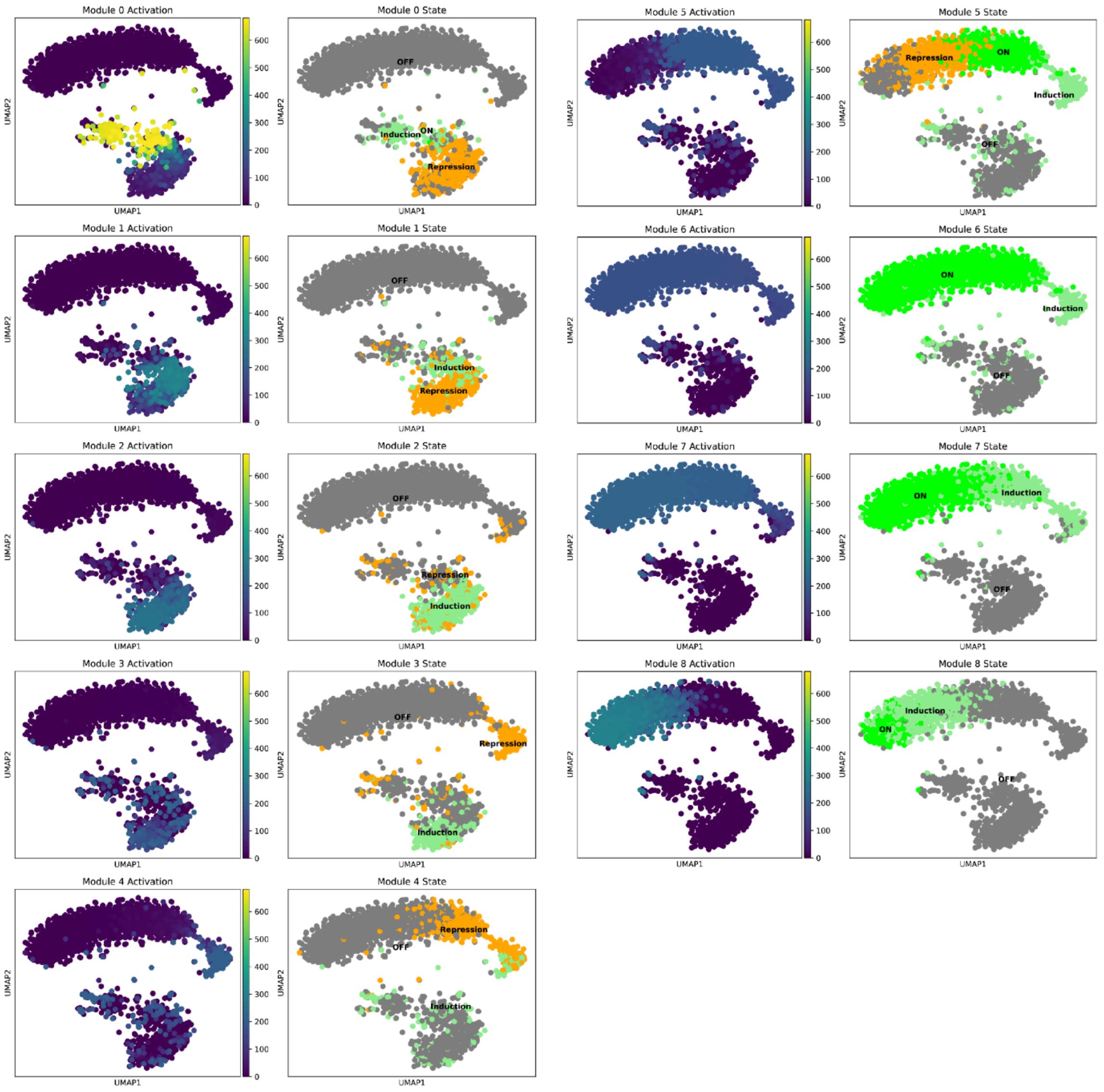


**Supp. Figure 30: module activation and state UMAP plots for mouse bone marrow data**

*UMAPs coloured by module activation in columns 1 and 3 and state in columns 2 and 4, highlight a sequence of transcriptional programs switched on during differentiation.*

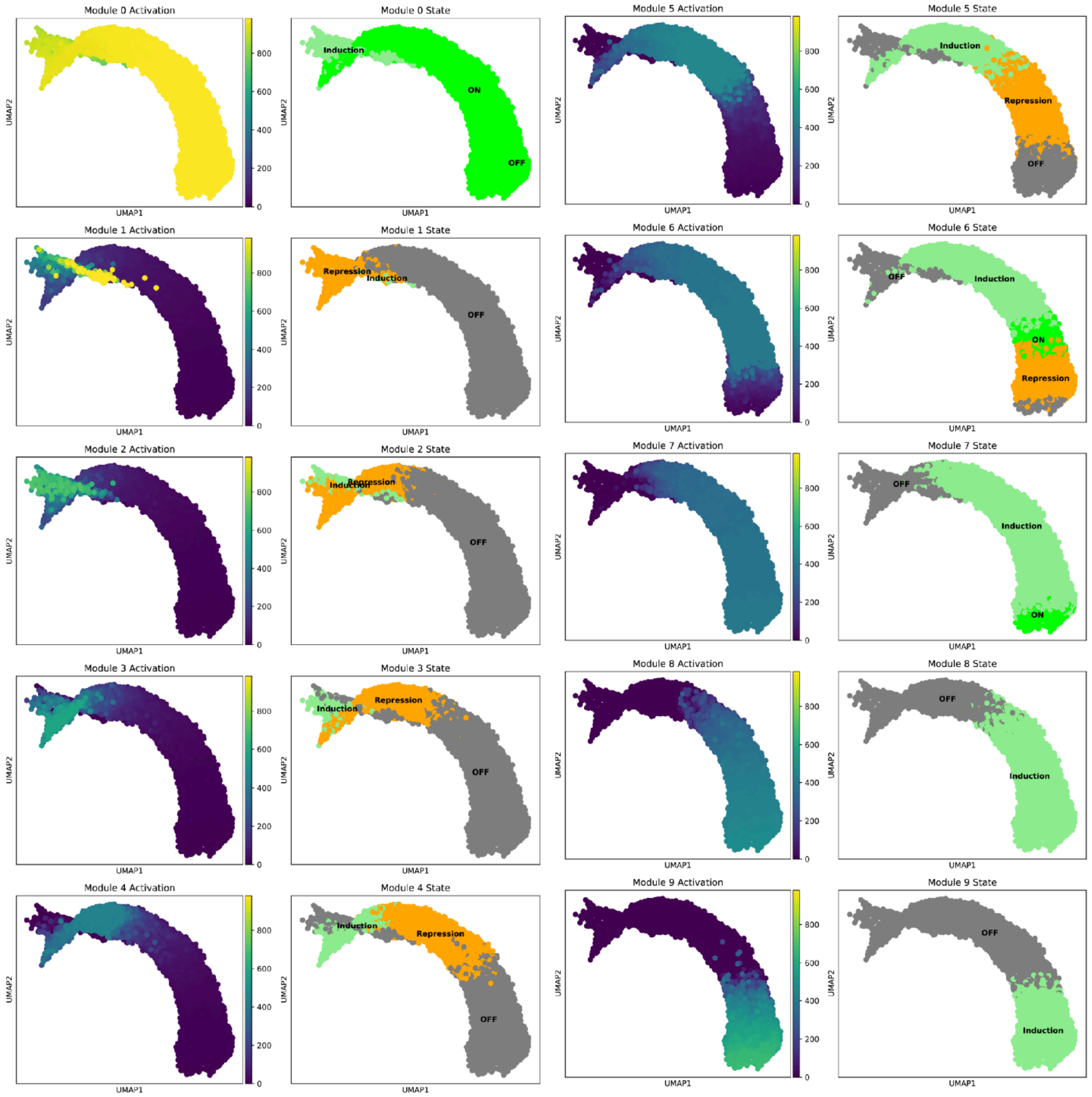


**Supp. Figure 31: module activation and state UMAP plots for mouse erythroid maturation**

*UMAPs coloured by module activation in columns 1 and 3 and state in columns 2 and 4, highlight a sequence of transcriptional programs switched on during differentiation.*

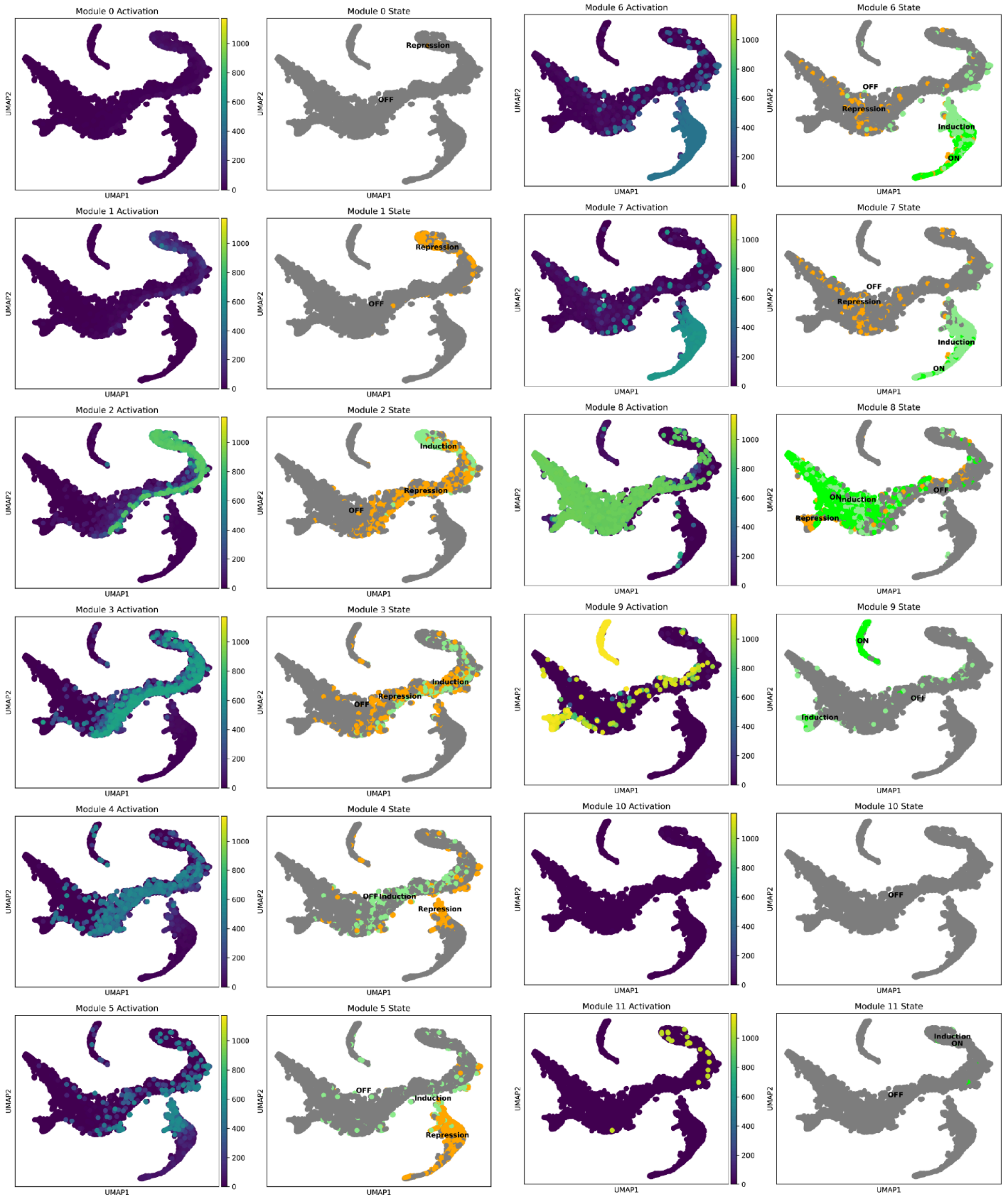


**Supp. Figure 32 : module activation and state UMAP plots for human bone marrow data**

*UMAPs coloured by module activation in columns 1 and 3 and state in columns 2 and 4, highlight a sequence of transcriptional programs switched on during differentiation.*

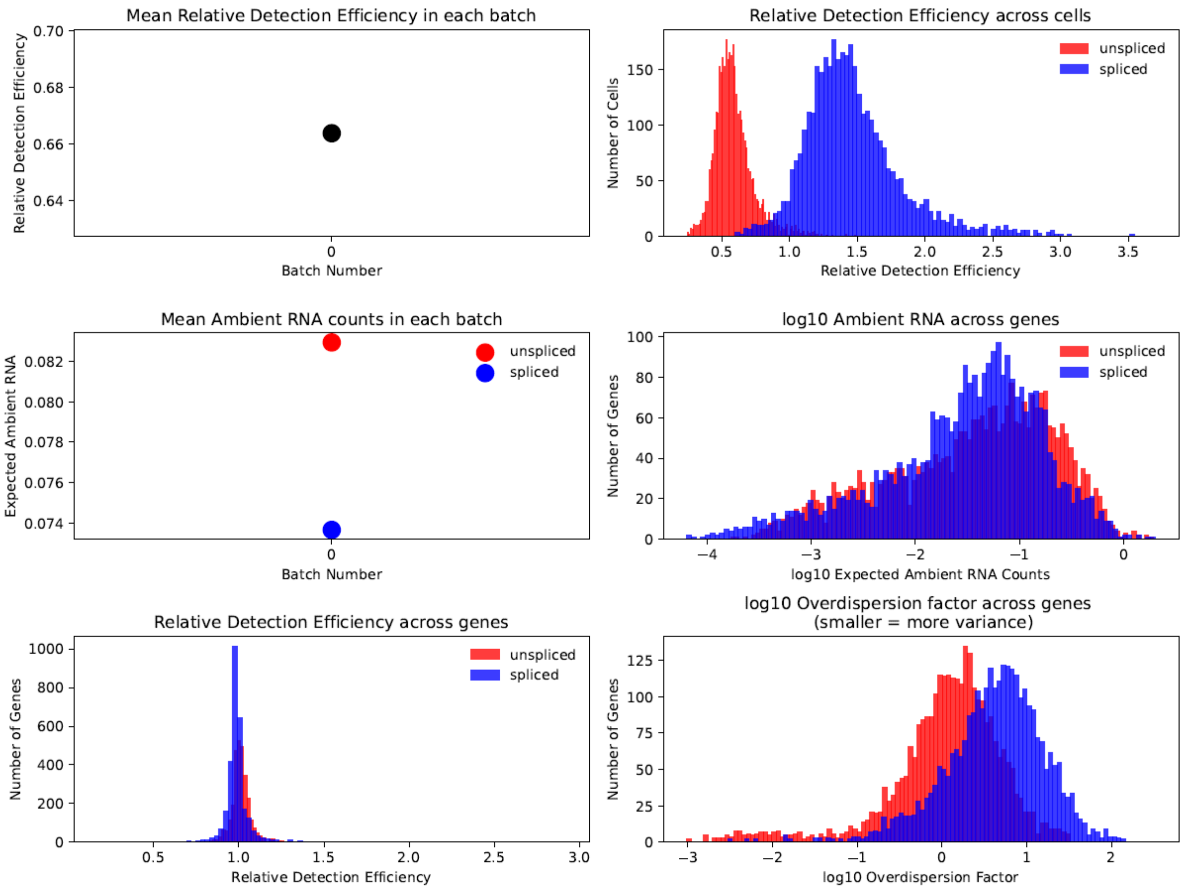


**Supp. Figure 33: overview of noise and technical variables for mouse pancreas data**

*Unspliced counts have lower detection efficiency (top right), higher ambient RNA (middle right) and more noise than spliced counts, corresponding to a lower overdispersion parameter (lower right).*

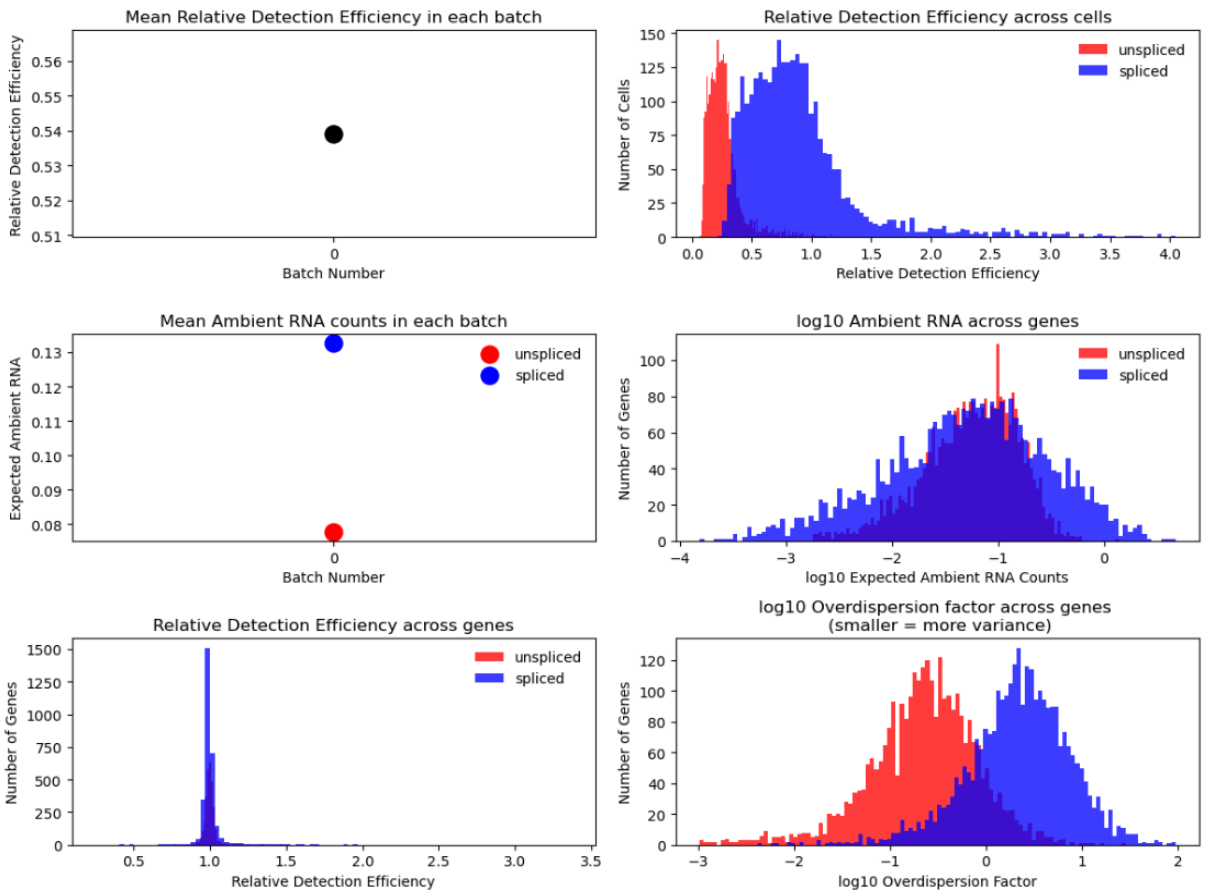


**Supp. Figure 34: overview of noise and technical variables for mouse dentate gyrus data**

*Unspliced counts have lower detection efficiency (top right), higher ambient RNA (middle right) and more noise than spliced counts, corresponding to a lower overdispersion parameter (lower right).*

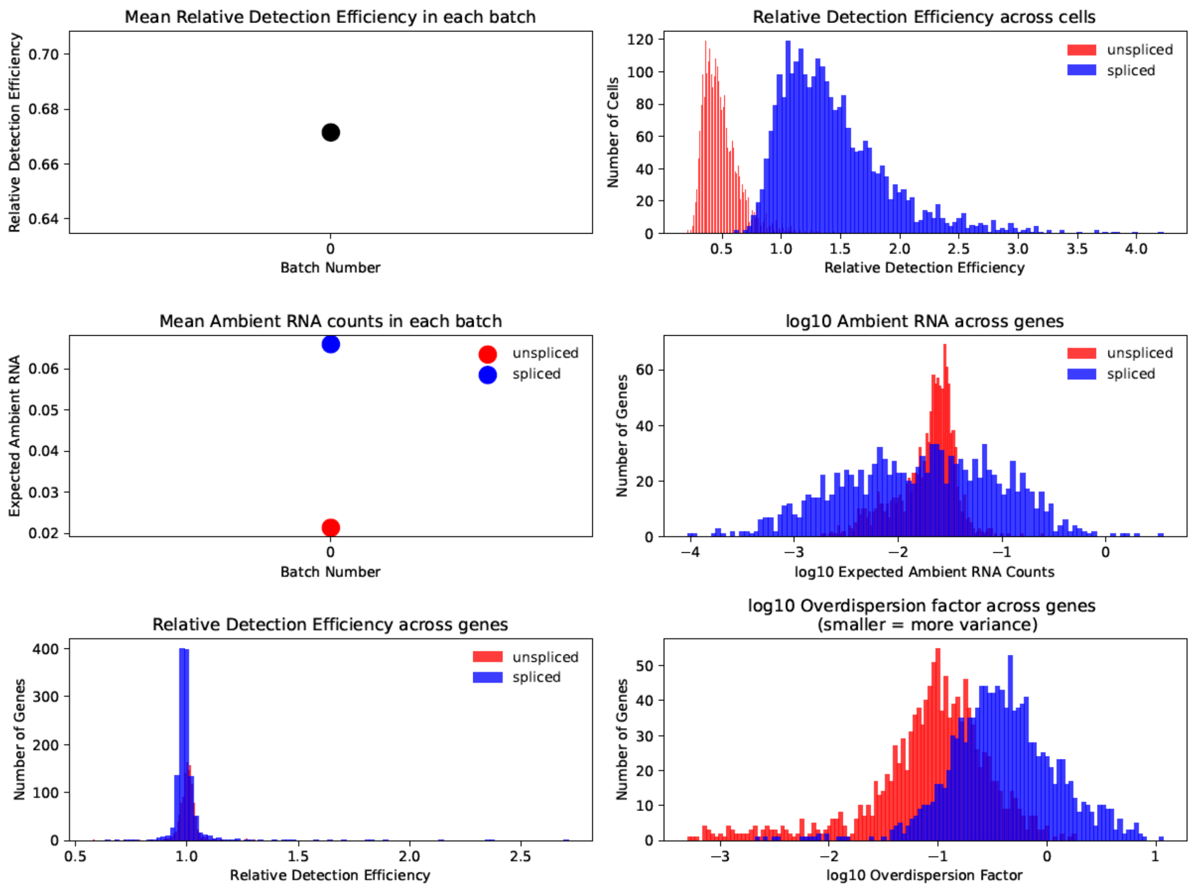


**Supp. Figure 35: overview of noise and technical variables for mouse bone marrow data**

*Unspliced counts have lower detection efficiency (top right), higher ambient RNA (middle right) and more noise than spliced counts, corresponding to a lower overdispersion parameter (lower right).*

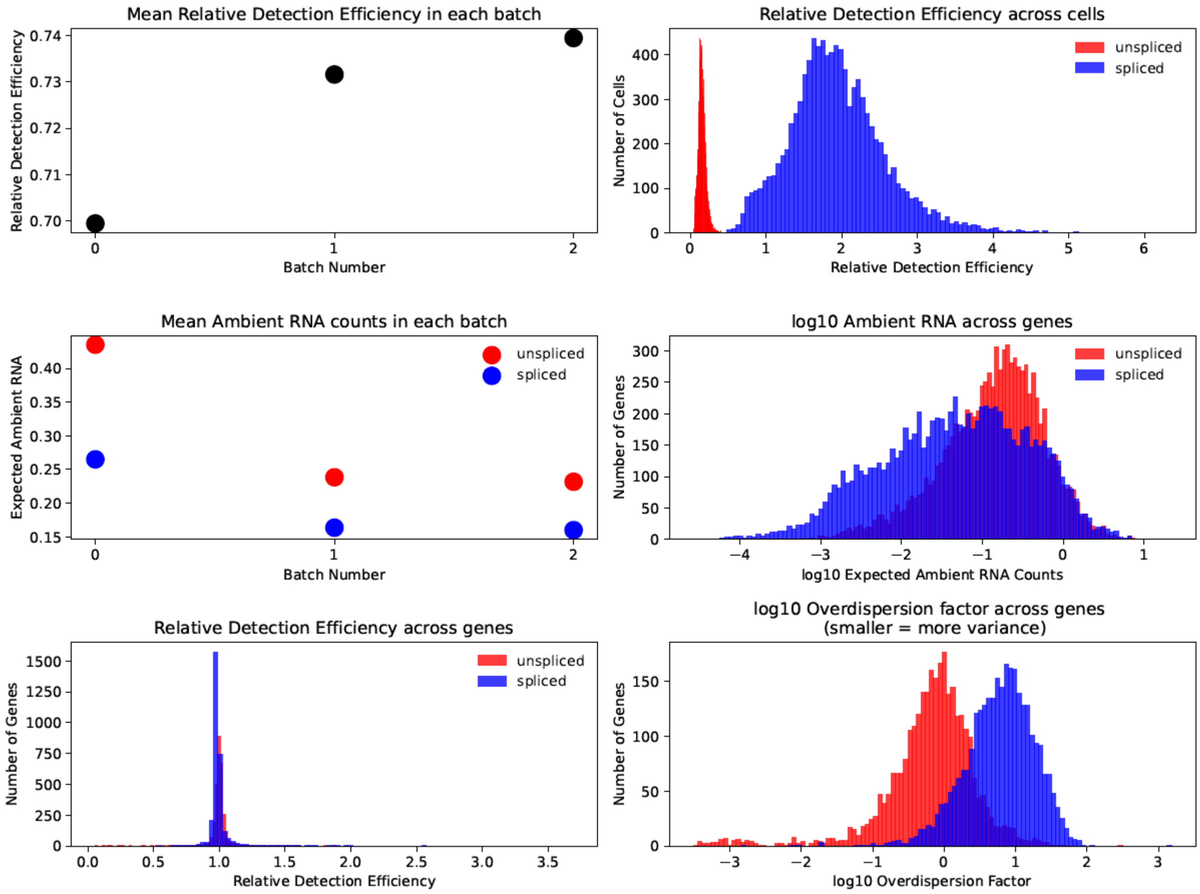


**Supp. Figure 36: overview of noise and technical variables for mouse erythroid maturation data**
*Unspliced counts have lower detection efficiency (top right), higher ambient RNA (middle right) and more noise than spliced counts, corresponding to a lower overdispersion parameter (lower right).*

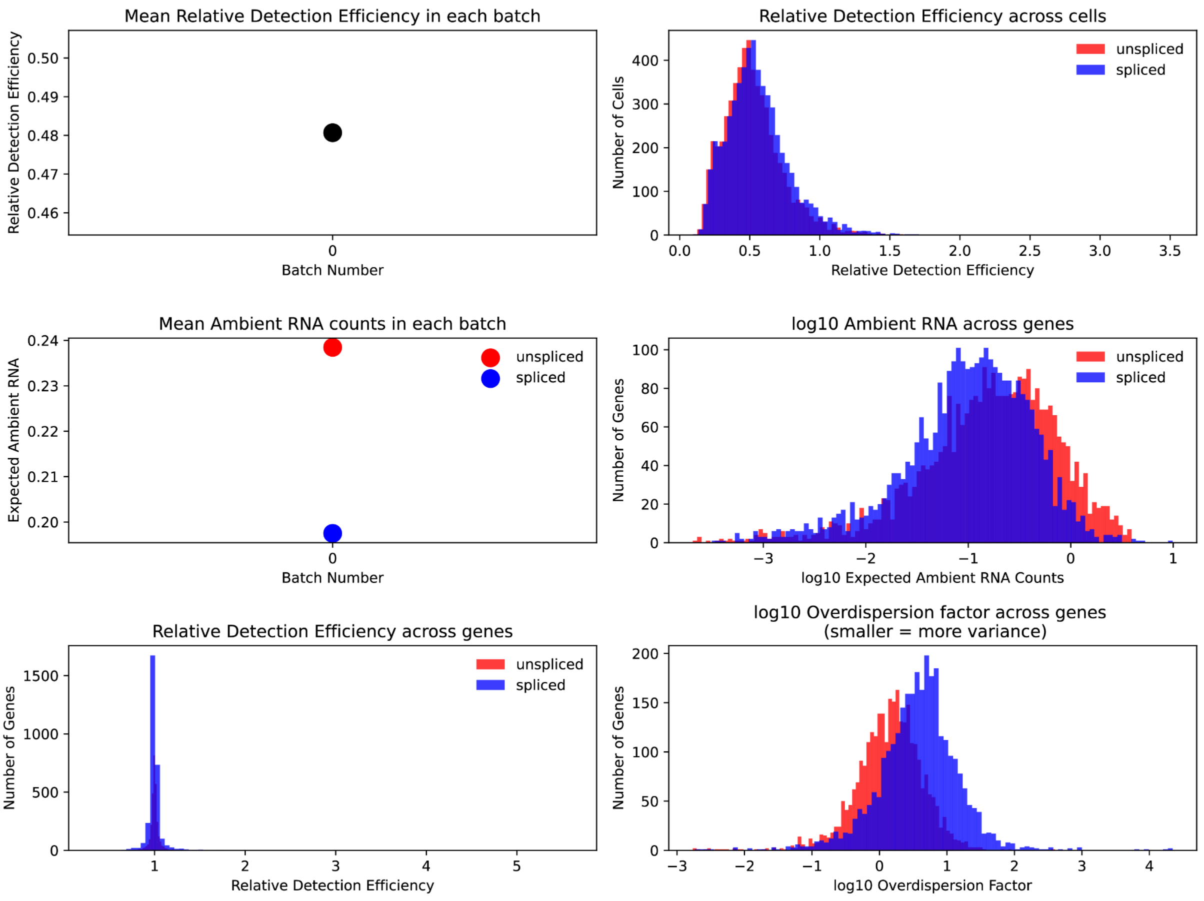


**Supp. Figure 37: overview of noise and technical variables for human bone marrow data**

*Unspliced counts have lower detection efficiency (top right), higher ambient RNA (middle right) and more noise than spliced counts, corresponding to a lower overdispersion parameter (lower right).*

# cell2fate Supplementary Notes

## Contents

**1 Cell2fate model description** **2**
1.1 Background and introduction to the RNA velocity problem . . . 2
1.2 Definition of cell2fate parameters and subscripts . . . 4
1.3 cell2fate biological model definition by linearization . . . 5
1.4 Measurement model . . . 9
1.5 Full Likelihood . . . 10
1.6 Prior distributions . . . 10
1.7 Interpretation of the linearization solution as mixed membership model . . . 14
1.8 Model inference and choice of hyperparameters . . . 15

**2 Comparative analysis of RNA velocity models** **16**
2.1 Cell-specific time . . . 16
2.2 Non-steady states, complex transcription rate function, cell-specific degradation and splicing rates . . . 17
2.3 Parameter estimation . . . 19
2.4 Multiple dynamics . . . 19
2.5 Stochastic fate decisions . . . 19
2.6 Mechanistic basis for switch time and rate parameters . . . 20
2.7 Low-rank modelling of gene switch times, transcription rates or counts . . . 20
2.8 Closed-form solution to count time evolution . . . 20
2.9 Raw Count Input . . . 21
2.10 Negative Binomial Noise, Batch Correction, Ambient RNA, Detection Probabilities . . . 21
2.11 Regularization via Hierarchical Priors . . . 21

**3 Appendix** **21**
3.1 Derivation of transcription rate, spliced and unspliced counts as a function of time . . . 21
3.2 Effect of mRNA detection probabilities on observed dynamics . . . 23
3.3 Approximate time until steady-state spliced counts abundance . . . 23
3.4 Connection to non-negative matrix factorization . . . 24
3.5 Potential Extensions . . . 24
3.5.1 Linking transcription rate modules by a regulatory model . . . 24

3.5.2 Extension to model stochastic fate decisions . . . . . . . . 25
3.5.3 Extension to variable splicing and degradation rates . . . 26
3.6 List of all components in the cell2fate generative model . . . . . 26

# 1 Cell2fate model description

## 1.1 Background and introduction to the RNA velocity problem

RNA velocity describes the rate of change of RNA in a cell [12], thereby giving rise to a dynamical, i.e. time-dependent models of gene-expression. Popular RNA velocity models to date, such as velocyto [12] and scvelo [1], are fit to spliced and unspliced scRNA-seq counts. The temporal information in the splicing process then allows estimating the direction and rate of expression changes across cells. To achieve this, RNA velocity models formulate a function for the rate of change of mRNA molecules over time. The first methods [12, 1] constructed this function by assuming that each gene produces mRNA molecules with a time-dependent gene-specific rate $\alpha_g(t)$, unspliced molecules, $u_{cg}$, are modified into spliced molecules, $s_{cg}$, with a constant gene-specific rate $\beta_g$ and degraded with a constant gene-specific rate $\gamma_g$, as summarized below:

$$\emptyset \xrightarrow{\alpha_g(t)} u_{cg} \xrightarrow{\beta_g} s_{cg} \xrightarrow{\gamma_g} \emptyset \tag{1}$$

For modelling this process, these differential equations have been suggested, which correspond to the first-order approximation of the chemical master equation of the process [20]:

$$\frac{du_g}{dt} = \alpha_g(t) - \beta_g u_g \tag{2}$$

$$\frac{ds_g}{dt} = \beta_g u_g - \gamma_g s_g, \tag{3}$$

with a step-wise transcription rate that depends on a cell and gene specific time, $t_{cg}$, and gene-specific switch times $t_{gON}$ and $t_{gOFF}$ [12, 1]:

$$\alpha_g(t) = \begin{cases} \alpha_g & \text{if } t_{cg} > t_{gON} \text{ and } t_{cg} < t_{gOFF} \\ 0 & \text{otherwise} \end{cases}$$

With such a step-wise transcription rate function, the differential equations can be solved analytically [1], which gives rise to time-evolution of spliced and unspliced counts with parameters that can in principle be fit to the observed data. Parameter fitting is challenging, because such RNA velocity models contain a large number of parameters, and spliced and unspliced raw counts include a great deal of technical noise. As a consequence, most methods use various tricks so that models are not directly fit to raw counts. This includes

normalizing by total counts in each cell and averaging counts across nearest neighbours in PCA space before parameter fitting. [1].

As noted previously [8, 2], while computationally convenient and tractable, this RNA velocity formulation makes coarse simplifications about the underlying transcriptional dynamics:

1. all rates are deterministic
2. the splicing and degradation rates are constant
3. splicing occurs in a single step
4. the transcription rate can only change to a single non-zero value
5. there is no interaction between transcription at different time points (no gene regulation)
6. genes specific rates are on different time-scales

In addition to these conceptual simplifications, the count-preprocessing, implies the following assumptions:

7. total cell-counts are proportional to count detection probabilities
8. there is no ambient RNA in cells
9. there are no batch effects
10. nearest neighbours in PCA space can be treated as nearest neighbours in time

These limitations and assumptions have also been implicated with the variable performance of velocito and scvelo [8, 2] for predicting cell fates.

Following these seminal implementations of RNA velocity, efforts have been directed towards improving the inference procedure [9, 7, 14] or fitting more realistic differential equation models [6], including cell-specific transcription rates [5, 9] and variable splicing and degradation rates [4]. Since the resulting complex systems of differential equation are not analytically tractable, current strategies are based on numerically approximating their solutions, which can impair computational scalibility, accuracy, statistical power and interpretability (see figure 2 in main text and section 1.8, “Comparative analysis of RNA velocity methods”).

Cell2fate follows a different route and is based on a new formulation of the RNA velocity problem (see section 1.3), which can overcome the limitations listed above without the need for numerical solvers in more complex RNA velocity models. Specifically, we suggest to approximate non-integrable functions, by a sum of integrable components, which we call modules. We use this principle to construct an analytically solveable RNA velocity model with arbitrarily varying transcription rate over time (section 1.3). In addition, we formulate

a comprehensive measurement model for spliced and unspliced counts that dispenses with the need for pre-processing counts (section 1.4). This model is then implemented in the probabilistic programming language pyro [3], so that parameters can be obtained via stochastic variational inference (sections 1.6, 1.8). We also give an outlook in the appendix (section 3.5) for how the remaining simplifications can be removed using this linearization approach in the near future.

## 1.2 Definition of cell2fate parameters and subscripts

Symbols for observed data, model parameters and subscripts are defined and explained in detail throughout the text. They are also listed here for additional clarity.

We first define subscripts, which are used to maintain clarity in the dimensions of observed data and model parameters. Capital letters denote the maximum value of the index.

- $c$ cells, $C$
- $g$ all genes, $G$
- $i$ states (ON or OFF), $I$
- $m$ modules, $M$
- $j$ mRNA maturity (unspliced or spliced), $J$
- $e$ experimental batch (usually separate 10X scRNAseq reactions), E

The following symbols denote observed data:

- $U_{cg}$ unspliced count matrix
- $S_{cg}$ spliced count matrix
- $X_{cgj}$ combined count matrix (spliced and unspliced)
- $H_{ce}$ a one-hot categorical assignment of cells to known experimental batches

The following symbols denote biological model parameters. Time-dependent parameter are either denoted with an additional "$(t)$" or equivalently by adding an additional $_c$ subscript, since time is a cell-specific parameter.

- $T_c$ the time of each cell
- $u_g(t)$, $u_{cg}$ biologically expected unspliced counts
- $s_g(t)$, $s_{cg}$ biologically expected spliced counts
- $x^B_{gj}(t)$, $x^B_{cgj}$ biologically expected unspliced and spliced counts

- $\alpha_g(t)$, $\alpha_{cg}$ the transcription rate of a gene
- $\beta_g$ the splicing rate of a gene
- $\gamma_g$ the degradation rate of a gene
- $v_g(t)$ the RNA velocity of a gene
- $\alpha_{mg}$ transcription rate of each module
- $\hat{\alpha}_{mgi}$ the target transcription rate of each module
- $\lambda_{mi}$ rate of transcription rate change for each module
- $T_{mON}$ switch ON time of each module
- $T_{mOFF}$ switch OFF time of each module
- $A_{mgON}$ target transcription rate in the ON state

These are the measurement model parameters:

- $s_{egj}$ ambient RNA counts
- $l_{cgj}$ detection efficiency
- $x^M_{cgj}$ expected counts after accounting for measurement noise
- $a_{gj}$ Negative Binomial overdispersion parameter

## 1.3 cell2fate biological model definition by linearization

Following previous approaches, we aim to model RNA velocity with more general differential equations, in which the transcription rate is itself going through dynamic changes parameterized here by a function $F_\alpha(t)$:

$$\frac{d\alpha_g}{dt} = F_\alpha(t) \tag{4}$$

$$\frac{du_g}{dt} = \alpha_g(t) - \beta_g u_g \tag{5}$$

$$\frac{ds_g}{dt} = \beta_g u_g - \gamma_g s_g, \tag{6}$$

where RNA velocity $v(t)$ is the time-derivative of spliced mRNA:

$$v_g(t) = \frac{ds_g}{dt} \tag{7}$$

Gene transcription rates are generally believed to be a complex non-linear function of changes in active transcription factor abundance in the nucleus [19], where activation states of transcription factors can be driven by signalling events from neighbouring cells [17]. At the core of cell2fate is the concept

to approximate this complexity with a linear sum of easily integrable basis functions:

$$F_\alpha(t) = \sum_{m=1}^{M} \frac{d\alpha_{mg}}{dt} = \sum_{m=1}^{M} \lambda_{mi}(\hat{\alpha}_{mgi} - \alpha_{mg}) \tag{8}$$

Each component in the sum is denoted by a subscript $m$ and is termed a *module* in the following. The state index $i$ can take on the two values $ON$ and $OFF$ (active or inactive state), depending on a time assigned to each cell, $T_c$, and so-called module switch-times, $T_{mON}$, $T_{mOFF}$:

$$i = \begin{cases} ON & \text{if } T_c > T_{mON} \text{ and } T_c < T_{mOFF} \\ OFF & \text{otherwise} \end{cases}$$

The parameter $\hat{\alpha}_{mgi}$, which we call the target transcription rate takes on the following values in each state:

$$\hat{\alpha}_{mgi} = \begin{cases} A_{mgON} & \text{if } i = ON \\ 0 & \text{if } i = OFF \end{cases}$$

The parameters $T_c$, $T_{mON}$, $T_{mOFF}$, $\lambda_{mi}$ and $A_{mgON}$ are determined from the data with prior distributions defined in section 1.6. We physically interpret the transcription rate function by postulating that within small time windows defined by $T_{mON}$, $T_{mOFF}$, changes in the transcription rate are driven by changes in only a small number of active regulatory proteins (transcription factors or co-factors) that simultaneously increase or decrease in abundance. The transcription rate function captures the effects of such increases or decreases up to a saturation point defined by $\hat{\alpha}_{mgi}$.

With this linearized function for the transcription rate dynamics $F_\alpha(t)$, the time evolution for $u_g$ and $s_g$ can be decomposed into a sum of effects arising from each module, which gives rise to these new RNA velocity differential equations:

$$\frac{d\alpha_g}{dt} = \sum_{m=1}^{M} \frac{d\alpha_{mg}}{dt} = \sum_{m=1}^{M} \lambda_{mi}(\hat{\alpha}_{mgi} - \alpha_{mg}) \tag{10}$$

$$\frac{du_g}{dt} = \sum_{m=1}^{M} \frac{du_{mg}}{dt} = \sum_{m=1}^{M} (\alpha_{mg} - \beta_g u_{mg}) \tag{11}$$

$$\frac{ds_g}{dt} = \sum_{m=1}^{M} \frac{ds_{mg}}{dt} = \sum_{m=1}^{M} (\beta_g u_{mg} - \gamma_g s_{mg}) \tag{12}$$

This also defines a velocity for each module, $v_{mg}$:

$$v_{mg} = \frac{ds_{mg}}{dt}, \tag{13}$$

where the total velocity is the sum of the module velocities.

The analytical solution for the transcription rate and spliced and unspliced counts as a function of time is derived in 3.1 and is given by:

$$\alpha_g(T_c) = \sum_m \alpha_{mg}(T_c) = \sum_M (\alpha^0_{mgi} + \delta^\alpha_{mgi}(1 - e^{-\lambda_{mi}\tau_{cmi}})) \tag{14}$$

$$\mathrm{u}_g(T_c) = \textstyle\sum_m u_{mg}(T_c) = \sum_m (u^0_{mgi} e^{-\beta_g \tau_{cmi}} + \dfrac{\alpha^0_{mgi}}{\beta_g}(1 - e^{-\beta_g \tau_{cmi}}) + \dfrac{\delta^\alpha_{mgi}}{\beta_g - \lambda_{mi}}(e^{-\beta_g \tau_{cmi}} - e^{-\lambda_{mi}\tau_{cmi}})) \tag{15}$$

$$\mathrm{s}_g(T_c) = \textstyle\sum_m s_{mg}(T_c) = \sum_m (s^0_{mgi} e^{-\gamma_g \tau_{cmi}} + \dfrac{\alpha^0_{mgi}}{\gamma_g}(1 - e^{-\gamma_g \tau_{cmi}}) + \dfrac{\alpha^0_{mgi} - \beta_g u^0_{mgi}}{\gamma_g - \beta_g}(e^{-\gamma_g \tau_{cmi}} - e^{-\beta_g \tau_{cmi}}) + \dfrac{\delta^\alpha_{mgi}\beta_g}{(\beta_g - \lambda_{mi})(\gamma_g - \beta_g)}(e^{-\beta_g \tau_{cmi}} - e^{-\gamma_g \tau_{cmi}}) + \dfrac{\delta^\alpha_{mgi}\beta_g}{(\beta_g - \lambda_{mi})(\gamma_g - \lambda_{mi})}(e^{-\lambda_{mi} \tau_{cmi}} - e^{-\gamma_g \tau_{cmi}})), \tag{16}$$

with:

$$\tau_{cmi} = T_c - T_{mi} \tag{17}$$

$$\delta^\alpha_{mgi} = \hat{\alpha}_{mgi} - \alpha^0_{mgi} \tag{18}$$

and the following initial conditions:

$$\alpha^0_{mgON} = 0 \tag{19}$$

$$u^0_{mgON} = 0 \tag{20}$$

$$s^0_{mgON} = 0 \tag{21}$$

$$\alpha^0_{mgOFF} = \alpha_{mg}(T_{mOFF}) \tag{22}$$

$$u^0_{mgOFF} = u_{gm}(T_{mOFF}) \tag{23}$$

$$s^0_{mgOFF} = s_{gm}(T_{mOFF}) \tag{24}$$

We can express these dynamical equations more compactly by renaming terms that reoccur in the equations, as follows:

$$A = e^{-\lambda_{mi}\tau_{cmi}} \tag{25}$$

$$B = e^{-\beta_g\tau_{cmi}} \tag{26}$$

$$C = e^{-\gamma_g\tau_{cmi}} \tag{27}$$

$$D = B - C \tag{28}$$

$$\theta_{mgi} = \beta_g - \lambda_{mi} \tag{29}$$

$$\xi_{mgi} = \frac{\delta^\alpha_{mgi}\beta_g}{\theta_{mgi}} \tag{30}$$

$$\eta_g = \gamma_g - \beta_g, \tag{31}$$

which results in:

$$\alpha_g = \sum_M \left(\alpha^0_{mgi} + \delta^\alpha_{mgi}(1 - A)\right) \tag{32}$$

$$u_g = \sum_M \left(u^0_{mgi}B + \frac{\alpha^0_{mgi}}{\beta_g}(1 - B) + \frac{\xi_{mgi}}{\beta_g}(B - A)\right) \tag{33}$$

$$s_g = \sum_M \left(s^0_{mgi}C + \frac{\alpha^0_{mgi}}{\gamma_g}(1 - C) + \left(\alpha^0_{mgi} + \beta_g u^0_{mgi} + \xi_{mgi}\right)\frac{D}{\eta_g} + \frac{\xi_{mgi}}{(\gamma_g - \lambda_{mi})}(A - C)\right) \tag{34}$$

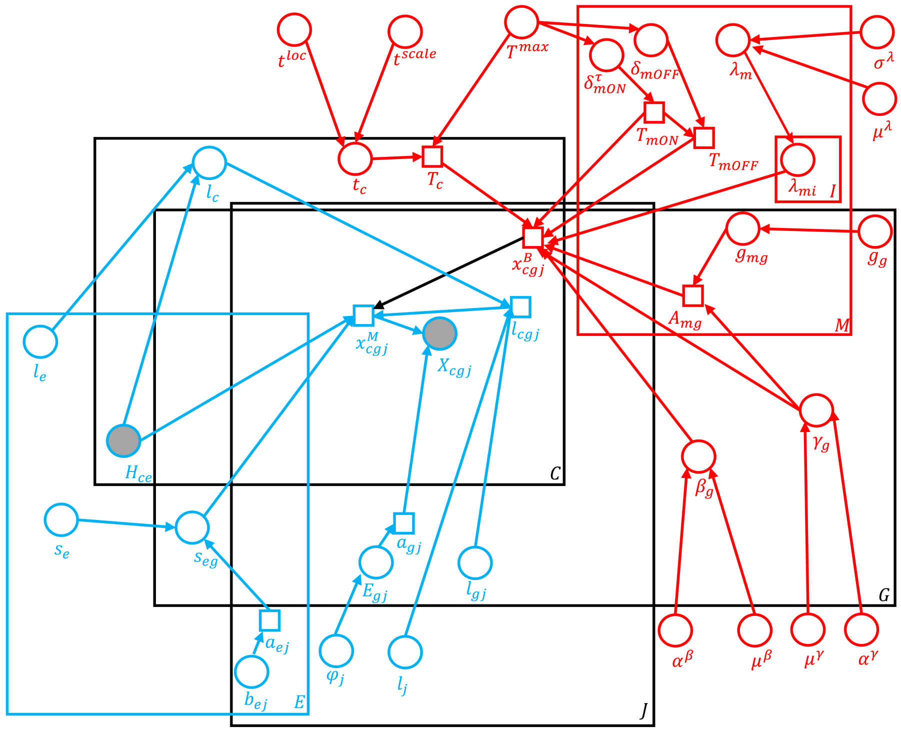


Figure 1: Summary of the generative model in a plate diagram. Circular nodes denote random variables and square nodes denote variables that are deterministically computed from other nodes. Red elements are exclusive to the "biological model" and blue elements are exclusive parts of the "measurement model."

### 1.4 Measurement model

We take into account technical factors of variation that change the expectation values for measured quantitites of spliced and unspliced counts by transforming "biological" expectation values $x^B_{cgj} = (u_{cg}, s_{cg})$ to account for technical variables:

$$x^M_{cgj} = l_{cgj} * (H_{ce} * s_{egj} + x^B_{cgj}) \tag{35}$$

Here, $H_{ce}$ denotes a one-hot categorical assignment of cells to experimental batches, $l_{cgj}$ describes differences in detection efficiency (e.g., sequencing depth, read alignment and quantification) of genes across cells and $s_{gej}$ models ambient RNA ("soup") for each gene in each batch. These "measurement" expectation values are then used to parameterize a Negative Binomial observation model of the observed raw count values $X_{cgj} = (U_{cg}, S_{cg})$:

$$X_{cgj} \sim \texttt{NegBinom}(\mu = x^M_{cgj}, \alpha = a_{gj}) \tag{36}$$

Here, $a_{jg}$ are Negative Binomial over-dispersion parameters for each gene, separate for spliced and unspliced counts. A Poisson distribution alone can model the sampling noise of transcripts, which is added during measurement [16]. The extra variance added by the Negative Binomial distribution over the Poisson distribution models additional sources of noise [16]. This includes the variance arising from stochastic rates (i.e. "transcriptional bursting") [16]. While prior work [13, 10] has shown that stochastic rates result in time-varying variances and co-variances for spliced and unspliced counts, we chose not consider this additional complexity for now, because we expect this additional model refinement to have only a small effect on accuracy. Finally, the extra Negative Binomial overdispersion should also account for missing details in our model (i.e. "model uncertainty"), such as the addition of other dynamical processes on top of the main process (e.g. the cell cycle). We chose separate overdispersion parameters for spliced and unspliced counts, since measurement noise is expected to be different for spliced and unspliced counts due to differences in how they are detected and quantified.

### 1.5 Full Likelihood

In summary, the full likelihood of the model is:

$$\prod_C \prod_G \prod_J P(X_{cgj}|\theta_{cgj}) = \prod_C \prod_G \prod_J \mathtt{f}_{\mathtt{NB}}(\mu = F(\theta_{cgj}), \alpha = a_{gj}), \tag{37}$$

where $\theta_{cgj}$ are all model parameters and $\mathtt{f}_{\mathtt{NB}}$ is the probability density function of the Negative Binomial distribution.

$$\theta_{cgj} = (l_{cgj}, H_{ce}, s_{egj}, T_c, \beta_g, \gamma_g, A_{mgON}, \lambda_{mi}, T_{mi}) \tag{38}$$

and $F$ is a function that summarizes the dependence of expected measured counts $x^M_{cgj}$ on all model parameters, through equations 33, 34, 35.

### 1.6 Prior distributions

The full Bayesian posterior of the model, $P(\theta_{cgj}|X_{cgj})$, is obtained by combining the likelihood above, $P(X_{cgj}|\theta_{cgj})$, with the prior distributions for each parameter, $P(\theta_{cgj})$:

$$P(\theta_{cgj}|X_{cgj}) = P(X_{cgj}|\theta_{cgj})P(\theta_{cgj}) \tag{39}$$

We list and motivate these prior distributions below.

We derived analytically (Appendix 3.3) that most genes in a module will reach their steady state after 20 hours. This sets a general order of magnitude for the maximal time $T^{max}$ of a process that we can expect. We choose this prior for the maximal time:

$$T^{max} \sim \mathtt{Gamma}(\mu = 50, \sigma = 50) \tag{40}$$

Based on this, the prior for the cell process time is:

$$t^{loc} \sim \texttt{Gamma}(\alpha = 1, \beta = \frac{1}{0.5}) \tag{41}$$

$$t^{scale} \sim \texttt{Gamma}(\alpha = 1, \beta = \frac{1}{0.25}) \tag{42}$$

$$t_c \sim \texttt{Normal}(\mu = t^{loc}, \sigma = t^{scale}) \tag{43}$$

$$T_c = t_c * T^{max} \tag{44}$$

In this parameterization of the Gamma distribution with $\alpha$ and $\beta$ instead of $\mu$ and $\sigma$, $\alpha$ controls the variance (larger $\alpha$ means less variance) and $\beta$ can be set to $\frac{\alpha}{\mu}$ to control the mean. Hence, values for $t_c$ will largely fall between 0 and 1, so that the overall magnitude of $T_c$ is controlled by $T^{max}$ .

Switch-on times of all modules $m > 1$ are defined by a time gap to the switch-on time of the previous module:

$$\delta^{\tau}_{mON} \sim \begin{cases} 0 & \text{if } m = 1 \\ T^{max} \cdot \texttt{Gamma}(\alpha = 20, \beta = 20 * M) & \text{otherwise} \end{cases}$$

$$T_{mON} = \sum_{m'=1}^{m} \delta^{\tau}_{m'ON} \tag{45}$$

Switch-off times of all modules are assumed to depend on occurrence of a signal with uniform probability of appearing at each point in time, so that an exponential distribution is appropriate:

$$\delta^{\tau}_{mOFF} \sim \texttt{Exponential}\left(\frac{M}{T^{max}}\right) \tag{46}$$

$$T_{mOFF} = T_{mON} + \delta^{\tau}_{mOFF} \tag{47}$$

Moving on to the prior distributions for our rate parameters, the mean degradation rate is expected to be 0.2 (molecules/h) and the mean splicing rate is expected to be about 5 times as fast, based on previous work [15]. We chose to set uninformative priors in this general order of magnitude

$$\alpha^{\gamma} \sim \texttt{Gamma}(\alpha = 20, \beta = \frac{20}{5}) \tag{48}$$

$$\mu^{\gamma} \sim \texttt{Gamma}(\alpha = 10, \beta = \frac{10}{1}) \tag{49}$$

$$\gamma_g \sim \texttt{Gamma}(\alpha = \alpha^{\gamma}, \beta = \frac{\alpha^{\gamma}}{\mu^{\gamma}}) \tag{50}$$

$$\alpha^{\beta} \sim \texttt{Gamma}(\alpha = 20, \beta = \frac{20}{5}) \tag{51}$$

$$\mu^{\beta} \sim \texttt{Gamma}(\alpha = 10, \beta = \frac{10}{1}) \tag{52}$$

$$\beta_g \sim \texttt{Gamma}(\alpha = \alpha^{\beta}, \beta = \frac{\alpha^{\beta}}{\mu^{\beta}}) \tag{53}$$

The rates of activation for each module, share a hierarchical prior like this:

$$\mu^{\lambda} \sim \texttt{Gamma}(\mu = 1, \sigma = 0.33) \tag{54}$$

$$\sigma^{\lambda} \sim \texttt{Gamma}(\mu = 0.33, \sigma = 0.1) \tag{55}$$

$$\lambda_m \sim \texttt{Gamma}(\mu = \mu^{\lambda}, \sigma = \sigma^{\lambda}) \tag{56}$$

$$\lambda_{mi} \sim \texttt{Gamma}(\mu = \lambda_m, \sigma = \lambda_m * 0.05) \tag{57}$$

Instead of defining the maximal effect on the transcription rate by each module directly, we define the maximal effect on spliced counts $g_{mg}$ (at $\tau = \infty$) and multiply this by the degradation rate to obtain the effect on the transcription rate:

$$A_{mg} = g_{mg} \cdot \gamma_g \tag{58}$$

Priors for $g_{mg}$ are given by:

$$g_g \sim \texttt{Gamma}(\alpha = 1, \beta = 1) \tag{59}$$

$$g_{mg} \sim \texttt{Gamma}(\alpha = \frac{h}{M}, \beta = \frac{1}{g_g}) \tag{60}$$

$$h = 3 \tag{61}$$

$g_g$ can be interpreted as the total counts produced by a gene in a cell per module in the steady state. $h$ can be interpreted as the number of modules this gene is part of, since the mean per module is given by:

$$\texttt{mean}(g_{mg}) = \frac{h \cdot g_g}{M} \tag{62}$$

Moving on to technical variables, we use this hierarchical prior for the overdispersion parameter of spliced and unspliced counts:

$$a_{gj} = \frac{1}{E_g^2} \tag{63}$$

$$E_{gj} \sim \texttt{Exponential}(\phi) \tag{64}$$

$$\phi_j \sim \texttt{Gamma}(\mu = 3, \sigma = 1) \tag{65}$$

This kind of prior is called a containment prior [18]. To understand the reasoning behind it, it is useful to write out the total variance of the Negative Binomial distribution:

$$\sigma^2 = \mu + \frac{\mu^2}{a}, \tag{66}$$

so the chosen prior pushes alpha towards infinity and with that the "extra variance" compared to a Poisson distribution towards 0. So inference should only converge to solutions with considerable overdispersion if this is really necessary.

The mean relative detection probability in each cell depends on the experimental batch $e$ each cell comes from:

$$l_e \sim \texttt{Beta}(\alpha = 1, \beta = 1) \tag{67}$$

(This results in a broad distribution between 0 and 1 with mean of 0.5.)

$$l_c \sim \texttt{Gamma}(\alpha = 10, \beta = \frac{10}{H_{ce} * l_e}) \tag{68}$$

To model differences in detection probabilities for spliced and unspliced counts, we define two more parameters that describe their average difference (using a broad prior around 1):

$$l_j \sim \texttt{Gamma}(\alpha = 100, \beta = \frac{100}{1}), \tag{69}$$

as well as their gene specific difference (using a narrow prior):

$$l_{gj} \sim \texttt{Gamma}(\alpha = 200, \beta = \frac{200}{1}) \tag{70}$$

That together give the gene- and cell-specific detection probability for spliced and unspliced counts:

$$l_{cgj} = l_c \cdot l_j \cdot l_{gj} \tag{71}$$

Finally, we use this containment prior for the ambient RNA parameter of both spliced and unspliced counts:

$$s_e \sim \texttt{Gamma}(\mu = 0.005, \sigma = 0.00005) \tag{72}$$

$$b_{ej} \sim \texttt{Exponential}(\lambda^b = 9) \tag{73}$$

$$a^s_{ej} = \frac{1}{b^2_{ei}} \tag{74}$$

$$s_{egj} \sim \texttt{Gamma}(\alpha = a^s_{ej}, \beta = \frac{a^s_{ej}}{s_e}) \tag{75}$$

## 1.7 Interpretation of the linearization solution as mixed membership model

The cell2fate model can be parameterized as a mixed membership model, by factoring out the target transcription rate in the on state $A_{mgON}$ from the time evolution of the total transcription rate (equation 14), like this:

$$\alpha_g(T_c) = \sum_M A_{mgON} \cdot \alpha'_{mg}(T_c) \tag{76}$$

The transcription rate of each module is similar as before:

$$\alpha'_{mg}(T_c) = \sum_M (\alpha^0_{mgi} + \delta'^{\alpha}_{mgi}(1 - e^{-\lambda_{mi}\tau_{cmi}})) \tag{77}$$

with:

$$\delta'^{\alpha}_{mgi} = \hat{\alpha}'_{mgi} - \alpha^0_{mgi} \tag{78}$$

$$\alpha^0_{mgON} = 0 \tag{79}$$

$$\alpha^0_{mgOFF} = \alpha'_{mg}(T_{mOFF}) \tag{80}$$

The only difference is that the target transcription rate is now capped at 1:

$$\hat{\alpha}_{mgi}(t)' = \begin{cases} 1 & \text{if } i = ON \\ 0 & \text{if } i = OFF, \end{cases}$$

With this parameterization, $A_{mgON}$ corresponds the "gene loadings" visualized as bars in figure 1B of the main text and $\alpha'_{mg}(T_c)$ defines the temporal patterns, illustrated at the top of figure 1B, which are similar to "cell loadings" in PCA and non-negative matrix factorization. See appendix 3.4 for further comparisons to non-negative matrix factorization.

## 1.8 Model inference and choice of hyperparameters

The model is implemented in the probabilistic programming language pyro [3]. All parameters are initialized to the mean of their prior distributions. Inference is performed using stochastic variational inference (SVI), using the "AutoHierarchical NormalMessenger" autoguide and Adam optimizer [11] for 500 iterations with a learning rate of 0.01 and a batch size of 1,000 cells by default. Posteriors are then estimated numerically by sampling 100 values from the variational distribution. The parameter controlling the maximal number of modules, $M$, is chosen before training by Louvain clustering of the data and multiplying the total number of clusters by 1.15. For the datasets included in our study this optimization finished in under 20 minutes in all cases (see figure 2).

In more detail, within the SVI inference framework, the posterior distributions over unknown parameters are approximated by univariate normal distributions that are transformed to ensure the appropriate scale for each parameter (for example, positive for the mean of a Gamma distribution). The parameters of these variational distributions are then determined by minimizing the KL divergence between the variational approximation and the true posterior distribution or, equivalently, maximizing the evidence lower bound (ELBO loss function). The use of the "AutoHierarchical NormalMessenger" autoguide improves the variational approximation, by keeping dependencies between parameters as present in the model through hierarchical prior distributions. Specifically, the mean-field posterior at any site is a transformed normal distribution, the mean of which depends on the value of that site given its dependencies in the model:

$$\underbrace{x}_{\text{Variable}} = \texttt{transform}(\texttt{Normal}(\underbrace{\mu}_{\text{Total mean}}, \underbrace{\sigma}_{\text{Independent standard deviation}})), \tag{82}$$

$$\underbrace{\mu}_{\text{Total mean}} = \underbrace{loc}_{\text{Independent mean}} + \underbrace{\texttt{transform.inverse}(\hat{\mu})}_{\text{Prior mean transformed to real value space}} \cdot \underbrace{weight}_{\text{Weight}}, \tag{83}$$

which is equivalent to defining a posterior distribution conditional on the hierarchical prior of each variable, where `transform` indicates a function that maps the posterior distribution in real value space to the domain in which the variable is defined.

| Dataset | No of cells | No of genes | No of modules | Run time (min:sec) |
|---|---|---|---|---|
| Dentate Gyrus | 2930 | 3000 | 16 | 15:51 |
| Pancreas | 3669 | 3000 | 14 | 8:22 |
| Mouse Bone Marrow | 2600 | 3000 | 9 | 3:15 |
| Erythroid Maturation | 9815 | 3000 | 10 | 16:51 |
| Human Bone Marrow | 5780 | 3000 | 11 | 11:13 |
| Human Brain | 9443 | 3000 | 11 | 18:29 |

Figure 2: Overview of cell2fate training time on different datasets using a Tesla V100-SXM2 32GB GPU.

# 2 Comparative analysis of RNA velocity models

We provide a table in figure 3 that succinctly compares existing RNA velocity methods according to 17 criteria. We further explain and discuss those criteria below.

| | velocyto | scVelo | UniTVelo | DeepVelo | VeloVAE | VeloVI | LatentVelo | Pyro-Velocity | cell2fate |
|---|---|---|---|---|---|---|---|---|---|
| **Parameter Estimation** | Least-squares | Expectation Maximization | Expectation Maximization | Backpropagation | Variational Autoencoders | Variational Autoencoders | Variational Autoencoders | Stochastic Variational Inference in Pyro | Stochastic Variational Inference in Pyro |
| **Cell-specific time** | No | No | Yes | No | Yes | No | Yes | Yes | Yes |
| **Non-steady states** | No | Yes | Yes | Yes | Yes | Yes | Yes | Yes | Yes |
| **Complex transcription rate function** | No | No | Partial | Yes | Yes | Partial | Yes | No | Yes |
| **Cell-specific degradation and splicing rates** | No | No | No | Yes | No | No | No | No | No |
| **Multiple Dynamics** | Partial | Partial | No | Yes | Yes | Partial | Yes | Partial | Yes |
| **Stochastic Fate Choices** | Partial | Partial | No | No | Yes | Partial | No | Partial | No |
| **Mechanistic basis for switch time and rate parameters** | Partial | Partial | Partial | No | No | Partial | No | Partial | Yes |
| **Low-rank modelling of gene switch times** | No | No | No | No | No | Yes | No | No | Yes |
| **Low-rank modelling of transcription rates** | No | No | No | No | No | No | No | No | Yes |
| **Low-rank modelling of counts** | No | No | No | No | No | No | Yes | No | Yes |
| **Closed-form solution to count time evolution (= no numerical approximations)** | Yes | Yes | Yes | No | No | Yes | No | Yes | Yes |
| **Raw Count Input** | No | No | No | No | No | No | No | Yes | Yes |
| **Negative Binomial Noise** | No | No | No | No | No | No | No | No | Yes |
| **Batch correction** | No | No | No | No | No | No | Yes | No | Yes |
| **Ambient RNA** | No | No | No | No | No | No | No | No | Yes |
| **Detection Probabilities** | No | No | No | No | No | No | No | No | Yes |
| **Regularization via Hierarchical Priors** | No | No | No | No | No | No | No | No | Yes |

Figure 3: Comparison of RNA velocity methods in table format, according to parameter estimation procedures and model components.

## 2.1 Cell-specific time

Inuitively, each cell in a dataset can be thought of as occupying a unique process time point in a temporal dimensions, which we called $T_c$ in the cell2fate method. However, velocyto, scVelo and veloVI do not only estimate a separate time for each cell, but also for each gene, resulting in a two-dimensional time parameter

$T_{cg}$. $T_{cg} = 0$ is defined as the switch-on time of the gene in this case. In addition, a switch off time $T_{gOFF}$ is defined separately for each gene. While this approach allows more flexibility in modelling transcriptional dynamics, it increases the number of parameters that need to be estimated by a factor $G$ and thus reduces statistical power.

## 2.2 Non-steady states, complex transcription rate function, cell-specific degradation and splicing rates

In general, all methods use the deterministic differential equations describing the transcription, splicing and degradation process, introduced in equations 2 and 3. However, methods differ in how much complexity they allow in the transcription rate function. In addition, DeepVelo stands out as using cell-specific splicing and degradation rates, rather than just gene-specific ones.

The velocyto method solves the RNA velocity differential equations in 2 and 3, by making the assumption that $\frac{du_g}{dt}$ and $\frac{ds_g}{dt}$ are 0 and then fitting their model to cells with maximal and minimal expression for a given gene. All other methods relax this steady-state assumption and solve the equations in full and then fit their model to all data.

In velocyto, scvelo and pyro-velocity the transcription rate is a step-wise function of time. veloVI and UniTVelo relax this assumption slightly by allowing gradual increases and decreases of the transcription rate, but still with a single maximal value. DeepVelo, VeloVAE, LatentVelo and cell2fate fully relax constraints on transcription rates, by allowing arbitrary transcriptional patterns over time. The way in which the latter four methods achieve this goal is very different. In DeepVelo rate parameters of each cell $\alpha_{cg}$, $\beta_{cg}$, $\gamma_{cg}$ are defined as a function of each cell's gene-expression, $X_{cg}$ by a graph-convolutional network, $F$:

$$\alpha_{cg}, \beta_{cg}, \gamma_{cg} = F(X_{cg}) \tag{84}$$

This generates the velocity of each cell, according to the standard equation, as:

$$\frac{ds_{cg}}{dt} = \beta_{cg} u_{cg} - \gamma_{cg} s_{cg} \tag{85}$$

The expected spliced counts expression of each cell is then generated by:

$$s_{cg} = \frac{\sum_{c'=1}^{c'=C} \left( K_{cc'} \left( s_{c'g} + \delta^T \cdot \frac{ds_{c'g}}{dt} \right) \right)}{\sum_{c'=1}^{c'=C} K_{cc'}} \tag{86}$$

, where $\delta^T$ is a time-step that is set to 1 in the DeepVelo method and $K_{cc'}$ is a binary matrix that indicates matching temporal neighbours for each cell that is found as part of the optimization process. The most obvious assumption of this numerical approximation is thus that $\frac{ds_{cg}}{dt}$ is constant over an interval

$\delta^T = 1$. In addition, the approach relays on finding the neighbour matrix $K_{cc'}$ accurately.
VeloVAE has some similarity to the above numerical approach despite being implemented in a different framework, using variational autoencoders. In particular, each cell is assigned a state $c_c$, based on which a relative transcription rate $\rho_c$ between 0 and 1 is generated, by a neural network $F$, so that the total transcription rate is given by:

$$c_c \sim \texttt{Normal}(\mu = 0, \sigma = 1) \tag{87}$$

$$\rho_c = F(c_c) \tag{88}$$

$$\alpha_{cg} = \alpha_g \cdot \rho_c \tag{89}$$

Initial conditions for the differential equations are assumed to be $u_0 = s_0 = 0$ in a first training run. After the first run, the nearest temporal neighbours of each cell are then used to construct cell specific initial conditions $u_{c0}$, $s_{c0}$ for a second training run. Thus this approach assumes transcription rates are constant in the interval between nearest temporal neighbours and it relies on finding those temporal neighbours accurately.
LatenVelo is also based on variational autoencoders, but does not model gene-wise dynamics. Instead it models dynamics in the latent space for unspliced and spliced counts. For this purpose it defines three latent variables:

$$z_{cm} = (z^u_{cm}, z^s_{cm}, z^r_{cn}) \sim \texttt{Normal}(\mu = 0, \sigma = 1), \tag{90}$$

where $m \in 1, ..., M$ and $n \in 1, ..., N$ define the dimensions of latent parameters. $M = 20$ by default and N is adjusted to be the "expected number of lineages" minus 1. $(z^u_{cm}, z^s_{cm})$ are used to generated expected spliced and unspliced counts, as in a standard variational autoencoder:

$$u_{cg}, s_{cg} = F(z^u_{cm}, z^s_{cm}), \tag{91}$$

where $F$ is the auto-encoder neural network. In addition, all three latent parameters are constrained to obey the following dynamics:

$$t \sim \texttt{LogitNormal}(\mu = 0, \sigma = 1) \tag{92}$$

$$\frac{dz^u_{cm}}{dt} = f_u(z^u_{cm}, z^r_{cm}) \tag{93}$$

$$\frac{dz^s_{cm}}{dt} = f_s(z^u_{cm}, z^s_{cm}) \tag{94}$$

$$\frac{dz^r_{cm}}{dt} = f_r(z^s_{cm}, z^r_{cm}, h) \tag{95}$$

$$h = f_h(z^u_{cm}, z^s_{cm}), \tag{96}$$

where $f$ are neural networks. The rate of change of unspliced counts, thus depends on $z^r$ and $h$, so that this model can in principle capture complex dynamics that depend on the current latent state $z^u_{cm}, z^s_{cm}$ of a cell.

cell2fate describes time-dependent transcription rates, unspliced and spliced counts as the sum over multiple modules effects, as described previously in equations 10 to 12.

## 2.3 Parameter estimation

The original RNAvelocity method velocyto uses least-squares fitting of each parameter. scVelo and UniTVelo deploy maximum likelihood estimation with an Expectation Maximization algorithm. DeepVelo is based on backpropogation. VeloVAE, VeloVI and LatentVelo use backpropogation and mini-batch stochastic gradient descent for variational autoencoders in pytorch, thus enabling posterior parameter uncertainty. Similarly, Pyro-Velocity and cell2fate use stochastic variational inference in pyro.

## 2.4 Multiple dynamics

If an entire group of genes is not expressed at all in one cell type, but expressed in another, UniTVelo can account for such simple differences by assigning a different time to cells in one cell type than another. cell2fate, can more generally account for genes that are transcribed at different non-zero rates in different cell types, by similarly assigning a different time to different cell types, which will result in different modules being turned on in different cell types. Since velocyto, scVelo and veloVI estimate a separate time for each gene, they can also partially account for multiple dynamics. Specifically, a gene that is off in one cell type, but not in the other will simply be assigned a time of 0 for all cells of the latter cell type. However, if a gene is transcribed at different non-zero levels in two cell types, this cannot be accounted for by these models. DeepVelo, LatentVelo and veloVAE can account for multiple dynamics since rates are a function of gene expression or inferred latent state.

## 2.5 Stochastic fate decisions

At branching points the gene expression of a cell is not entirely predictive of its future. Instead, different transcription programs are turned on stochastically. This violates the assumptions of DeepVelo and LatentVelo that make dynamics a deterministic function of current expression. It also violates the assumptions of the cell2fate and UniTVelo methods, that have a transcription rate, as a deterministic function of time. VeloVAE can in principle account for such stochasticity as rates are not a function of gene expression or time, but instead depend on an inferred latent state. Similar to the case of multiple dynamics, the gene-specific time in velocyto, scVelo and veloVI can partially account for stochastic branching points. Specifically, a simple binary decision, where a gene is turned on or not stochastically at a branching point, can be fit with

either a non-zero or zero gene-specific time. However, in more complex cases, where a gene stochastically changes its transcription rate to different non-zero values, these models cannot describe the stochastic dynamics accurately. In Pyro-velocity, the switch on point of a gene does not only depend on time, but also on a Bernoulli variable, which can thus capture stochastic decisions, in which a gene is either turned on or not.

## 2.6 Mechanistic basis for switch time and rate parameters

In velocyto, scVelo, veloVI and pyro-velocity genes are assumed to switch on and off at two unique time points. This is only partially realistic, as the transcription rate of each gene, should change continuously in response to abundance of different transcription factors. This is the motivation for cell2fate's regulatory module formalism. In contrast, in DeepVelo and VeloVI rates change without dependence on a lower-level, mechanistically motivated structure. Finally, in LatentVelo rates are not modelled at all, since latent state velocity and time are inferred directly.

## 2.7 Low-rank modelling of gene switch times, transcription rates or counts

Co-regulation of genes by common transcription factors, results in a low-rank structure to observed dynamics, that can be modelled to increase statistical power. veloVI factorizes the time-matrix $T_{cg}$ for this purpose, using 10 dimensions by default. LatentVelo represents counts in a latent space of 20 dimensions and models the velocity of these latent states. cell2fate is unique in modelling the fundamental low-rank structure of transcription rates directly. A lower number of switch-times, and a low-rank structure for counts and velocities, results as a downstream result from this modelling choice.

## 2.8 Closed-form solution to count time evolution

velocyto, scvelo, veloVI and pyro-velocity are all based on solving the RNA velocity differential equations analytically, which is possible, because of their simple transcription rate functions. DeepVelo, veloVAE, LatentVelo use numerical approximations to solve their ODEs, which is required given their complex transcription rate functions. In particular, LatentVelo makes use of the "torchdiffeq" package for neural ODEs to compute gradients of inferred latent time points. DeepVelo and veloVAE use approximations as described previously in section 2.2. Cell2fate stands out as having both a complex transcription rate function and an anlalytical solution for the time evolution of spliced and unspliced counts. This is expected to improve performance by circumventing any numerical inaccuracies.

## 2.9 Raw Count Input

pyro-velocity and cell2fate are the only methods that use raw counts as an input. All other methods require preprocessing the data using total-count normalization, log-transformation and nearest-neighbour smoothing. These preprocessing steps are expected to introduce biases for lowly expressed genes (log-transformation) and rare cell-types (nearest-neighbour smoothing).

## 2.10 Negative Binomial Noise, Batch Correction, Ambient RNA, Detection Probabilities

Both LatentVelo and cell2fate include a batch variable in their model. Cell2fate stands out as the only method to model ambient RNA, detection probabilities and Negative Binomial overdispersion (all seperate for spliced and unspliced counts).

## 2.11 Regularization via Hierarchical Priors

Cell2fate makes use of the flexibility of the pyro-probabilistic programming language by using hierarchical priors for all cell and gene specific parameters, which is expected to provide parameter regularization in a principled manner.

# 3 Appendix

## 3.1 Derivation of transcription rate, spliced and unspliced counts as a function of time

We drop both subscripts and the summation over all modules for simplicity. Starting from our differential equation for the transcription rate:

$$\frac{d\alpha}{dt} = (\hat{\alpha} - \alpha)\lambda$$

This can be solved directly by integrating:

$$\int_{\alpha_0}^{\alpha} \frac{1}{(\hat{\alpha} - \alpha)\lambda} d\alpha = \int_{t_0}^{t} dt \tag{98}$$

To yield:

$$\ln(\hat{\alpha} - \alpha_0) - \ln(\hat{\alpha} - \alpha) = \lambda(t - t_0) \tag{99}$$

Defining:

$$\tau = t - t_0 \tag{100}$$

$$\delta^{\alpha} = \hat{\alpha} - \alpha_0 \tag{101}$$

And rearranging terms we obtain our solution for $\alpha$:

$$\alpha(t) = \alpha_0 + \delta^{\alpha}(1 - e^{-\lambda\tau}) \tag{102}$$

Substituting into the equation for unspliced counts:

$$\frac{du}{dt} = \alpha_0 + \delta^{\alpha}(1 - e^{-\lambda t}) - \beta u \tag{103}$$

We multiply both sides by $e^{\beta t}$ and solve this via integration:

$$e^{\beta t}\frac{du}{dt} + e^{\beta t}\beta u = e^{\beta t}\alpha_0 + e^{\beta t}\delta^{\alpha}(1 - e^{-\lambda t}) \tag{104}$$

$$\int_{t_0}^{t} \frac{d}{dt}\left(e^{\beta t}u\right) dt = \int_{t_0}^{t} e^{\beta t}\alpha_0 + e^{\beta t}\delta^{\alpha}(1 - e^{-\lambda t})dt \tag{105}$$

$$e^{\beta\tau}u = \frac{\delta^{\alpha}}{\beta}e^{\beta\tau} - \frac{\delta^{\alpha}}{\beta - \lambda}e^{-\lambda\tau}e^{\beta\tau} + \frac{\alpha_0}{\beta}e^{\beta\tau} + C \tag{106}$$

C is given by substituting the initial condition $(u_0, t_0)$:

$$C = \left(u_0 - \frac{\delta^{\alpha} + \alpha_0}{\beta} + \frac{\delta^{\alpha}}{\beta - \lambda}\right)e^{\beta t_0} \tag{107}$$

Substituting C and rearranging terms gives us our equation for unspliced counts:

$$u = u^0 e^{-\beta\tau} + \frac{\alpha^0}{\beta}(1 - e^{-\beta\tau}) + \frac{\delta^{\alpha}}{\beta} - \lambda(e^{-\beta\tau} - e^{-\lambda\tau})) \tag{108}$$

The solution for spliced counts is obtained in a similar manner. We first substitute our expression for unspliced counts, then multiply by $e^{\gamma t}$ and then integrate:

$$\int_{t_0}^{t} \frac{d}{dt}\left(e^{\gamma t}s\right) dt = \int_{t_0}^{t} \beta u_0 e^{-\beta t}e^{\gamma t} + \alpha_0(1 - e^{-\beta t})e^{-\gamma t} + \frac{\delta^{\alpha}\beta}{\beta - \lambda}(e^{-\beta t} - e^{-\lambda t})e^{\gamma t}dt \tag{109}$$

to obtain:

$$\mathrm{e}^{\gamma\tau}s = \frac{\beta u_0}{\gamma - \beta}e^{\beta\tau}e^{\gamma\tau} + \frac{\alpha^0}{\gamma}e^{\gamma\tau} - \frac{\alpha^0}{\gamma - \beta}e^{-\beta\tau}e^{\gamma\tau} + \frac{\delta^{\alpha}\beta}{(\beta - \lambda)(\gamma - \beta)}e^{-\beta\tau}e^{\gamma\tau} - \frac{\delta^{\alpha}\beta}{(\beta - \lambda)(\gamma - \lambda)}e^{-\lambda\tau}e^{\gamma\tau} + C \tag{110}$$

The initial condition is found at the point $(t_0, s_0)$ and after rearranging terms we obtain the solution for spliced counts.

## 3.2 Effect of mRNA detection probabilities on observed dynamics

We define observed spliced and unspliced counts as $s^{obs} = l_s \cdot s$ and $u^{obs} = l_u \cdot u$ respectively, i.e. $l_s$ and $l_u$ are detection probabilities for spliced and unspliced counts respectively.

For simplicity we make the approximation that $\lambda >> 1$. This means changes in the transcription rate do not happen gradually over time, but instead occur instantaneously. This means we can use the simpler equations from the scvelo model [1] and now multiply both sides by $l_u$ and $l_s$ respectively:

$$\frac{d(u \cdot l_u)}{dt} = l_u \cdot \alpha - \beta \cdot (l_u \cdot u) \tag{111}$$

$$\frac{d(s \cdot l_s)}{dt} = \beta \cdot (l_s \cdot u) - \gamma \cdot (l_s \cdot s) \tag{112}$$

Substituting expressions for observed counts we obtain:

$$\frac{du^{obs}}{dt} = l_u \cdot \alpha - \beta^{obs} \tag{113}$$

$$\frac{ds^{obs}}{dt} = \beta \cdot \frac{l_s}{l_u} u^{obs} - \gamma \cdot (s^{obs}) \tag{114}$$

This shows the importance of modelling detection probabilities. Otherwise, observed dynamics for spliced counts will appear to have a different splicing rate than unspliced counts by a factor of $\frac{l_u}{l_s}$ and the transcription rate will appear smaller by a factor $l_u$. This also implies that modelling relative detection probabilities between spliced and unspliced counts is enough, if we do not care about the absolute magnitude of the transcription rate for downstream analysis. Total-count normalization cannot remove this effect, since total counts can vary for biological reasons and not just due to detection probabilities.

## 3.3 Approximate time until steady-state spliced counts abundance

We aim to find the time at which spliced counts reach $r = 0.99$ of their maximal abundance, so $s(t) = \frac{r\alpha}{\gamma}$.

We assume $\lambda >> 1$ so that we can use this simple form for spliced counts, first suggested in the scvelo model [1]:

$$s(t) = s_0 e^{-\gamma\tau} + \frac{\alpha}{\gamma}(1 - e^{-\gamma\tau}) + \frac{\alpha - \beta u_0}{\gamma - \beta}(e^{-\gamma\tau} - e^{-\beta\tau}) \tag{115}$$

For most genes, $\beta > \gamma$ [15], so that at $\tau >> 1$, exponential terms with $\gamma$ will dominate the spliced counts equation:

$$\frac{r\alpha}{\gamma} \approx s_0 e^{-\gamma\tau} + \frac{\alpha}{\gamma}(1 - e^{-\gamma\tau}) + \frac{\alpha - \beta u_0}{\gamma - \beta}(e^{-\gamma\tau}) \tag{116}$$

Rearranging for $\tau$ and assuming $u_0 = s_0 = 0$:

$$\tau = \frac{-1}{\gamma} \ln\left(\frac{(r-1)}{\frac{\gamma}{\gamma - \beta} - 1}\right) \tag{117}$$

And substituting typical values of $\beta = 1\ \texttt{mol/h}$ and $\gamma = 0.2\ \ \mathrm{mol/h}$ :

$$\tau = 21.5\,\texttt{hours} \tag{118}$$

This can be seen as a general order of magnitude at which most genes will reach most of their maximal abundance. However, some genes will be much faster and others much slower, depending on their splicing and degradation rates. If we do not assume $u_0 = s_0 = 0$ and $\lambda >> 1$ this time will also depend on the $\alpha$ and $\lambda$ values for each gene.

## 3.4 Connection to non-negative matrix factorization

There is an interesting connection of our model to non-negative matrix factorization (NMF). In principle, the $g_{mg}$ values could be found with a simple NMF model, defined as:

$$S_{cg} = w_{cm} \cdot g_{mg}, \tag{119}$$

where $w_{cm}$ would take on different values between 0 and 1 depending on how close a cell is to the steady state for the module. For example, this prior could be appropriate:

$$w_{cm} \sim \texttt{Beta}(1, 1) \tag{120}$$

However, as we show in 3.3, how quickly a gene approaches its steady-state depends on the various rate parameters of a gene. So summarizing the similarity to the steady state with a single gene-shared parameter $w_{cm}$ effectively assumes that all genes have the same rate parameters, which is unrealistic. Hence, NMF will generally find a different set of $g_{mg}$ values. This also shows the problem with using dimensionality reduction methods as a preprocessing step as typically done in "knn-smoothing".

## 3.5 Potential Extensions

### 3.5.1 Linking transcription rate modules by a regulatory model

Currently, the effect on transcription rates by each module $A_{mg}$ is given by a prior distribution that does not share information across time steps (i.e. equations 58 to 60) and the active windows of modules can overlap. Instead,

we could constrain modules to be switched off when the next module switches on by inferring a fixed time gap for all modules, termed $\delta^\tau$, and constraining swtich ON times to be the previous module's switch off times:

$$\delta^\tau \sim T^{max} \cdot \texttt{Gamma}(\alpha = 20, \beta = 20 * M) \tag{121}$$

$$T_{mOFF} = m \times \delta^\tau \tag{122}$$

$$T_{mON} = \begin{cases} 0 & \text{if } m = 1 \\ T_{(m-1)OFF} & \text{if m} > 1 \end{cases}$$

The first module would then have an independent prior distribution as before in equation 58, but all other modules could be linked to the previous one by the spliced count abundance at their point of activation, termed $s_m^0$ i.e.

$$A_{mg} = \texttt{Function}(s_m^0) \text{ if m} > 0, \tag{124}$$

where `Function` can be any differentiable function to express regulatory relationships between genes, including non-linear or non-integrable functions. Crucially, $s_m^0$ can be calculated by substituting the switch ON time of the module into equation 34 and only summing up to the previous module. In this way, we obtain a recursive differentiable function for spliced and unspliced counts that can be optimized in pyro.

#### 3.5.2 Extension to model stochastic fate decisions

To model seemingly stochastic bifurcations in which cells with identical expression at one point can follow different future transcriptional paths, we suggest to infer a latent discrete parameter, termed $J_c$, for each cell:

$$J_c \sim \texttt{Binomial}(p = 0.5) \tag{125}$$

and include this parameter in the function that calculates the next transcriptional module for each cell:

$$A_{mgc} = \texttt{Function}(s_m^0, J_c) \text{ if m} > 0 \tag{126}$$

$J_c$ can be interpreted as stochastic transcriptional effects, as well as the effects of unobserved influences on gene regulation, including, for example, the methylation state of regulatory regions or the effect of external signals on the activation state of transcription factors.

#### 3.5.3 Extension to variable splicing and degradation rates

An approximate solutions to introduce time-varying splicing and degradation rates would be to have different rates for each module and state. For example, we could change $\beta_m$ to $\beta_{mgi}$, so that for unspliced counts we have:

$$\mathrm{u}_g(T_c) = \sum_M u_{mg}(T_c) = \sum_M (u^0_{mgi} e^{-\beta_{mgi}\tau_{cmi}} + \frac{\alpha^0_{mgi}}{\beta_{mgi}}(1 - e^{-\beta_{mgi}\tau_{cmi}}) + \frac{\delta^\alpha_{mgi}}{\beta_{mgi} - \lambda_{mi}}(e^{-\beta_{mgi}\tau_{cmi}} - e^{-\lambda_{mi}\tau_{cmi}}))$$

Since each module-specific splicing rate would only apply to counts produced by that module, the splicing rate for each gene at each point in time would effectively be an average, weighted by the counts of each module at each point in time.

$$\beta_g(T_c) = \sum_M \frac{u_{mg}(T_c) \cdot \beta_{mgi}}{u_g(T_c)} \tag{127}$$

Time-varying degradation rates would follow a similar reasoning.

### 3.6 List of all components in the cell2fate generative model

$$T^{max} \sim \texttt{Gamma}(\mu = 100, \sigma = 100) \tag{128}$$

$$t^{loc} \sim \texttt{Gamma}(\alpha = 1, \beta = \frac{1}{0.5}) \tag{129}$$

$$t^{scale} \sim \texttt{Gamma}(\alpha = 1, \beta = \frac{1}{0.25}) \tag{130}$$

$$t_c \sim \texttt{Gamma}(\mu = t^{loc}, \sigma = t^{scale}) \tag{131}$$

$$T_c = t_c * T^{max} \tag{132}$$

$$\delta^\tau_{mON} \sim \begin{cases} 0 & \text{if } m = 0 \\ T^{max} \cdot \texttt{Gamma}(\alpha = 20, \beta = 20 * M) & \text{otherwise} \end{cases}$$

$$T_{mON} = \sum_{m'=0}^{m-1} \delta^\tau_{m'ON} \tag{133}$$

$$\delta^\tau_{mOFF} \sim \texttt{Exponential}\left(\frac{M}{T^{max}}\right) \tag{134}$$

$$T_{mOFF} = T_{mON} + \delta^\tau_{mOFF} \tag{135}$$

$$i = \begin{cases} ON & \text{if } T_c > T_{mON} \text{ and } T_c < T_{mOFF} \\ OFF & \text{otherwise} \end{cases}$$

$$\alpha^T_{mgi} = \begin{cases} A_{mgON} & \text{if } i = ON \\ 0 & \text{if } i = OFF, \end{cases}$$

$\alpha_g(T_c) = \sum_m \alpha_{mg}(T_c) = \sum_M (\alpha^0_{mgi} + \delta^\alpha_{mgi}(1 - e^{-\lambda_{mi}\tau_{cmi}}))$

$\mathrm{u}_g(T_c) = \sum_m u_{mg}(T_c) = \sum_m (u^0_{mgi} e^{-\beta_g \tau_{cmi}} + \dfrac{\alpha^0_{mgi}}{\beta_g}(1 - e^{-\beta_g \tau_{cmi}}) + \dfrac{\delta^\alpha_{mgi}}{\beta_g - \lambda_{mi}}(e^{-\beta_g \tau_{cmi}} - e^{-\lambda_{mi}\tau_{cmi}}))$

$\mathrm{s}_g(T_c) = \sum_m s_{mg}(T_c) = \sum_m (s^0_{mgi} e^{-\gamma_g \tau_{cmi}} + \dfrac{\alpha^0_{mgi}}{\gamma_g}(1 - e^{-\gamma_g \tau_{cmi}}) + \dfrac{\alpha^0_{mgi} - \beta_g u^0_{mgi}}{\gamma_g - \beta_g}(e^{-\gamma_g \tau_{cmi}} - e^{-\beta_g \tau_{cmi}}) + \dfrac{\delta^\alpha_{mgi}\beta_g}{(\beta_g - \lambda_{mi})(\gamma_g - \beta_g)}(e^{-\beta_g \tau_{cmi}} - e^{-\gamma_g \tau_{cmi}}) + \dfrac{\delta^\alpha_{mgi}\beta_g}{(\beta_g - \lambda_{mi})(\gamma_g - \lambda_{mi})}(e^{-\lambda_{mi}\tau_{cmi}} - e^{-\gamma_g \tau_{cmi}}))$

$$\tau_{cmi} = T_c - T_{mi} \tag{137}$$
$$\delta^\alpha_{mgi} = \alpha^T_{mgi} - \alpha^0_{mgi} \tag{138}$$
$$\alpha^0_{mgON} = 0 \tag{139}$$
$$u^0_{mgON} = 0 \tag{140}$$
$$s^0_{mgON} = 0 \tag{141}$$
$$\alpha^0_{mgOFF} = \hat{\alpha}_{mg}(T_{mOFF}) \tag{142}$$
$$u^0_{mgOFF} = u_{gm}(T_{mOFF}) \tag{143}$$
$$s^0_{mgOFF} = s_{gm}(T_{mOFF}) \tag{144}$$
$$x^B_{cgj} = (u_{cg}, s_{cg}) \tag{145}$$
$$x^M_{cgj} = l_{cgj} * (H_{ce} * s_{egj} + x^B_{cgj}), \tag{146}$$
$$X_{cgj} \sim \texttt{NegBinom}(\mu = x^M_{cgj}, \alpha = a_{gj}), \tag{147}$$

$$\alpha^\gamma \sim \texttt{Gamma}(\alpha = 20, \beta = \frac{20}{5}) \tag{148}$$

$$\mu^\gamma \sim \texttt{Gamma}(\alpha = 10, \beta = \frac{10}{1}) \tag{149}$$

$$\gamma_g \sim \texttt{Gamma}(\alpha = \alpha^\gamma, \beta = \frac{\alpha^\gamma}{\mu^\gamma}) \tag{150}$$

$$\alpha^\beta \sim \texttt{Gamma}(\alpha = 20, \beta = \frac{20}{5}) \tag{151}$$

$$\mu^\beta \sim \texttt{Gamma}(\alpha = 10, \beta = \frac{10}{1}) \tag{152}$$

$$\beta_g \sim \texttt{Gamma}(\alpha = \alpha^\beta, \beta = \frac{\alpha^\beta}{\mu^\beta}) \tag{153}$$

$$\mu^\lambda \sim \texttt{Gamma}(\mu = 1, \sigma = 0.33) \tag{154}$$

$$\sigma^\lambda \sim \texttt{Gamma}(\mu = 0.33, \sigma = 0.1) \tag{155}$$

$$\lambda_m \sim \texttt{Gamma}(\mu = \mu^\lambda, \sigma = \sigma^\lambda) \tag{156}$$

$$\lambda_{mi} \sim \texttt{Gamma}(\mu = \lambda_m, \sigma = \lambda_m * 0.05) \tag{157}$$

$$A_{mg} = g_{mg} \cdot \gamma_g \tag{158}$$

$$g_g \sim \texttt{Gamma}(\alpha = 1, \beta = 1) \tag{159}$$

$$g_{mg} \sim \texttt{Gamma}(\alpha = \frac{h}{M}, \beta = \frac{1}{g_g}) \tag{160}$$

$$h = 3 \tag{161}$$

$$a_{gj} = \frac{1}{E_g^2} \tag{162}$$

$$E_{gj} \sim \texttt{Exponential}(\phi) \tag{163}$$

$$\phi_j \sim \texttt{Gamma}(\mu = 3, \sigma = 1) \tag{164}$$

$$l_e \sim \texttt{Beta}(\alpha = 1, \beta = 1) \tag{165}$$

$$l_c \sim \texttt{Gamma}(\alpha = 10, \beta = \frac{10}{H_{ce} * l_e}) \tag{166}$$

$$l_j \sim \texttt{Gamma}(\alpha = 100, \beta = \frac{100}{1}), \tag{167}$$

$$l_{gj} \sim \texttt{Gamma}(\alpha = 200, \beta = \frac{200}{1}) \tag{168}$$

$$l_{cgj} = l_c \cdot l_j \cdot l_{gj} \tag{169}$$

$$s_e \sim \texttt{Gamma}(\mu = 0.005, \sigma = 0.00005) \tag{170}$$

$$b_{ej} \sim \texttt{Exponential}(\lambda^b = 9) \tag{171}$$

$$a^s_{ej} = \frac{1}{b^2_{ei}} \tag{172}$$

$$s_{egj} \sim \texttt{Gamma}(\alpha = a^s_{ej}, \beta = \frac{a^s_{ej}}{s_e}) \tag{173}$$

# References


[1] Volker Bergen, Marius Lange, Stefan Peidli, F Alexander Wolf, and Fabian J Theis. Generalizing rna velocity to transient cell states through dynamical modeling. *Nature biotechnology*, 38(12):1408–1414, 2020.

[2] Volker Bergen, Ruslan A Soldatov, Peter V Kharchenko, and Fabian J Theis. Rna velocity—current challenges and future perspectives. *Molecular systems biology*, 17(8):e10282, 2021.

[3] Eli Bingham, Jonathan P Chen, Martin Jankowiak, Fritz Obermeyer, Neeraj Pradhan, Theofanis Karaletsos, Rohit Singh, Paul Szerlip, Paul Horsfall, and Noah D Goodman. Pyro: Deep universal probabilistic programming. *The Journal of Machine Learning Research*, 20(1):973–978, 2019.

[4] Zhanlin Chen, William C King, Aheyon Hwang, Mark Gerstein, and Jing Zhang. Deepvelo: Single-cell transcriptomic deep velocity field learning with neural ordinary differential equations. *Science Advances*, 8(48):eabq3745, 2022.

[5] Spencer Farrell, Madhav Mani, and Sidhartha Goyal. Inferring single-cell transcriptomic dynamics with structured dynamical representations of rna velocity. *Bulletin of the American Physical Society*, 2023.

[6] Mingze Gao, Chen Qiao, and Yuanhua Huang. Unitvelo: temporally unified rna velocity reinforces single-cell trajectory inference. *Nature Communications*, 13(1):6586, 2022.

[7] Adam Gayoso, Philipp Weiler, Mohammad Lotfollahi, Dominik Klein, Justin Hong, Aaron M Streets, Fabian J Theis, and Nir Yosef. Deep generative modeling of transcriptional dynamics for rna velocity analysis in single cells. *bioRxiv*, pages 2022–08, 2022.

[8] Gennady Gorin, Meichen Fang, Tara Chari, and Lior Pachter. Rna velocity unraveled. *PLOS Computational Biology*, 18(9):e1010492, 2022.

[9] Yichen Gu, David Blaauw, and Joshua D Welch. Bayesian inference of rna velocity from multi-lineage single-cell data. *bioRxiv*, pages 2022–07, 2022.

[10] Tobias Jahnke and Wilhelm Huisinga. Solving the chemical master equation for monomolecular reaction systems analytically. *Journal of mathematical biology*, 54:1–26, 2007.

[11] Diederik P Kingma and Jimmy Ba. Adam: A method for stochastic optimization. *arXiv preprint arXiv:1412.6980*, 2014.

[12] Gioele La Manno, Ruslan Soldatov, Amit Zeisel, Emelie Braun, Hannah Hochgerner, Viktor Petukhov, Katja Lidschreiber, Maria E Kastriti, Peter Lönnerberg, Alessandro Furlan, et al. Rna velocity of single cells. *Nature*, 560(7719):494–498, 2018.

[13] Tiejun Li, Jifan Shi, Yichong Wu, and Peijie Zhou. On the mathematics of rna velocity i: theoretical analysis. *bioRxiv*, pages 2020–09, 2020.

[14] Qian Qin, Eli Bingham, Gioele La Manno, David M Langenau, and Luca Pinello. Pyro-velocity: Probabilistic rna velocity inference from single-cell data. *bioRxiv*, pages 2022–09, 2022.

[15] Michal Rabani, Joshua Z Levin, Lin Fan, Xian Adiconis, Raktima Raychowdhury, Manuel Garber, Andreas Gnirke, Chad Nusbaum, Nir Hacohen, Nir Friedman, et al. Metabolic labeling of rna uncovers principles of rna production and degradation dynamics in mammalian cells. *Nature biotechnology*, 29(5):436–442, 2011.

[16] Abhishek K Sarkar and Matthew Stephens. Separating measurement and expression models clarifies confusion in single cell rna-seq analysis. *BioRxiv*, 2020.

[17] Adam J Shaywitz and Michael E Greenberg. Creb: a stimulus-induced transcription factor activated by a diverse array of extracellular signals. *Annual review of biochemistry*, 68(1):821–861, 1999.

[18] Daniel Simpson, Håvard Rue, Andrea Riebler, Thiago G Martins, and Sigrunn H Sørbye. Penalising model component complexity: A principled, practical approach to constructing priors. *Statistical science*, 32(1):1–28, 2017.

[19] Ty C Voss and Gordon L Hager. Dynamic regulation of transcriptional states by chromatin and transcription factors. *Nature Reviews Genetics*, 15(2):69–81, 2014.

[20] Amit Zeisel, Wolfgang J Köstler, Natali Molotski, Jonathan M Tsai, Rita Krauthgamer, Jasmine Jacob-Hirsch, Gideon Rechavi, Yoav Soen, Steffen Jung, Yosef Yarden, et al. Coupled pre-mrna and mrna dynamics unveil operational strategies underlying transcriptional responses to stimuli. *Molecular systems biology*, 7(1):529, 2011.
