# Supplementary material for: Cell2fate infers RNA velocity modules to improve cell fate prediction
Source: Nat Methods. 2025 Mar 3;22(4):698–707. doi: 10.1038/s41592-025-02608-3 (PMC11978503; doi:10.1038/s41592-025-02608-3)
Supplement: Supplementary file 2 — Reporting Summary [file 41592_2025_2608_MOESM2_ESM.pdf]

nature portfolio

Corresponding author(s): Omer Bayraktar

Last updated by author(s): 18/10/24

Reporting Summary

Nature Portfolio wishes to improve the reproducibility of the work that we publish. This form provides structure for consistency and transparency in reporting. For further information on Nature Portfolio policies, see our Editorial Policies and the Editorial Policy Checklist.

Statistics

For all statistical analyses, confirm that the following items are present in the figure legend, table legend, main text, or Methods section.

n/a	Confirmed	
☐	☒	The exact sample size (*n*) for each experimental group/condition, given as a discrete number and unit of measurement
☐	☒	A statement on whether measurements were taken from distinct samples or whether the same sample was measured repeatedly
☒	☐	The statistical test(s) used AND whether they are one- or two-sided<br>*Only common tests should be described solely by name; describe more complex techniques in the Methods section.*
☒	☐	A description of all covariates tested
☐	☒	A description of any assumptions or corrections, such as tests of normality and adjustment for multiple comparisons
☐	☒	A full description of the statistical parameters including central tendency (e.g. means) or other basic estimates (e.g. regression coefficient) AND variation (e.g. standard deviation) or associated estimates of uncertainty (e.g. confidence intervals)
☒	☐	For null hypothesis testing, the test statistic (e.g. *F*, *t*, *r*) with confidence intervals, effect sizes, degrees of freedom and *P* value noted<br>*Give P values as exact values whenever suitable.*
☐	☒	For Bayesian analysis, information on the choice of priors and Markov chain Monte Carlo settings
☒	☐	For hierarchical and complex designs, identification of the appropriate level for tests and full reporting of outcomes
☐	☒	Estimates of effect sizes (e.g. Cohen's *d*, Pearson's *r*), indicating how they were calculated

Our web collection on statistics for biologists contains articles on many of the points above.

Software and code

Policy information about availability of computer code

Data collection

The single-nucleus RNAseq data was processed using the open-source StarSolo method, version 2.7.9a with the velocyto option enabled.
Available at: https://github.com/alexdobin/STAR/blob/master/docs/STARsolo.md

Data analysis

Analysis of RNAseq data was performed with the cell2fate method available here:
https://github.com/BayraktarLab/cell2fate

Results from the cell2fate method can be reproduced with the notebooks in this repository:
github.com/AlexanderAivazidis/cell2fate_notebooks
Benchmarking results for all methods, as well as robustness analysis and comparison to real developmental age was done with notebooks in this repository:
github.com/AlexanderAivazidis/fate_benchmarking

We used a computing environment with the following publically available python packages:

Name	Version
_libgcc_mutex	0.1
_openmp_mutex	4.5
absl-py	1.2.0
aiohttp	3.8.1
aiosignal	1.2.0
anndata	0.8.0
appdirs	1.4.4

argon2-cffi	21.3.0
argon2-cffi-bindings	21.2.0
arpack	3.7.0
asttokens	2.0.5
astunparse	1.6.3
async-timeout	4.0.2
attrs	22.1.0
backcall	0.2.0
beautifulsoup4	4.11.1
bioservices	1.10.0
blas	1.0
bleach	5.0.1
bottleneck	1.3.5
brotlipy	0.7.0
c-ares	1.18.1
ca-certificates	2022.9.14
cachetools	5.2.0
cell2fate	0.1a0
cell2location	0.1
certifi	2022.9.14
cffi	1.15.1
charset-normalizer	2.1.0
chex	0.1.4
click	8.1.3
colorama	0.4.5
colorlog	6.6.0
commonmark	0.9.1
cryptography	35.0.0
cycler	0.11.0
debugpy	1.6.2
decorator	5.1.1
defusedxml	0.7.1
dm-tree	0.1.7
docrep	0.3.2
easydev	0.12.0
einops	0.4.1
entrypoints	0.4
et-xmlfile	1.1.0
etils	0.6.0
executing	0.9.1
fastcluster	1.2.6
fastjsonschema	2.16.1
flatbuffers	22.12.6
flax	0.5.0
fonttools	4.34.4
freetype	2.10.4
frozenlist	1.3.1
fsspec	2022.7.1
future	0.18.2
gast	0.4.0
gevent	21.8.0
glpk	4.65
gmp	6.2.1
google-auth	2.9.1
google-auth-oauthlib	0.4.6
google-pasta	0.2.0
greenlet	1.1.1
grequests	0.6.0
grpcio	1.47.0
gseapy	0.12.1
h5py	3.7.0
html5lib	1.1
icu	58.2
idna	3.3
igraph	0.9.10
importlib-metadata	3.10.0
importlib-resources	5.9.0
intel-openmp	2021.4.0
iprogress	0.4
ipykernel	6.15.1
ipython	8.4.0
ipython-genutils	0.2.0
ipywidgets	7.7.1
jax	0.3.15
jaxlib	0.3.15
jedi	0.18.1

jinja2	3.1.2
joblib	1.1.0
jpeg	9e
jsonschema	4.9.1
jupyter	1.0.0
jupyter-client	7.3.4
jupyter-console	6.4.4
jupyter-core	4.11.1
jupyterlab-pygments	0.2.2
jupyterlab-widgets	1.1.1
keras	2.11.0
kiwisolver	1.4.4
lcms2	2.12
ld_impl_linux-64	2.38
leidenalg	0.8.10
libblas	3.9.0
libcblas	3.9.0
libclang	14.0.6
libev	4.33
libffi	3.3
libgcc-ng	12.1.0
libgfortran-ng	7.5.0
libgfortran4	7.5.0
libhwloc	2.8.0
liblapack	3.9.0
libpng	1.6.37
libstdcxx-ng	12.1.0
libtiff	4.2.0
libuv	1.40.0
libwebp-base	1.2.2
libxml2	2.9.14
libxslt	1.1.35
libzlib	1.2.12
llvm-openmp	14.0.4
llvmlite	0.39.0
loompy	3.0.7
lxml	4.9.1
lz4-c	1.9.3
markdown	3.3.4
markupsafe	2.1.1
matplotlib	3.5.2
matplotlib-base	3.4.3
matplotlib-inline	0.1.3
metis	5.1.0
mistune	0.8.4
mkl	2021.4.0
mkl-service	2.4.0
mkl_fft	1.3.1
mkl_random	1.2.2
mpfr	4.1.0
msgpack	1.0.4
multidict	6.0.2
multipledispatch	0.6.0
natsort	8.1.0
nbclient	0.6.6
nbconvert	6.5.0
nbformat	5.4.0
ncurses	6.3
nest-asyncio	1.5.5
networkx	2.8.5
notebook	6.4.12
numba	0.56.0
numexpr	2.8.3
numpy	1.21.4
numpy-groupies	0.9.17
numpyro	0.10.0
oauthlib	3.2.0
olefile	0.46
opencv-python	4.6.0.66
openpyxl	3.0.10
openssl	1.1.1q
opt-einsum	3.3.0
optax	0.1.3
packaging	21.3
pandas	1.4.2
pandocfilters	1.5.0

parso	0.8.3
patsy	0.5.2
pexpect	4.8.0
pickleshare	0.7.5
pillow	9.2.0
pip	22.1.2
prometheus-client	0.14.1
prompt-toolkit	3.0.30
protobuf	3.19.6
psutil	5.9.1
ptyprocess	0.7.0
pure-eval	0.2.2
pyasn1	0.4.8
pyasn1-modules	0.2.8
pycparser	2.21
pydeprecate	0.3.1
pygments	2.12.0
pynndescent	0.5.7
pyopenssl	22.0.0
pyparsing	3.0.9
pyro-api	0.1.2
pyro-ppl	1.8.1
pyrsistent	0.18.1
pysocks	1.7.1
python	3.9.12
python-dateutil	2.8.2
python-graphviz	0.20.1
python-igraph	0.9.11
python_abi	3.9
pytorch-lightning	1.5.10
pytz	2022.1
pyyaml	6.0
pyzmq	23.2.0
qtconsole	5.4.0
qtpy	2.3.0
readline	8.1.2
requests	2.28.1
requests-oauthlib	1.3.1
requests_cache	0.4.13
rich	12.3.0
rsa	4.9
scanpy	1.9.1
scikit-learn	1.1.1
scipy	1.8.0
scvelo	0.2.4
scvi-tools	0.16.1
seaborn	0.11.2
send2trash	1.8.0
session-info	1.0.0
setuptools	59.5.0
six	1.16.0
soupsieve	2.3.2.post1
sqlite	3.39.0
stack-data	0.3.0
statsmodels	0.13.2
stdlib-list	0.8.0
suds-community	1.1.2
suitesparse	5.10.1
tbb	2021.5.0
tensorboard	2.11.0
tensorboard-data-server	0.6.1
tensorboard-plugin-wit	1.8.1
tensorflow	2.11.0
tensorflow-estimator	2.11.0
tensorflow-io-gcs-filesystem	0.28.0
termcolor	2.1.1
terminado	0.15.0
texttable	1.6.4
threadpoolctl	3.1.0
tinycss2	1.1.1
tk	8.6.12
toolz	0.12.0
torch	1.11.0
torchmetrics	0.9.3
tornado	6.2
tqdm	4.64.0

traitlets	5.3.0
txnburst	0.0.0
typing-extensions	4.3.0
tzdata	2022a
umap-learn	0.5.3
unitvelo	0.2.5
urllib3	1.26.11
wcwidth	0.2.5
webencodings	0.5.1
werkzeug	2.2.1
wheel	0.37.1
widgetsnbextension	3.6.1
wrapt	1.14.1
xmltodict	0.13.0
xz	5.2.5
yarl	1.8.1
zipp	3.8.1
zlib	1.2.12
zope.event	4.5.0
zope.interface	5.4.0
zstd	1.4.9

Additional algorithms run in our study are listed in the following together with their version number:

scvelo_dynamical
v0.2.5
scvelo_stochastic
v0.2.5
pyroVelocity_model1
0.1.0
veloVAE
n.a.
UniTVelo_independent
0.2.5
UniTVelo_unified
0.2.5
DeepVelo
0.2.5-rc.1
pyroVelocity_model2
0.1.0
VeloVI
0.1.1
CellRank
v.2.0.6
MOFA
v0.1
ProBound
1.4.0

For manuscripts utilizing custom algorithms or software that are central to the research but not yet described in published literature, software must be made available to editors and reviewers. We strongly encourage code deposition in a community repository (e.g. GitHub). See the Nature Portfolio guidelines for submitting code & software for further information.

Data

Policy information about availability of data

All manuscripts must include a data availability statement. This statement should provide the following information, where applicable:

- Accession codes, unique identifiers, or web links for publicly available datasets
- A description of any restrictions on data availability
- For clinical datasets or third party data, please ensure that the statement adheres to our policy

Raw UMI counts and metadata in anndata format for all single cell and Visium data is available
for download on this portal: https://cell2fate.cog.sanger.ac.uk/browser.html
FASTQ files for the human brain single-nucleus and Visium data are available on ENA under this accession number: PRJEB79988.
Reference GRCh38 v1.2.0 that was used to process the FASTQ files is available for download here: https://www.ncbi.nlm.nih.gov/datasets/genome/GCF_000001405.26/

Human research participants

Policy information about studies involving human research participants and Sex and Gender in Research.

Reporting on sex and gender	The sex was assigned as male by the Human Developmental Biology Resource, UCL, UK (REC 23/LO/0312) from where the second trimester human fetal brain tissue was obtained.
Population characteristics	No further characteristics were provided.
Recruitment	Human embryo and fetal samples were obtained from the MRC and Wellcome-funded Human Developmental Biology Resource (HDBR, http:// www.hdbr.org), with appropriate maternal written consent and approval from the Fulham Research Ethics Committee (REC reference 18/LO/0822) and Newcastle & North Tyneside 1 Research Ethics Committee (REC reference 18/NE/0290). The HDBR is regulated by the UK Human Tissue Authority (HTA; www.hta.gov.uk) and operates in accordance with the relevant HTA Codes of Practice.
Ethics oversight	Human embryo and fetal samples were obtained from the MRC and Wellcome-funded Human Developmental Biology Resource (HDBR43, http:// www.hdbr.org), with appropriate maternal written consent and approval from the Newcastle and North Tyneside NHS Health Authority Joint Ethics Committee (08/H0906/21+5). The HDBR is regulated by the UK Human Tissue Authority (HTA; www.hta.gov.uk) and operates in accordance with the relevant HTA Codes of Practice.

Note that full information on the approval of the study protocol must also be provided in the manuscript.

Field-specific reporting

Please select the one below that is the best fit for your research. If you are not sure, read the appropriate sections before making your selection.

☒ Life sciences ☐ Behavioural & social sciences ☐ Ecological, evolutionary & environmental sciences

For a reference copy of the document with all sections, see nature.com/documents/nr-reporting-summary-flat.pdf

Life sciences study design

All studies must disclose on these points even when the disclosure is negative.

Sample size	No sample size calculation was performed, since we did not use a case control study design, so there were no comparisons between groups. Instead we demonstrated our computational method on 5 public and 1 new dataset, for which Visium spatial transcriptomics and single-nucleus RNA sequencing were applied to one tissue sample from the same donor.
Data exclusions	Data was excluded during quality control of the single-nucleus RNAseq data, using default recommended count thresholds in the scanpy python processing pipeline: min_genes=200, min_cells=3, n_genes_by_counts = 2500, pct_counts_mt = 5
Replication	We did not replicate any experiments, since this is not generally needed for single-cell RNA sequencing, which is a very reproducible assay.
Randomization	There are no comparisons between experimental groups in our study hence no need for randomization.
Blinding	There are no comparisons between experimental groups in our study hence no need for blinding.

Reporting for specific materials, systems and methods

We require information from authors about some types of materials, experimental systems and methods used in many studies. Here, indicate whether each material, system or method listed is relevant to your study. If you are not sure if a list item applies to your research, read the appropriate section before selecting a response.

Materials & experimental systems

n/a	Involved in the study	
☒	☐	Antibodies
☒	☐	Eukaryotic cell lines
☒	☐	Palaeontology and archaeology
☒	☐	Animals and other organisms
☒	☐	Clinical data
☒	☐	Dual use research of concern

Methods

n/a	Involved in the study	
☒	☐	ChIP-seq
☒	☐	Flow cytometry
☒	☐	MRI-based neuroimaging
